# Supplementary material for: sVEGFR1 up-regulation via EGR1 impairs vascular repair in SFTSV-induced hemorrhage
Source: EMBO Rep. 2025 Aug 11;26(18):4477–502. doi: 10.1038/s44319-025-00541-2 (PMC12457690; doi:10.1038/s44319-025-00541-2)
Supplement: Supplementary file 1 — Appendix [file 44319_2025_541_MOESM1_ESM.pdf]

Contents:

Appendix Figure S1---2

Appendix Figure S2---3

Appendix Figure S3---4

Appendix Figure S4---5

Appendix Figure S5---6-7

Appendix Figure S6---8

Appendix Figure S7---9

Appendix Table S1---10-12

Appendix Figure S1

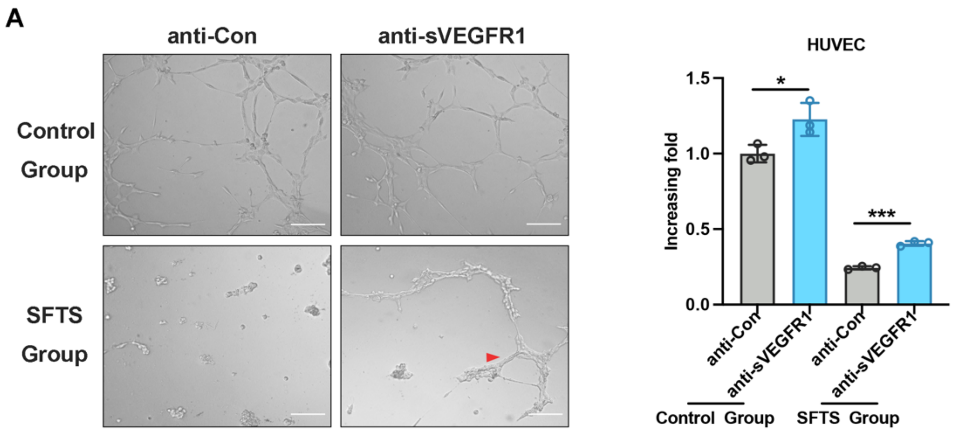

Appendix Figure S1. sVEGFR1 depletion rescues tube formation impairments.

(**A**) Sera from healthy individuals and SFTS patients were placed under UV light for 30 min to inactivate the virus, and then pretreated with a sVEGFR1 neutralizing antibody-conjugated or control antibody-conjugated magnetic beads to remove sVEGFR1. HUVECs were introduced into the Matrigel in the presence of pretreated-serum from either healthy individuals or SFTS patients, and tube formation of HUVECs was examined at 12 h after treatment. Representative capillary tubule structures were shown. Red arrowheads indicated tube elongation. Scale bar = 50 µm. Bar graph on the right represent the fold change of tubule formation. n = 3 biological replicates. Statistical significance was determined by two-tailed unpaired *Student t*-test. (Control: **P* = 0.0342; SFTS: ****P* = 0.0010)

Data information: Data shown were *Mean* ± *SD* of three biological replicates. (*, *P* < 0.05; ***, *P* < 0.001)

Appendix Figure S2

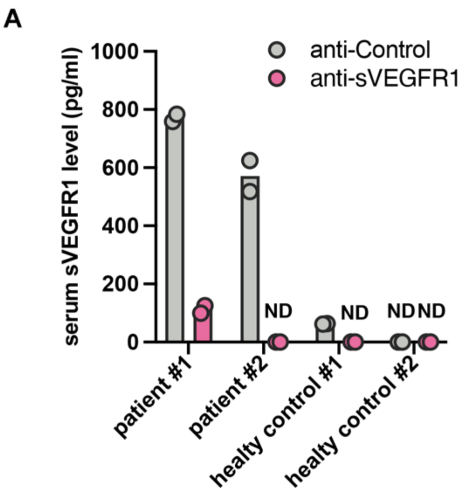

Appendix Figure S2. The efficiency of sVEGFR1 removal.

(**A**) Sera from healthy individuals and SFTS patients were placed under UV light for 30 min to inactivate the virus, and then pretreated with a sVEGFR1 neutralizing antibody-conjugated or control antibody-conjugated magnetic beads to remove sVEGFR1. sVEGFR1 in sera were measured by capture ELISA. ND, Not Detected. n = 2 technical replicates.

Appendix Figure S3

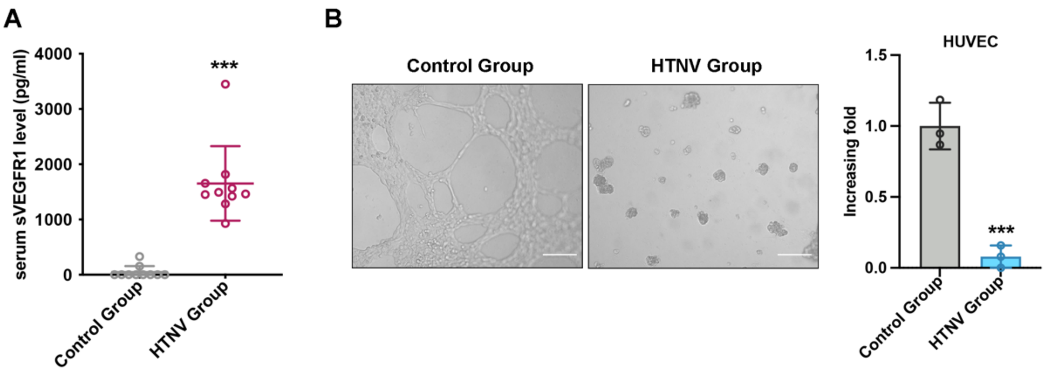

Appendix Figure S3. HTNV infection up-regulates sVEGFR1 expression in HFRS patients and HTNV induced angiogenesis dysfunction *in vitro*.

(**A**) Blood samples were obtained from 10 hemorrhagic fever with renal syndrome (HFRS) patients and 10 healthy individuals as controls. The serum level of sVEGFR1 was measured by capture ELISA. n = 10 biological replicates for each group. Statistical significance was determined by two-tailed unpaired *Student t*-test. (***$P < 0.0001$)

(**B**) HUVECs were introduced into Matrigel in the presence of sera derived from healthy individuals and HFRS patients. Each serum was placed under UV light for 30 min to inactivate residual virus. Tube formation of HUVECs was examined at 12 h after treatment. Representative capillary tubule structures were shown. Bar graph on the right represent the fold change of tubule formation. Scale bar = 50 μm. n = 3 biological replicates. Statistical significance was determined by two-tailed unpaired *Student t*-test. (***$P = 0.0009$)

Data information: Data shown were *Mean* ± *SD* of at least three biological replicates. (***, $P < 0.001$)

Appendix Figure S4

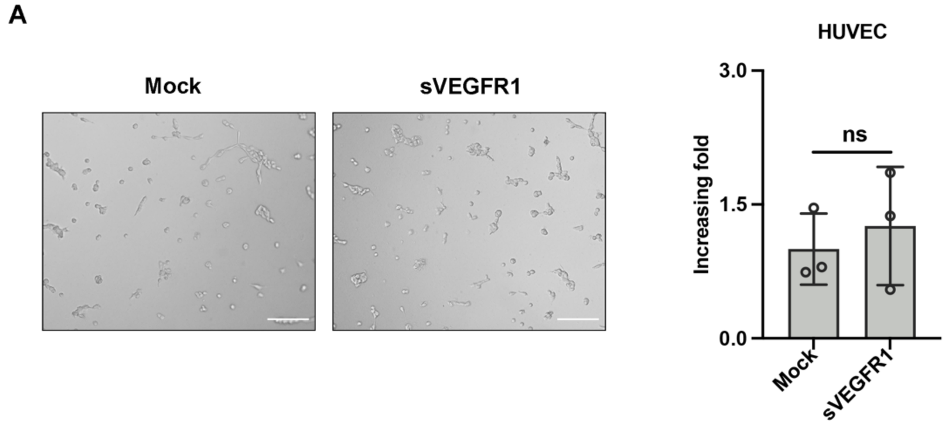

Appendix Figure S4.

(**A**) HUVECs were introduced into the Matrigel in the absence of human serum, following addition with or without exogenous recombinant sVEGFR1 (10 ng/ml). Tube formation of HUVECs was examined at 12 h after treatment. Scale bar = 50 µm. Bar graph on the right represent the fold change of tubule formation. n = 3 biological replicates. Statistical significance was determined by two-tailed unpaired *Student t*-test. (ns = 0.5944)

Data information: Data shown were *Mean* ± *SD* of three biological replicates. (ns, $P > 0.05$)

Appendix Figure S5

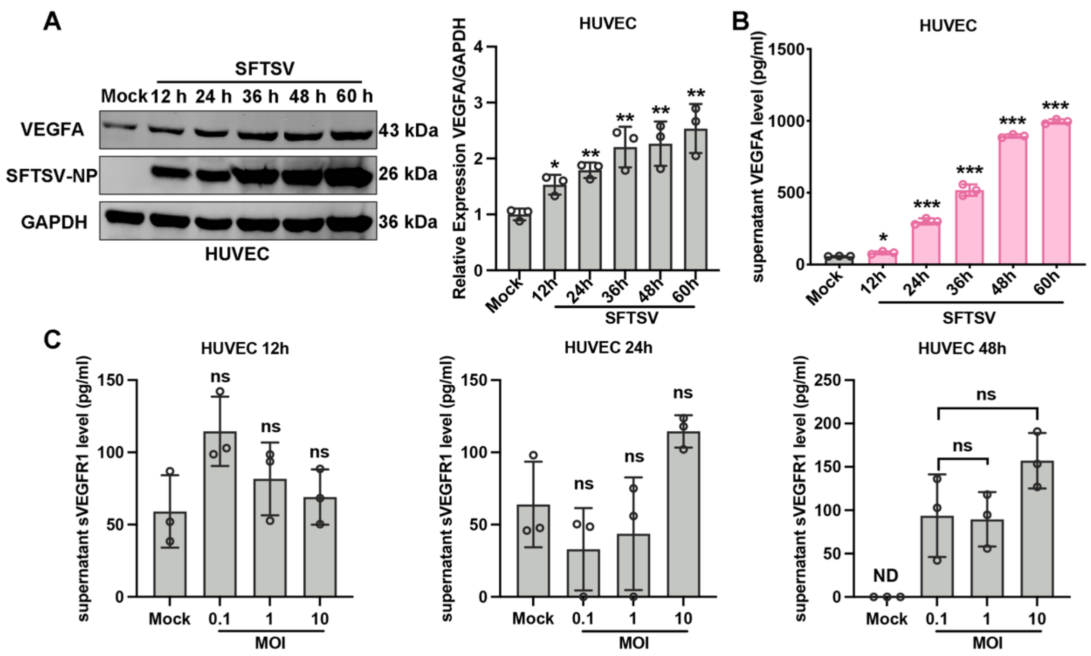

Appendix Figure S5. SFTSV infection up-regulates VEGFA expression, but does not affect sVEGFR1 expression in endothelial cells.

(**A**) HUVECs were infected with SFTSV (MOI = 1) for 12, 24, 36, 48 and 60 h. Intracellular VEGFA protein was analyzed by Western blotting (left). The VEGFA protein expression relative to GAPDH was shown (right). n = 3 biological replicates. Statistical significance was determined by two-tailed unpaired *Student t*-test. (**P* = 0.0109, ***P* = 0.0014, ***P* = 0.0053, ***P* = 0.0059, ***P* = 0.0041)

(**B**) HUVECs were infected with SFTSV (MOI = 1) for 12, 24, 36, 48 and 60 h. The secreted VEGFA in cell supernatant was measured by capture ELISA. n = 3 biological replicates. Statistical significance was determined by two-tailed unpaired *Student t*-test. (**P* = 0.0152, ****P* < 0.0001, ****P* < 0.0001, ****P* < 0.0001, ****P* < 0.0001)

(**C**) HUVECs were infected with SFTSV for 12, 24 and 48 h at various MOIs (MOI = 0.1, 1, and 10). sVEGFR1 in cell supernatant was measured by capture ELISA. ND, Not Detected. n = 3 biological replicates. Statistical significance was determined by two-tailed unpaired *Student t*-test. (12 h: ns = 0.0503, 0.3329, 0.6139; 24 h: ns = 0.2621, 0.5133, 0.0503; 48 h: ns =0.1282, 0.9053)

Data information: Data shown were *Mean* ± *SD* of three biological replicates. (ns, $P > 0.05$; *, $P < 0.05$; **, $P < 0.01$; ***, $P < 0.001$)

Appendix Figure S6

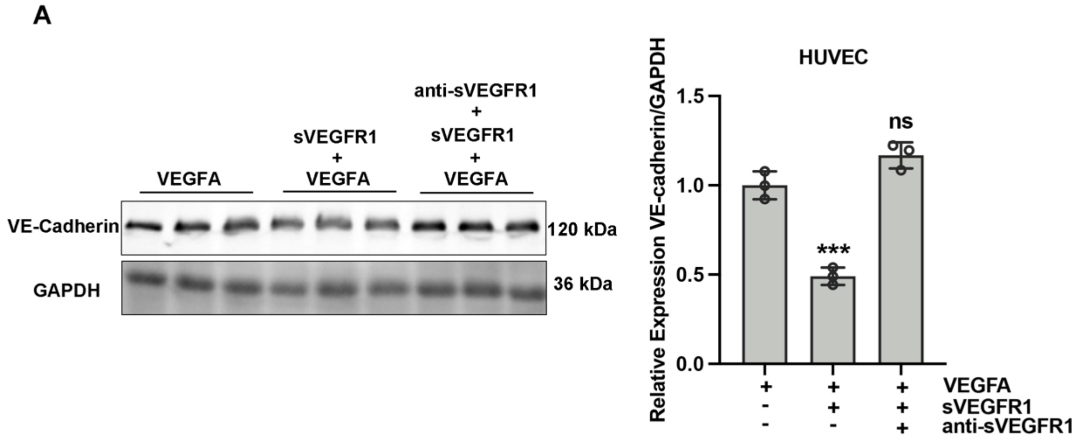

Appendix Figure S6. sVEGFR1 down-regulates VE-Cadherin expression in endothelial cells. (**A**) HUVECs were treated with VEGFA (80 ng/ml) in presence of sVEGFR1 (100 ng/ml) or/and sVEGFR1 neutralizing antibodies (100 ng/ml) for 72 h at 37°C. VE-cadherin protein was analyzed by Western blotting (left). The VE-cadherin protein expression relative to GAPDH was shown (right). n = 3 biological replicates. Statistical significance was determined by two-tailed unpaired *Student t*-test. (****P* = 0.0006, ns = 0.0530)

Data information: Data shown were *Mean* ± *SD* of three biological replicates. (ns, $P > 0.05$; ***, $P < 0.001$)

Appendix Figure S7

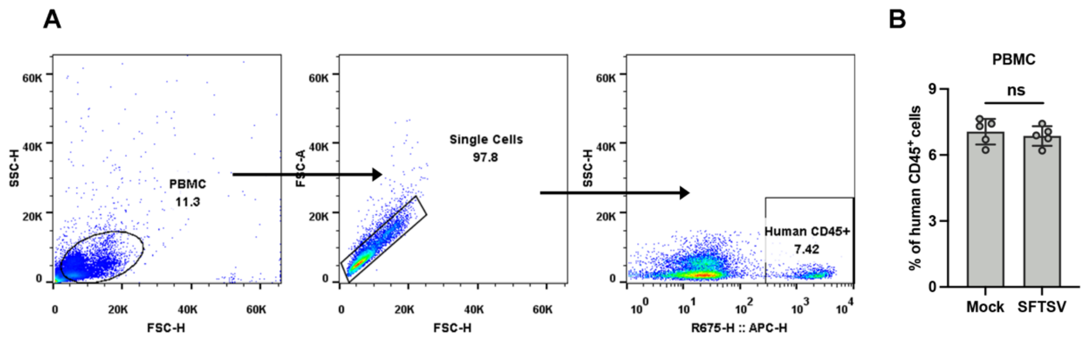

Appendix Figure S7. The levels of engraftment of hu-PBL NCG.

(**A-B**) Flow cytometry analysis of the percentage of human $CD45^{+}$ cells. n = 5 biological replicates.

Statistical significance was determined by two-tailed unpaired *Student t*-test. (ns = 0.5664)

Data information: Data shown were *Mean* ± *SD* of five biological replicates. (ns, $P > 0.05$)

Appendix Table S1 List of 81 genes related to angiogenesis

Gene_name	Mock1	Mock2	Mock3	SFTSV1	SFTSV2	SFTSV3
ADNP	4.21	4.20	4.84	4.07	16.48	19.88
ADAMTS1	14.15	14.87	15.09	17.96	17.50	17.23
AMOTL2	11.78	13.27	15.50	17.50	15.01	18.12
ATM	3.06	3.00	2.79	2.35	2.09	2.10
BASP1	125.21	120.23	21.80	17.56	18.68	20.05
BCL2L1	66.94	62.54	65.48	22.76	20.89	68.64
BCL6	0.16	0.17	0.20	0.31	0.33	0.28
BTN3A2	0.53	0.86	2.23	0.65	0.63	0.60
BCAP31	28.26	47.42	44.89	26.92	26.11	28.00
CHP1	4.22	5.54	15.07	14.34	15.40	14.50
CCN1	55.35	56.61	56.96	75.91	81.99	71.46
CCND3	4.08	3.18	2.82	3.64	6.64	6.55
CCL20	25.98	22.89	23.09	32.56	26.15	33.93
CIAPIN1	14.73	16.79	22.21	23.43	22.68	23.24
CHD9	4.08	4.99	4.74	3.00	3.08	2.99
CXCL2	39.82	39.81	38.41	60.90	54.20	52.05
CXCL8	32.28	29.63	34.66	52.95	44.33	52.00
CELF2	2.81	3.93	3.95	1.44	2.99	1.39
CLDN4	0.51	0.32	0.55	0.93	0.88	0.82
CTDSPL	8.99	2.80	3.12	2.38	2.47	2.54
CXCL3	3.02	2.61	2.26	3.82	3.71	4.91
CLK1	9.03	8.06	8.49	6.03	5.93	7.04
CBFB	7.32	9.05	8.31	8.53	20.54	8.80
CREB1	1.14	1.22	1.15	1.39	3.11	1.17
CSNK2B	48.21	16.43	49.35	19.70	14.89	16.42
CXCL1	317.85	300.11	295.96	369.98	359.39	371.67
DUSP3	24.84	25.62	26.36	26.32	3.48	3.85
DEGS1	16.50	24.25	14.95	23.80	22.87	28.09
EGR1	10.82	11.39	9.64	22.10	22.11	18.44
F11R	2.28	2.50	3.49	12.53	2.97	2.22
FOS	0.29	0.19	0.28	0.38	0.76	0.40
FKRP	2.10	0.35	1.85	0.35	0.57	0.29
HMGA2	8.42	5.45	8.99	5.18	4.47	5.95
HAS3	21.06	21.08	11.69	22.32	18.02	52.82
ICAM3	3.49	3.44	2.66	2.73	2.04	1.28
IL6	48.90	61.47	59.22	87.59	65.05	66.27
IFIT2	0.72	0.65	0.81	2.19	2.11	2.11
JUNB	14.39	13.88	13.57	19.69	17.50	16.81

LITAF	22.21	21.44	21.30	13.17	20.53	9.72
LY6E	12.29	14.56	22.44	13.64	11.26	10.25
LASP1	50.82	12.71	50.45	12.62	14.38	16.40
MKLN1	5.18	5.69	5.45	11.14	10.44	5.28
NF2	10.90	14.59	17.52	9.72	10.21	10.01
NONO	63.88	67.75	66.71	66.18	84.50	109.90
ORAI1	5.23	12.39	5.95	12.23	11.46	11.89
PTPN12	14.86	13.78	16.97	19.95	20.83	16.21
POLG2	2.90	2.95	2.91	1.68	1.49	1.70
PRCP	2.90	2.90	3.34	4.98	5.16	5.46
PPT1	37.57	29.62	18.64	37.67	35.33	40.22
PDXP	2.49	2.00	13.45	13.36	13.02	13.72
PDLIM5	2.35	4.39	5.16	2.42	2.46	2.48
PAQR3	2.42	2.27	2.38	1.38	2.07	0.84
PTX3	23.96	21.34	19.47	27.21	25.12	28.49
RBMS3	0.17	0.35	0.30	0.13	0.13	0.17
RNF146	5.08	5.22	4.92	3.11	3.54	4.30
RBM15	0.65	2.76	3.52	0.69	0.67	0.65
RAB5B	2.60	21.58	3.00	3.19	2.59	2.88
RNF114	22.76	23.18	23.08	21.99	10.25	10.81
RHOB	13.57	12.57	13.85	18.96	17.72	17.49
RCAN3	1.90	1.78	2.18	2.96	2.78	3.01
RRM2	14.30	15.64	14.01	14.65	29.90	25.49
RPL29	145.90	132.71	116.51	155.50	261.49	143.36
RO60	1.08	1.51	1.93	2.70	2.28	3.05
SLFN5	1.55	1.24	1.35	3.11	1.32	2.98
SOCS7	0.39	2.89	0.59	0.43	0.46	0.51
SLC12A6	1.33	1.39	1.42	1.27	0.64	0.63
S1PR3	0.03	0.03	0.04	0.08	0.04	0.07
SNX1	8.79	7.45	5.49	5.25	5.55	5.58
SUOX	3.26	2.16	2.30	6.35	6.47	4.44
TSKU	5.00	4.62	5.37	1.43	2.86	2.30
TNF	36.74	33.73	34.32	56.43	50.33	54.34
TUBA1A	15.59	12.33	13.45	41.25	39.93	19.16
TGFB2	0.33	0.37	0.12	0.56	0.40	0.52
TMCO1	14.39	13.01	12.16	10.45	9.04	11.43
TRIM47	14.15	12.79	13.42	7.58	8.07	7.17
WNT2B	0.48	0.24	0.24	0.11	0.19	0.12
WNT5B	4.18	9.25	4.18	3.52	3.78	4.06
WEE1	4.00	2.23	4.05	1.94	1.80	1.89
ZNF24	2.12	13.74	3.07	13.70	13.60	13.65

ZNF281	6.69	5.33	5.48	16.85	16.30	16.41
XBP1	16.86	10.07	17.74	9.98	10.77	11.17
