# Supplementary figures and images for: sVEGFR1 up-regulation via EGR1 impairs vascular repair in SFTSV-induced hemorrhage
Source: EMBO Rep. 2025 Aug 11;26(18):4477–502. doi: 10.1038/s44319-025-00541-2 (PMC12457690; doi:10.1038/s44319-025-00541-2)

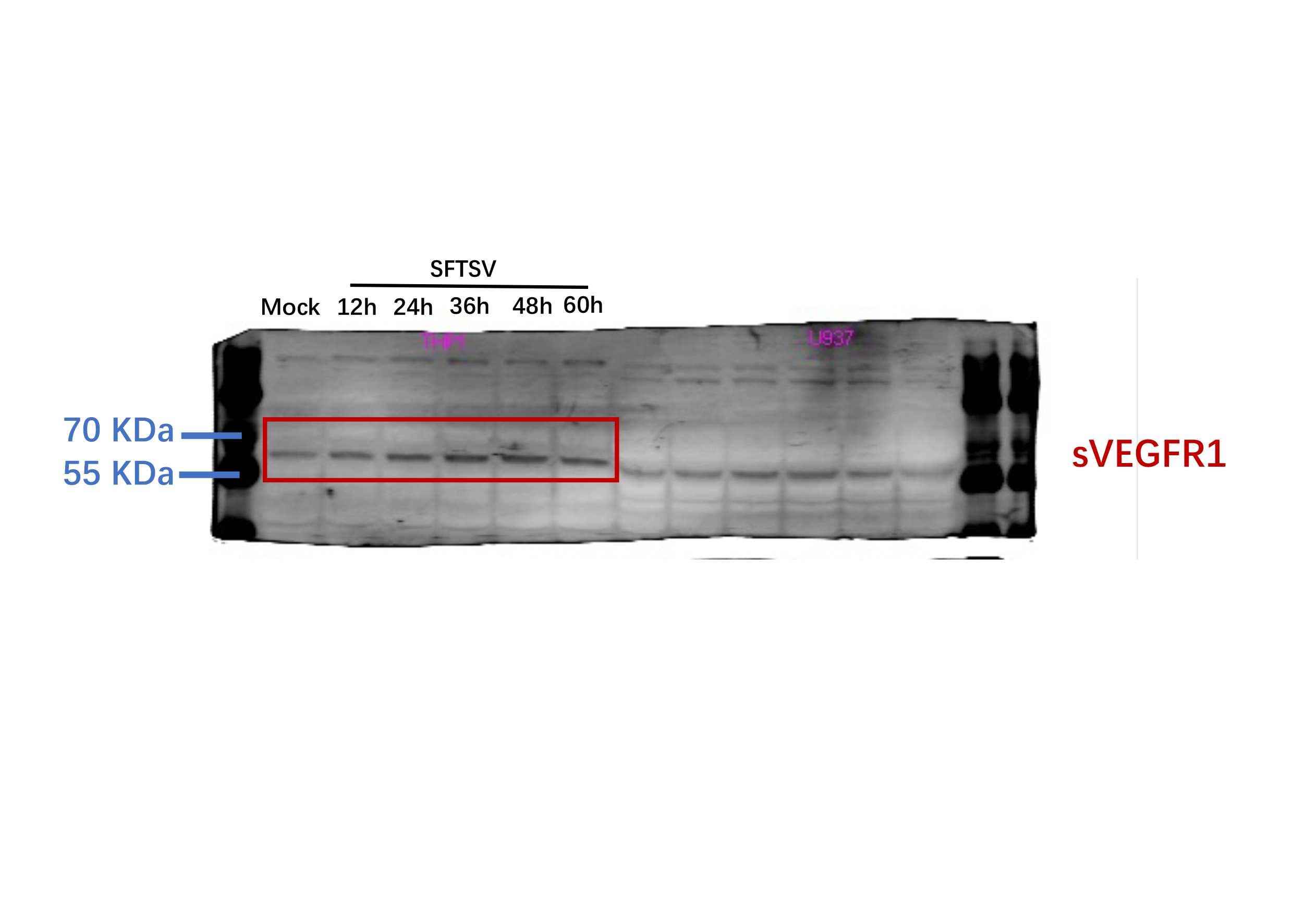

Supplement: Supplementary file 3 — Source data Fig. 1 [file 44319_2025_541_MOESM3_ESM.zip › Figure 1/1B/western sVEGFR1.tiff]

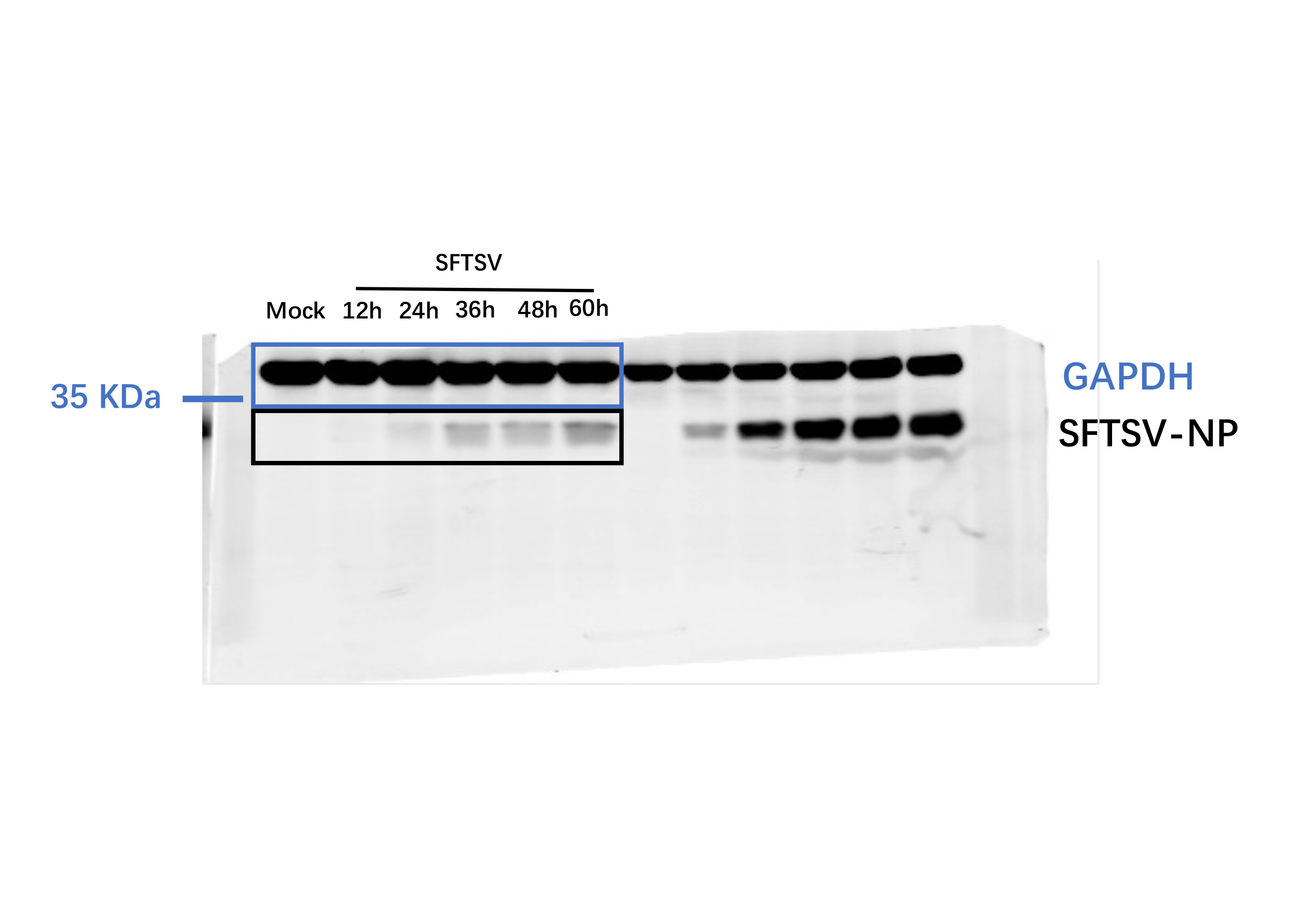

Supplement: Supplementary file 3 — Source data Fig. 1 [file 44319_2025_541_MOESM3_ESM.zip › Figure 1/1B/western NP and GAPDH.tiff]

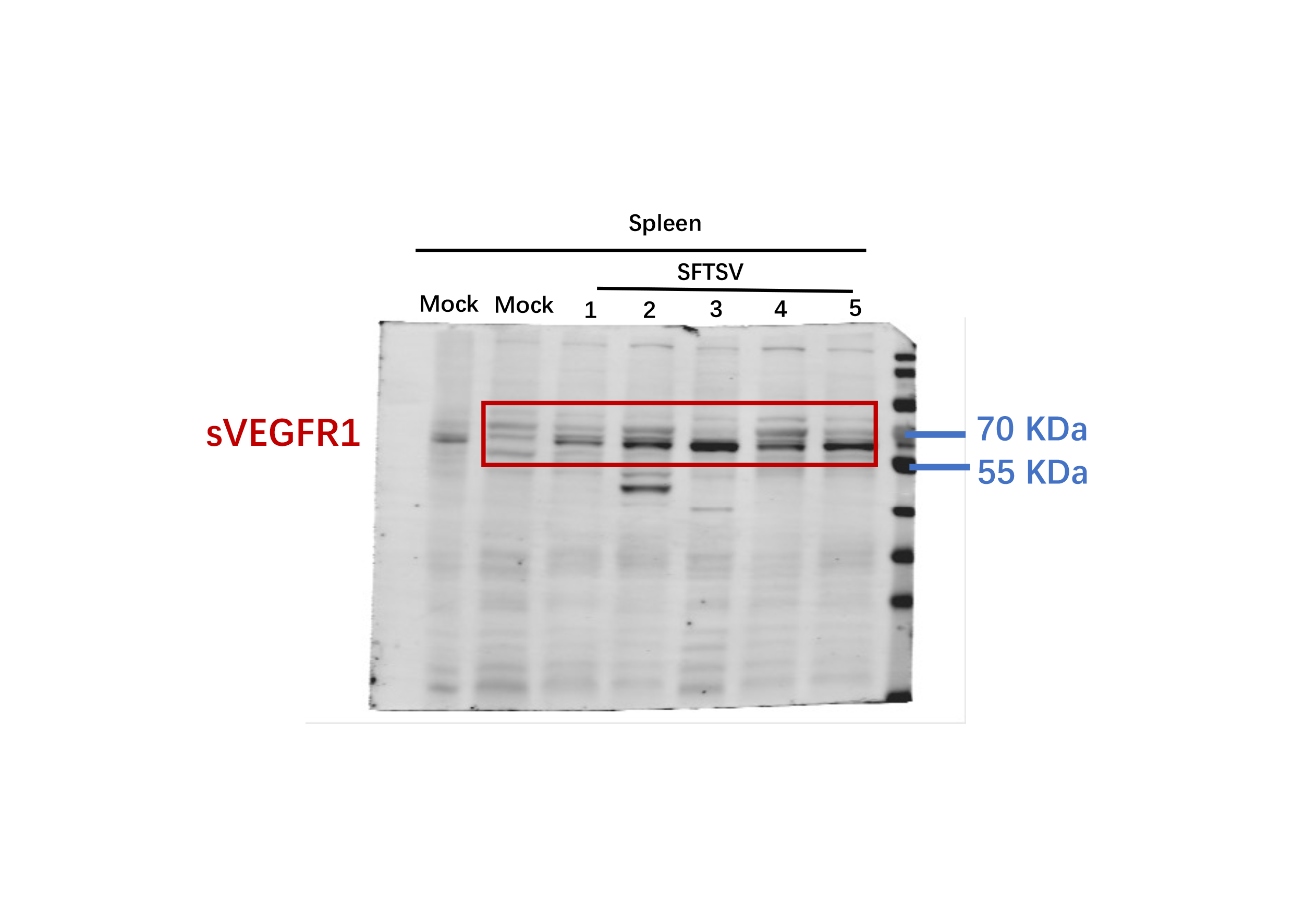

Supplement: Supplementary file 4 — Source data Fig. 2 [file 44319_2025_541_MOESM4_ESM.zip › Figure 2/2C/western sVEGFR1.tiff]

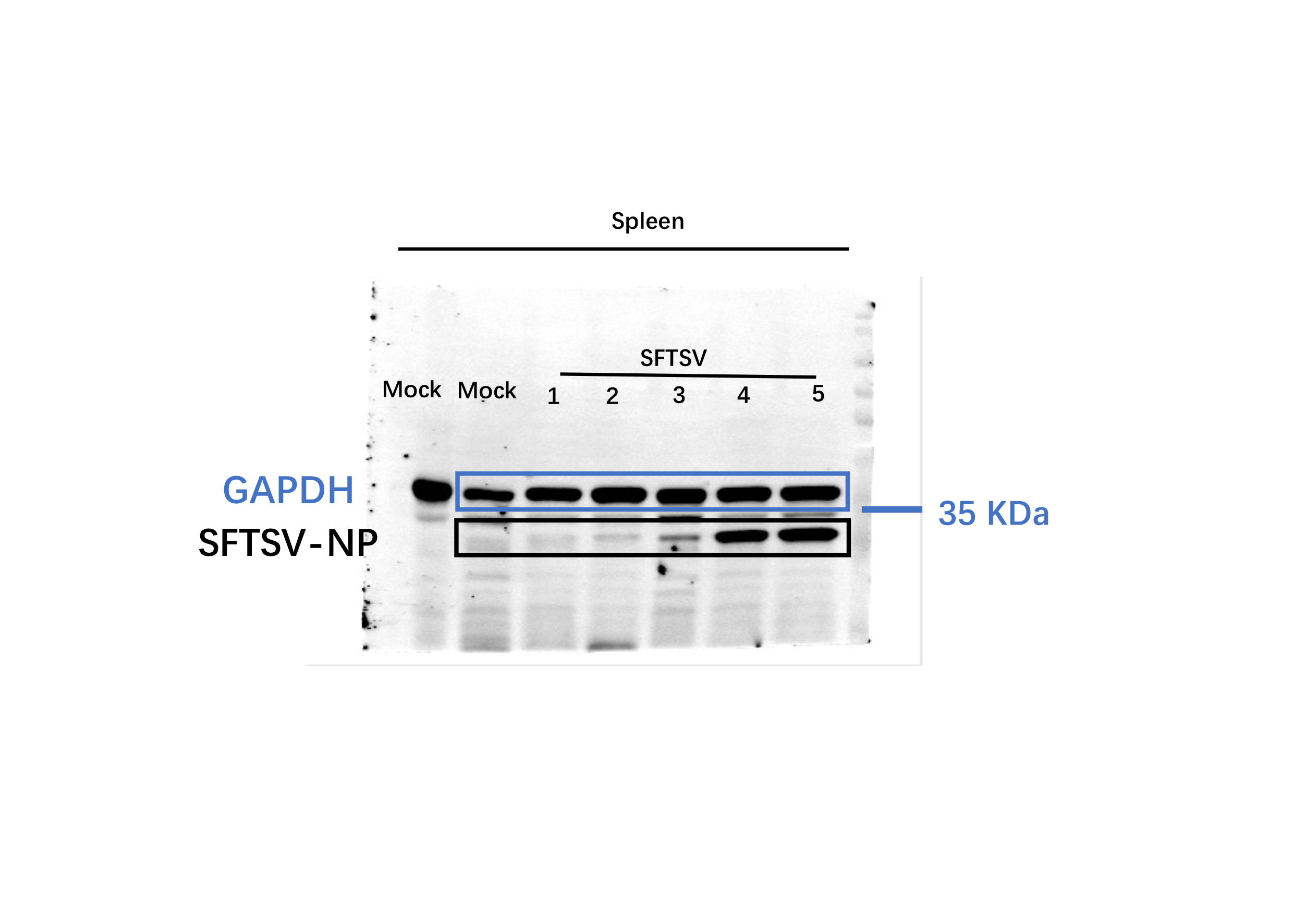

Supplement: Supplementary file 4 — Source data Fig. 2 [file 44319_2025_541_MOESM4_ESM.zip › Figure 2/2C/western NP and GAPDH.tiff]

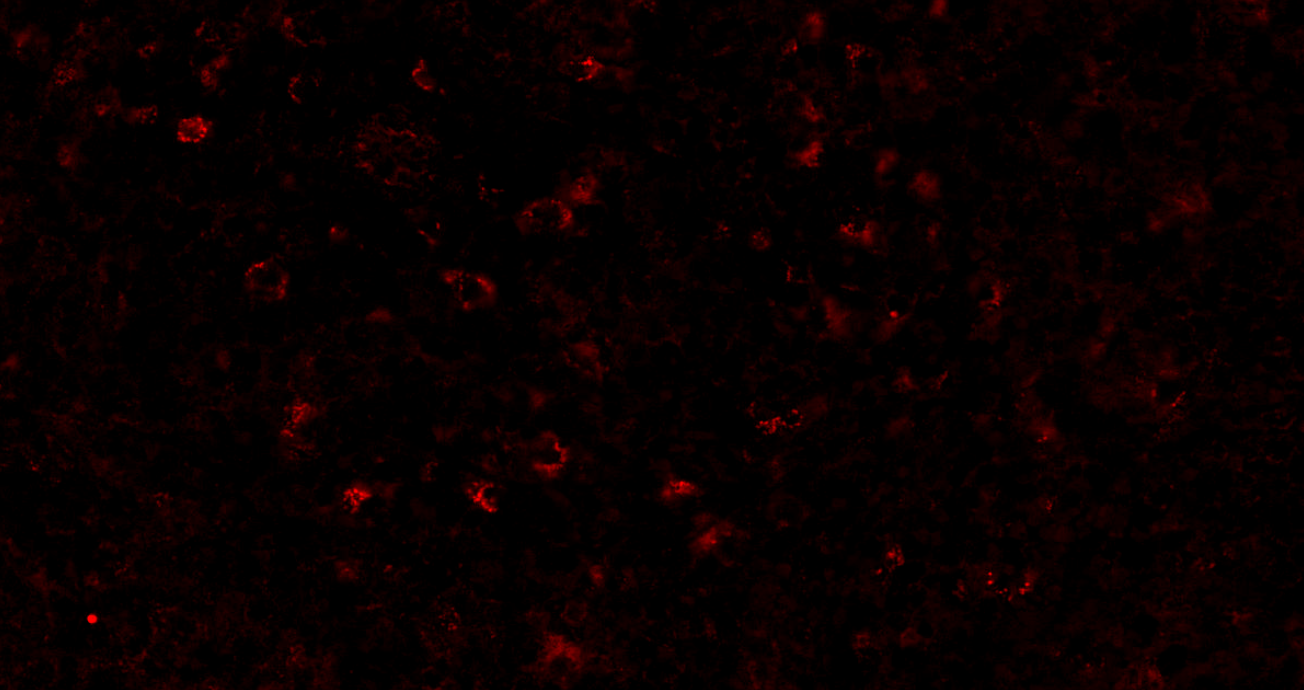

Supplement: Supplementary file 4 — Source data Fig. 2 [file 44319_2025_541_MOESM4_ESM.zip › Figure 2/2D/SFTSV_spleen SFTSV-Gn.tiff]

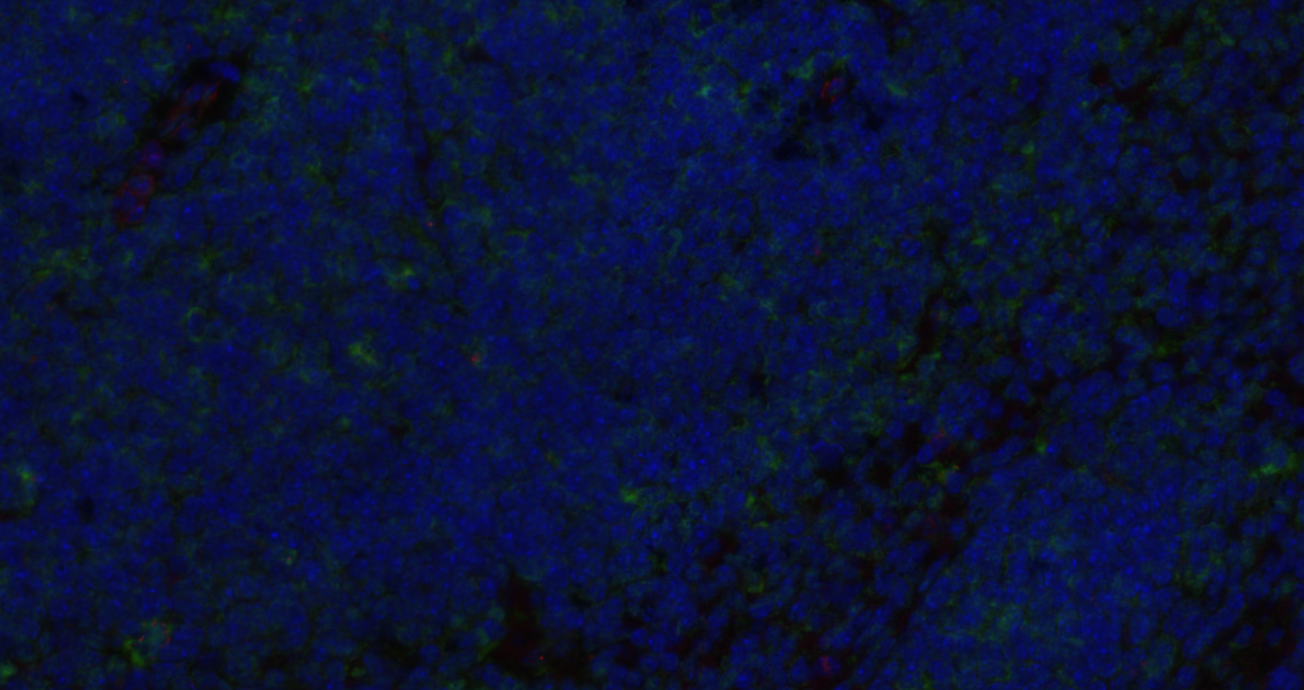

Supplement: Supplementary file 4 — Source data Fig. 2 [file 44319_2025_541_MOESM4_ESM.zip › Figure 2/2D/Mock_spleen merge.tiff]

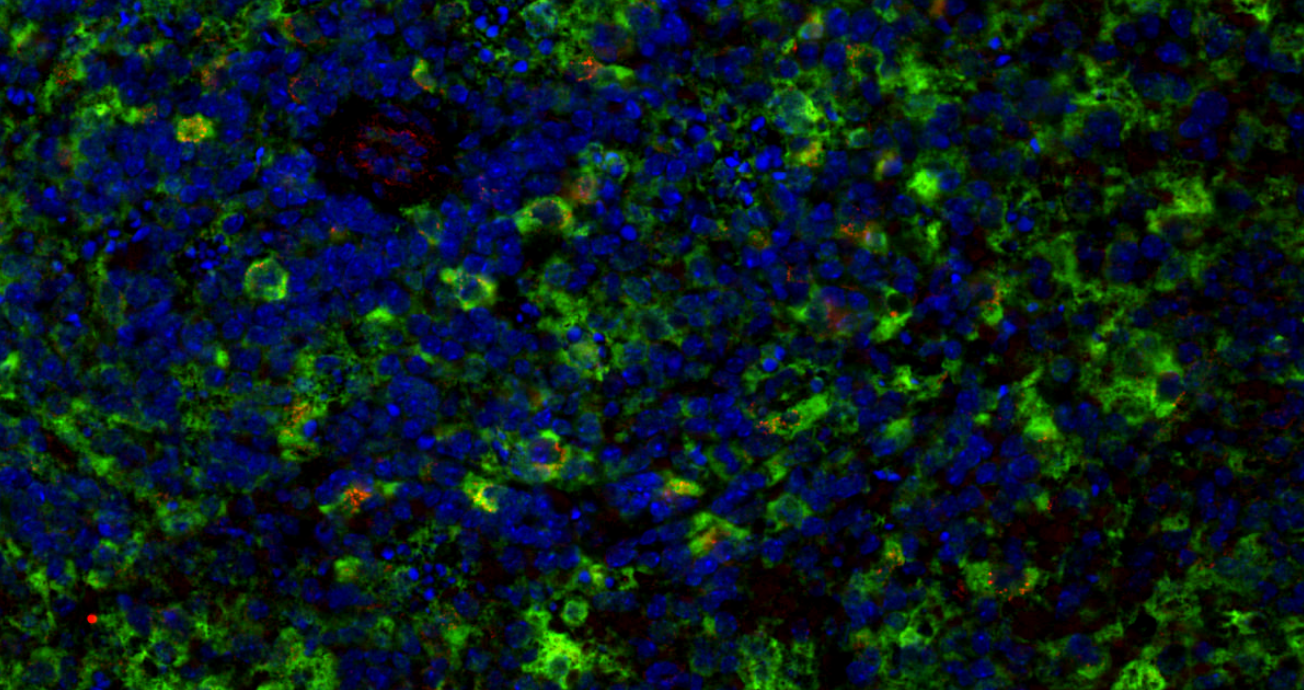

Supplement: Supplementary file 4 — Source data Fig. 2 [file 44319_2025_541_MOESM4_ESM.zip › Figure 2/2D/SFTSV_spleen merge.tiff]

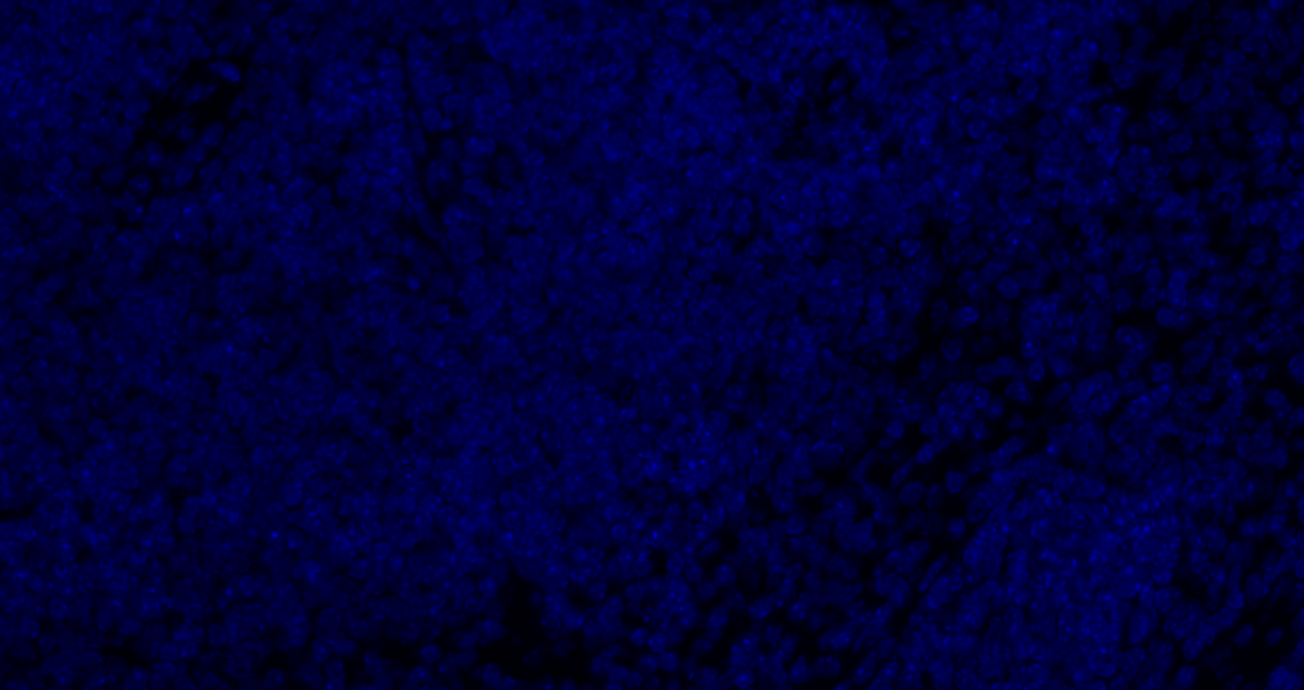

Supplement: Supplementary file 4 — Source data Fig. 2 [file 44319_2025_541_MOESM4_ESM.zip › Figure 2/2D/Mock_spleen dapi.tiff]

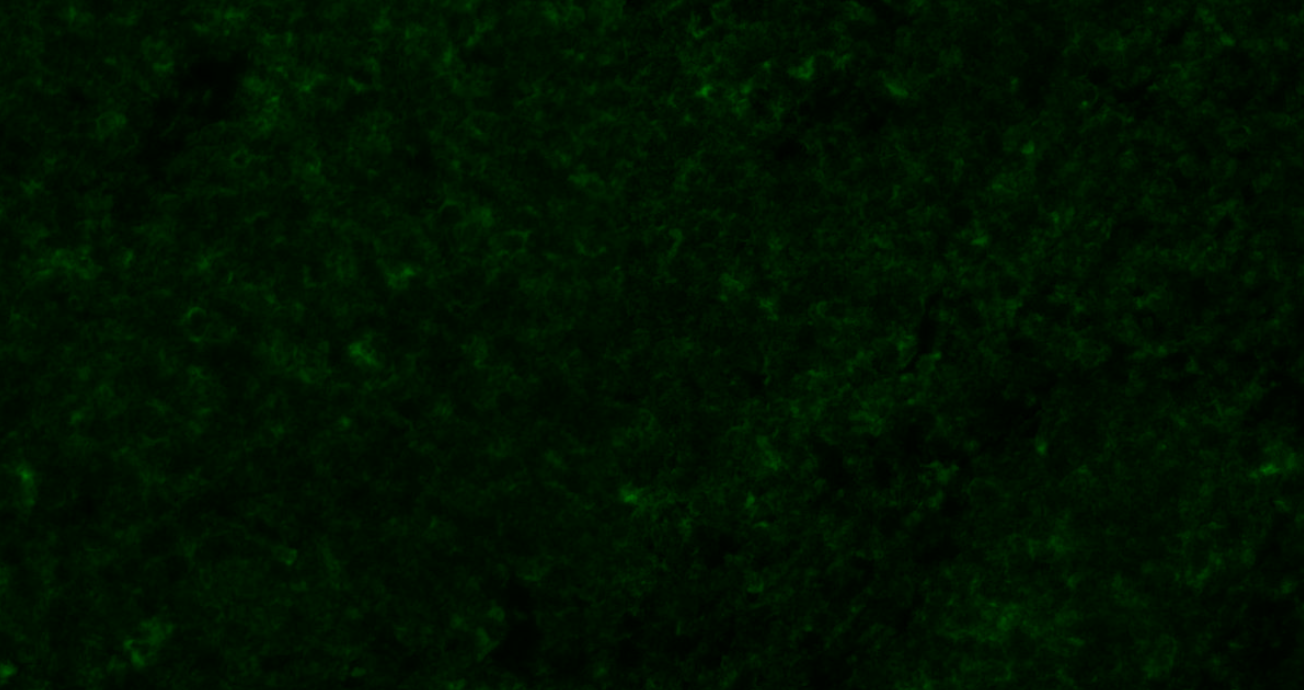

Supplement: Supplementary file 4 — Source data Fig. 2 [file 44319_2025_541_MOESM4_ESM.zip › Figure 2/2D/Mock_spleen sVEGFR1.tiff]

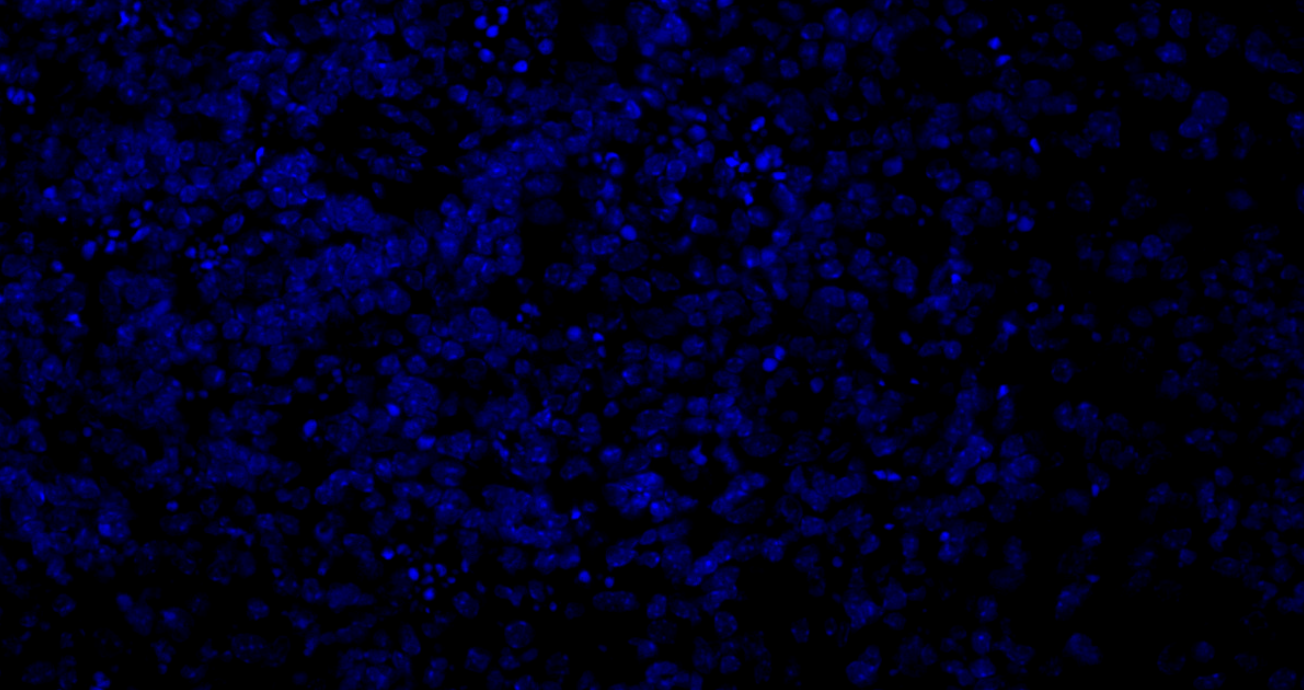

Supplement: Supplementary file 4 — Source data Fig. 2 [file 44319_2025_541_MOESM4_ESM.zip › Figure 2/2D/SFTSV_spleen dapi.tiff]

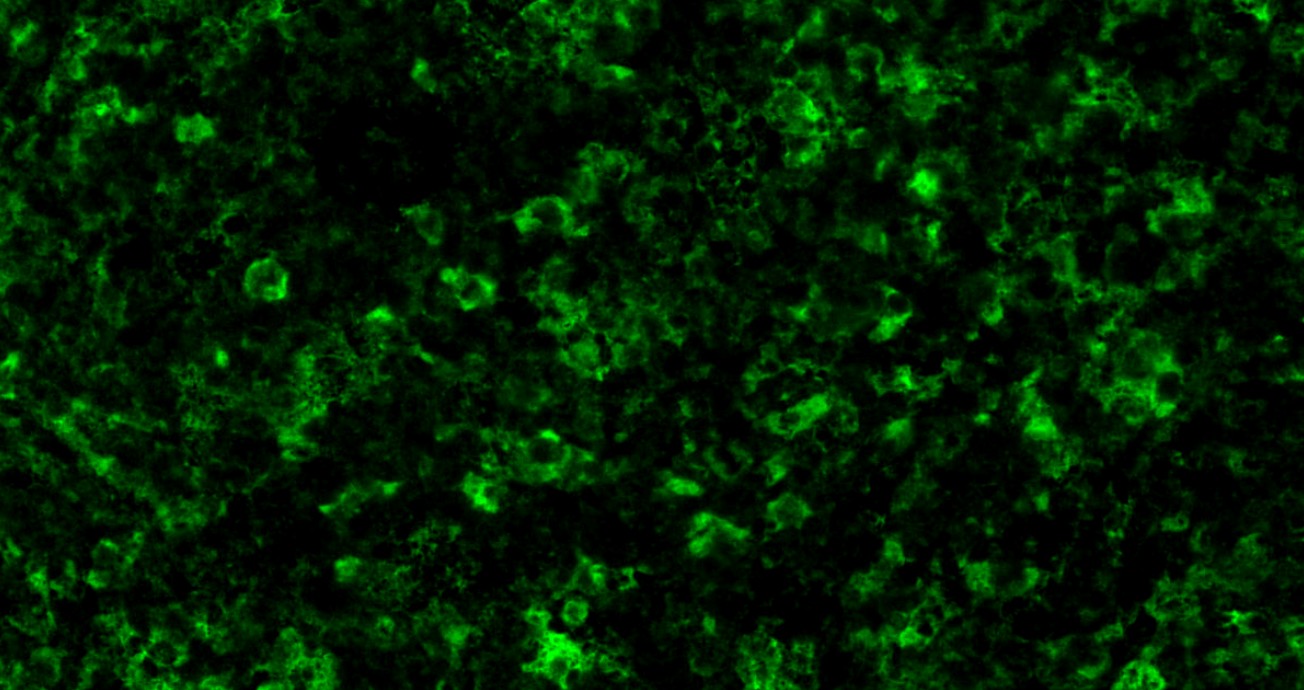

Supplement: Supplementary file 4 — Source data Fig. 2 [file 44319_2025_541_MOESM4_ESM.zip › Figure 2/2D/SFTSV_spleen sVEGFR1.tiff]

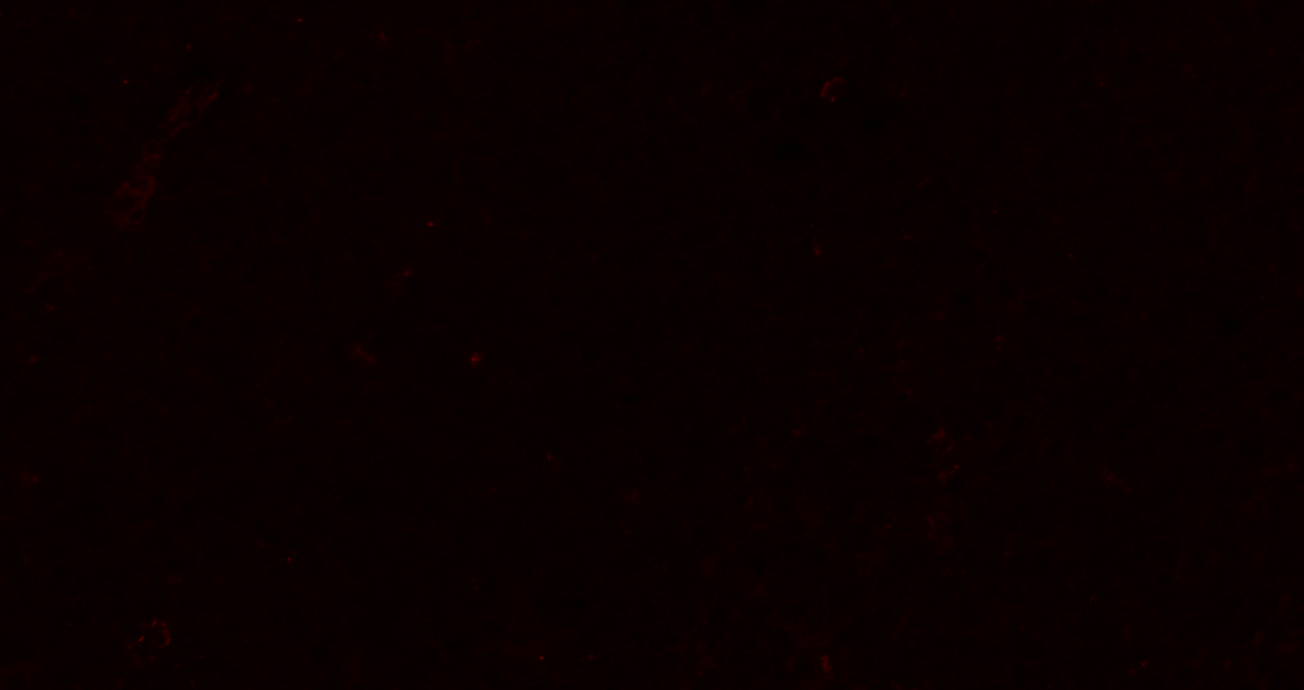

Supplement: Supplementary file 4 — Source data Fig. 2 [file 44319_2025_541_MOESM4_ESM.zip › Figure 2/2D/Mock_spleen SFTSV-Gn.tiff]

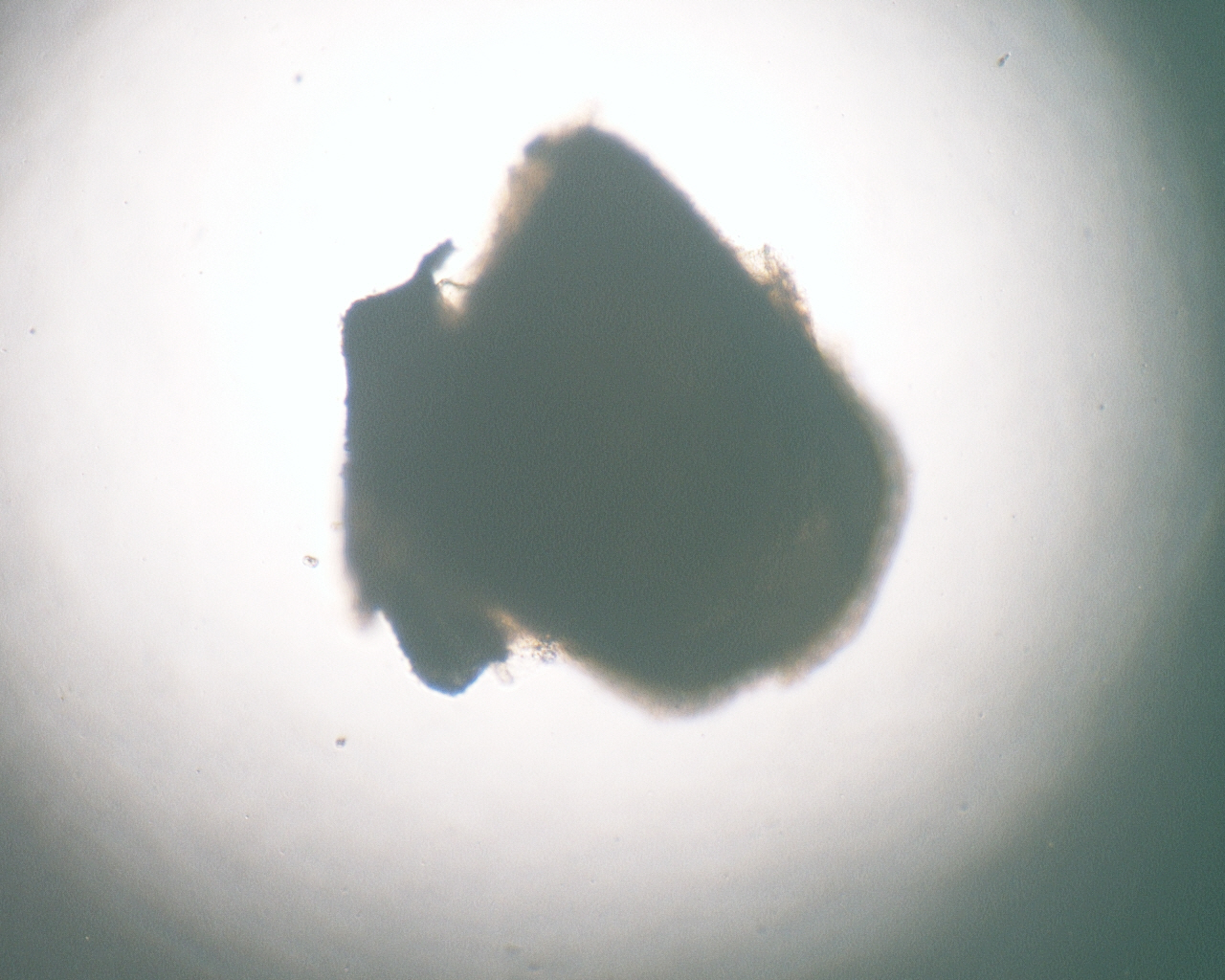

Supplement: Supplementary file 5 — Source data Fig. 3 [file 44319_2025_541_MOESM5_ESM.zip › Figure 3/3B/severe group Day5 4X.tiff]

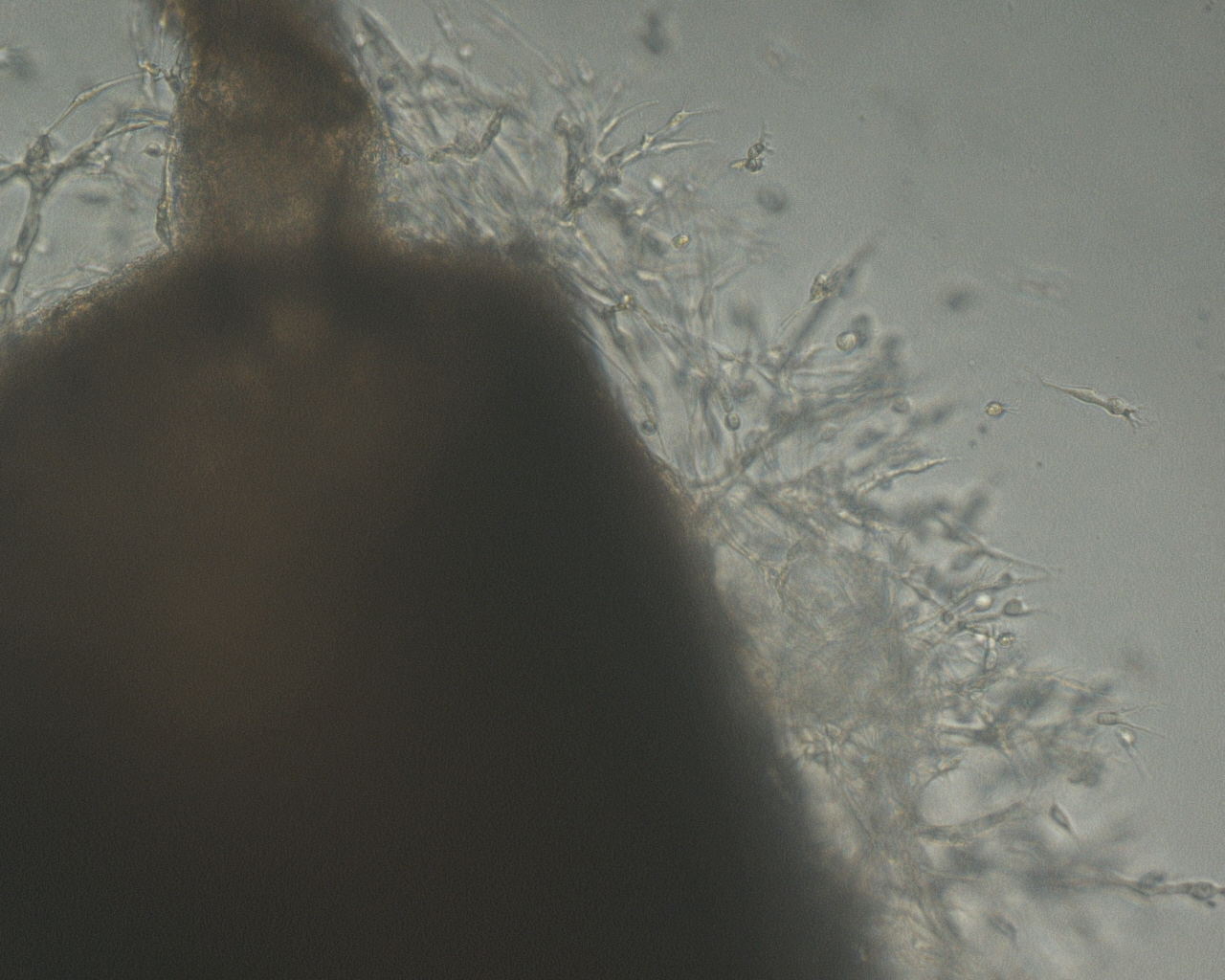

Supplement: Supplementary file 5 — Source data Fig. 3 [file 44319_2025_541_MOESM5_ESM.zip › Figure 3/3B/control group Day5 10X.tiff]

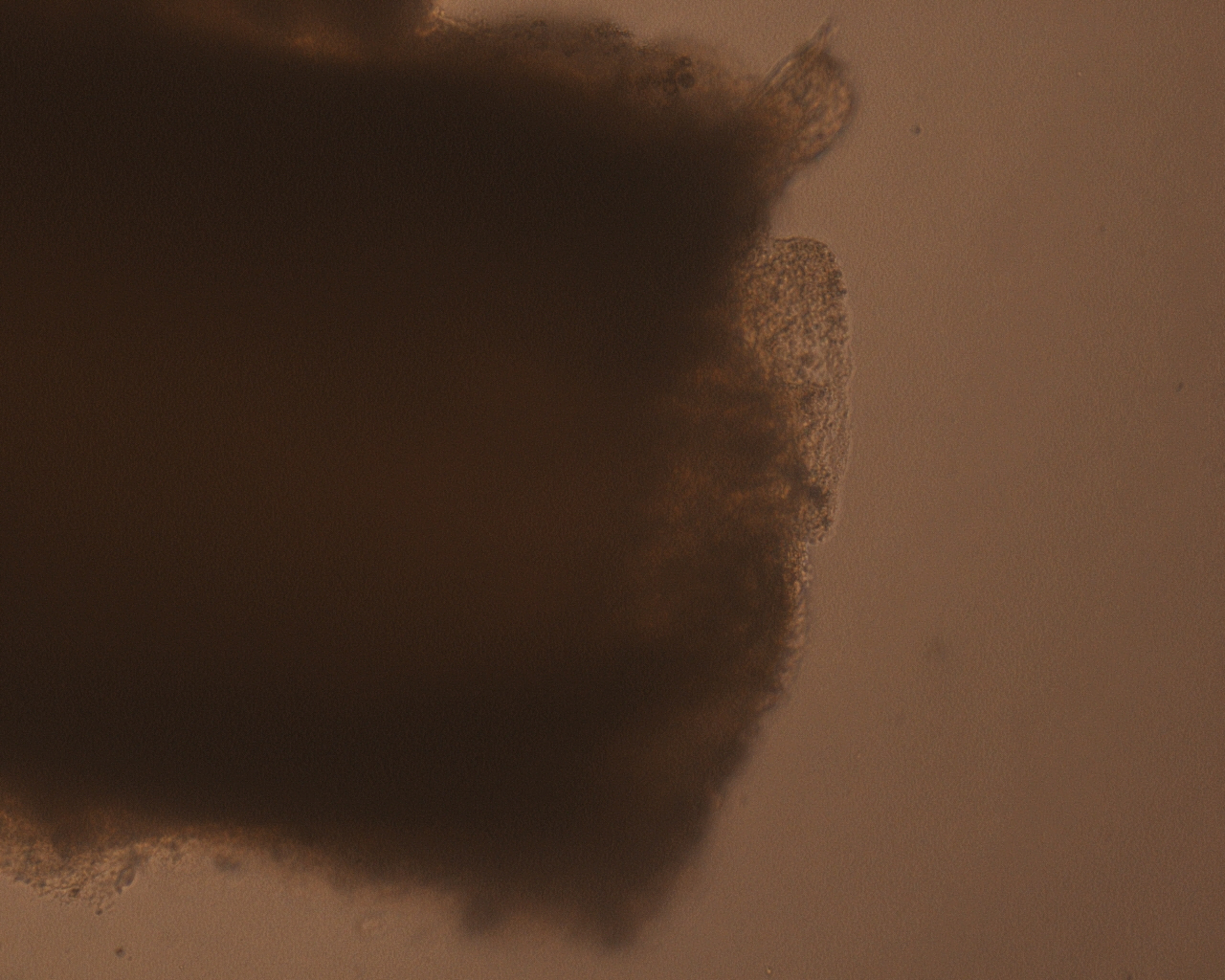

Supplement: Supplementary file 5 — Source data Fig. 3 [file 44319_2025_541_MOESM5_ESM.zip › Figure 3/3B/mild group Day3 10X.tiff]

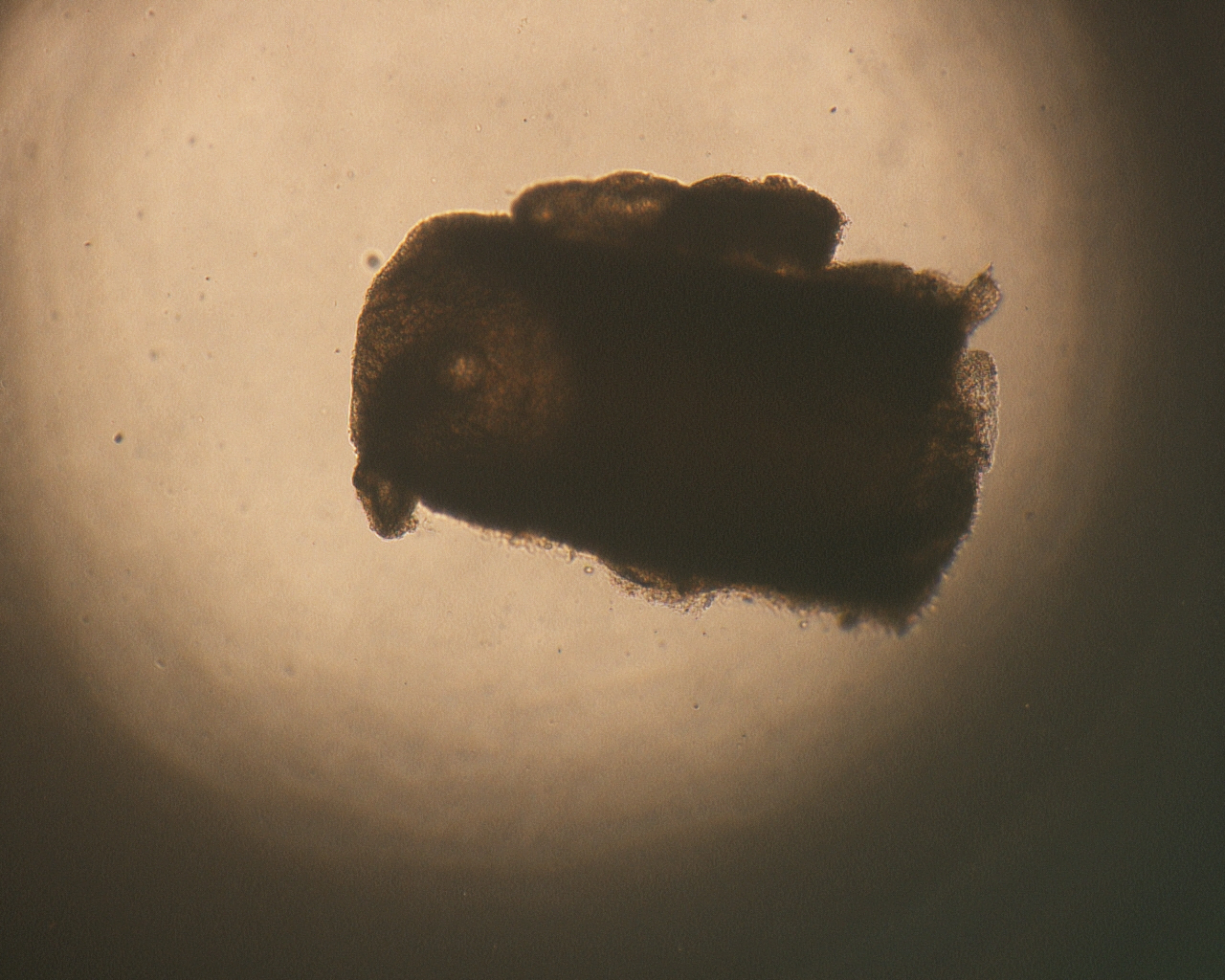

Supplement: Supplementary file 5 — Source data Fig. 3 [file 44319_2025_541_MOESM5_ESM.zip › Figure 3/3B/mild group Day3 4X.tiff]

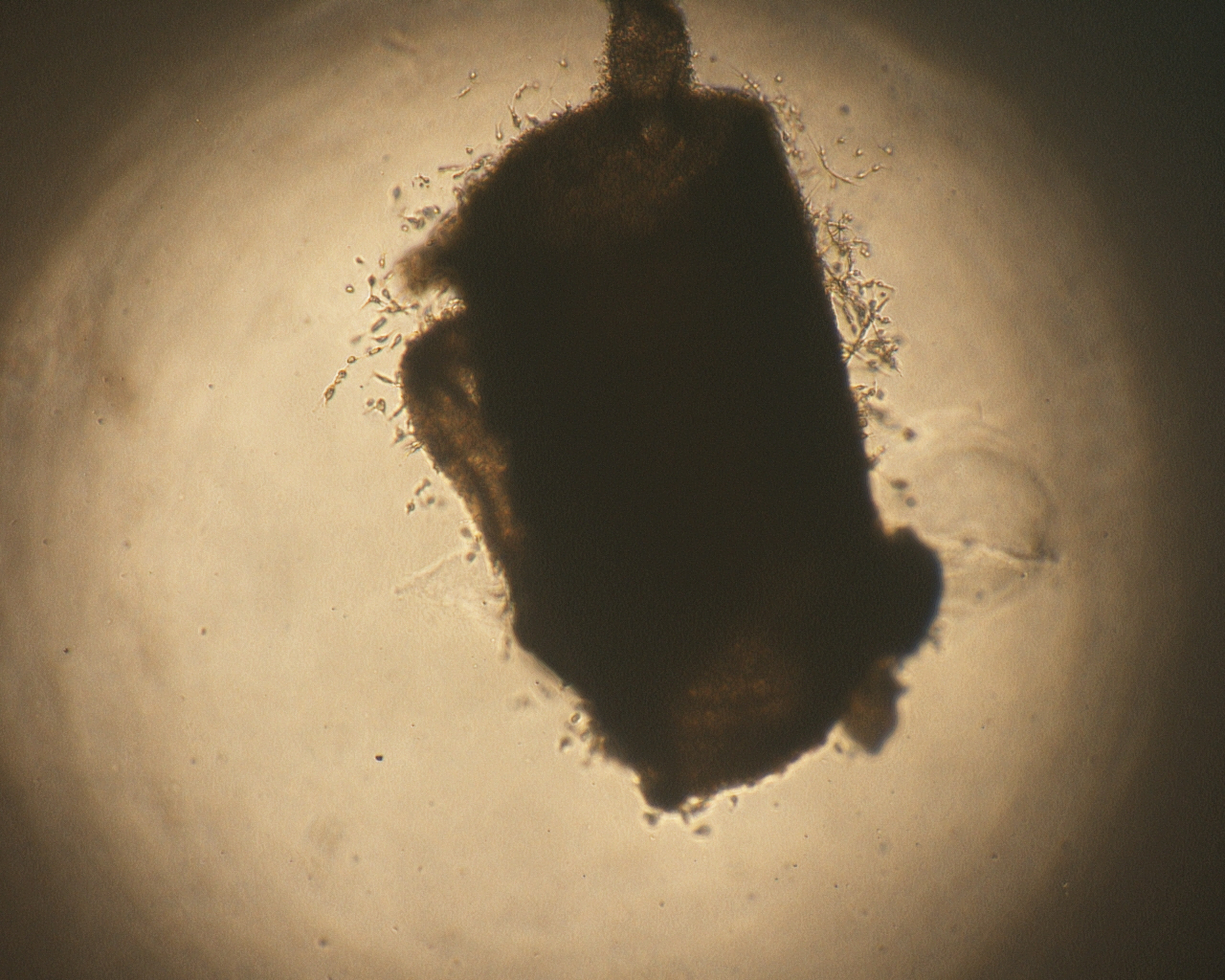

Supplement: Supplementary file 5 — Source data Fig. 3 [file 44319_2025_541_MOESM5_ESM.zip › Figure 3/3B/control group Day3 4X.tiff]

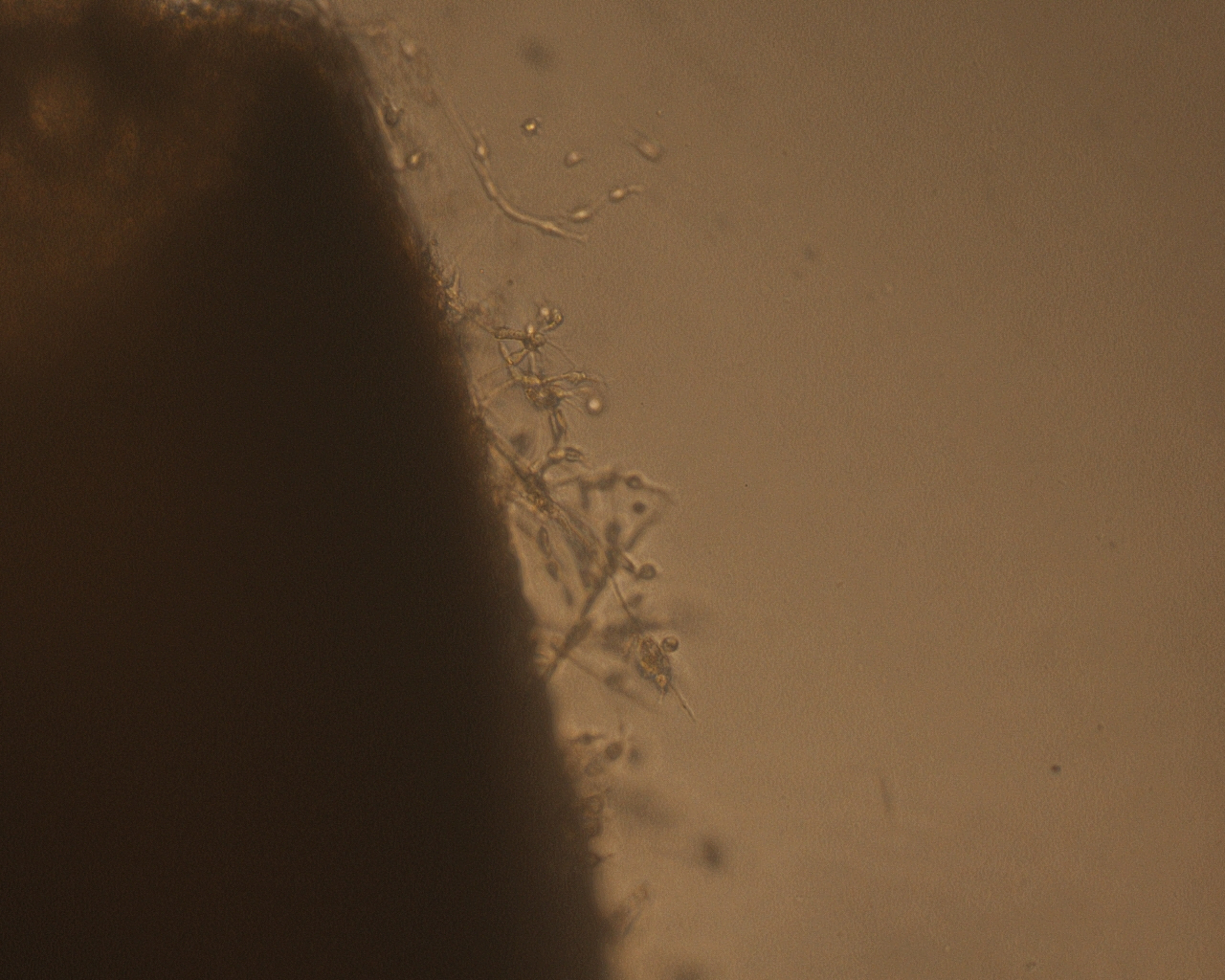

Supplement: Supplementary file 5 — Source data Fig. 3 [file 44319_2025_541_MOESM5_ESM.zip › Figure 3/3B/control group Day3 10X.tiff]

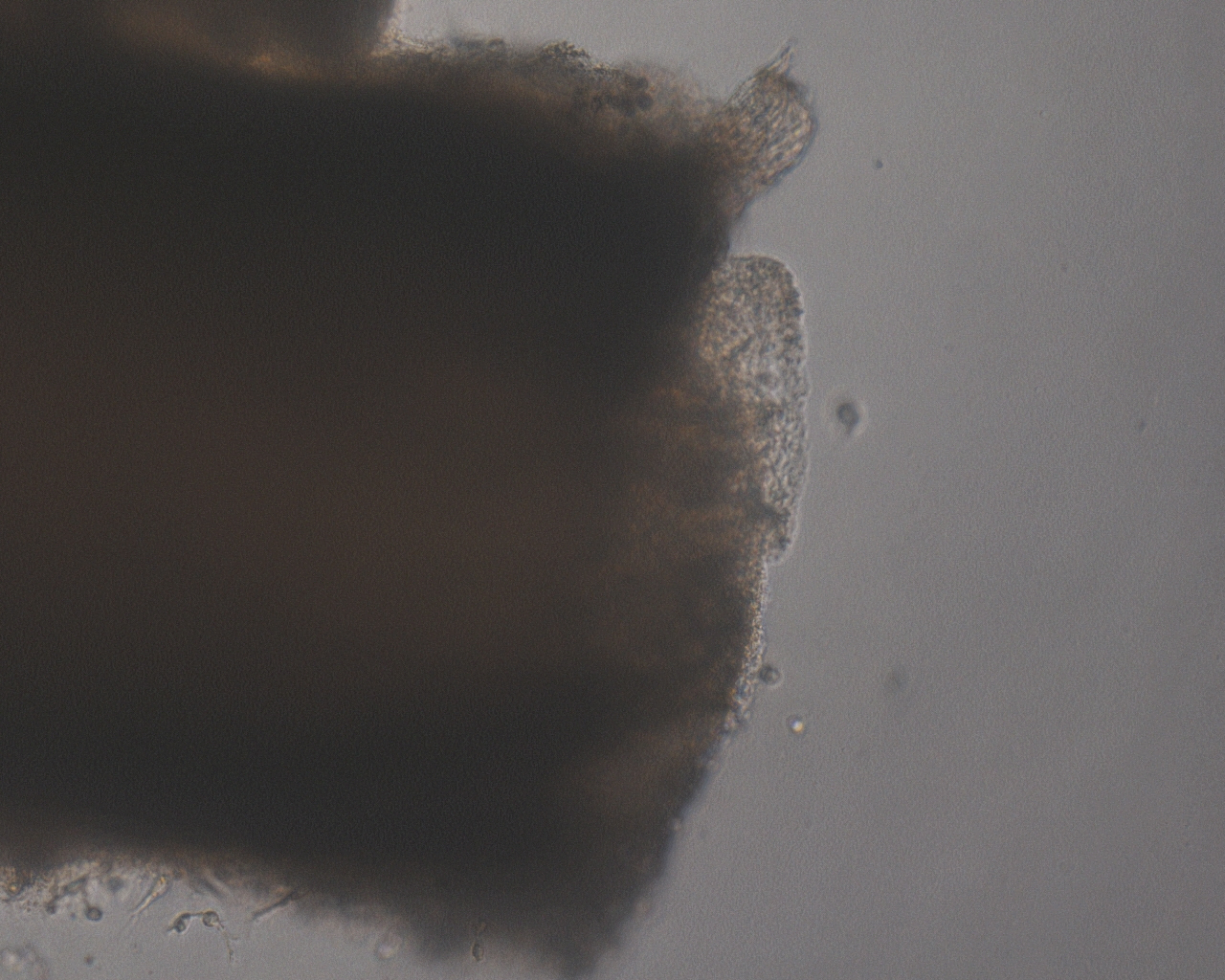

Supplement: Supplementary file 5 — Source data Fig. 3 [file 44319_2025_541_MOESM5_ESM.zip › Figure 3/3B/mild group Day5 10X.tiff]

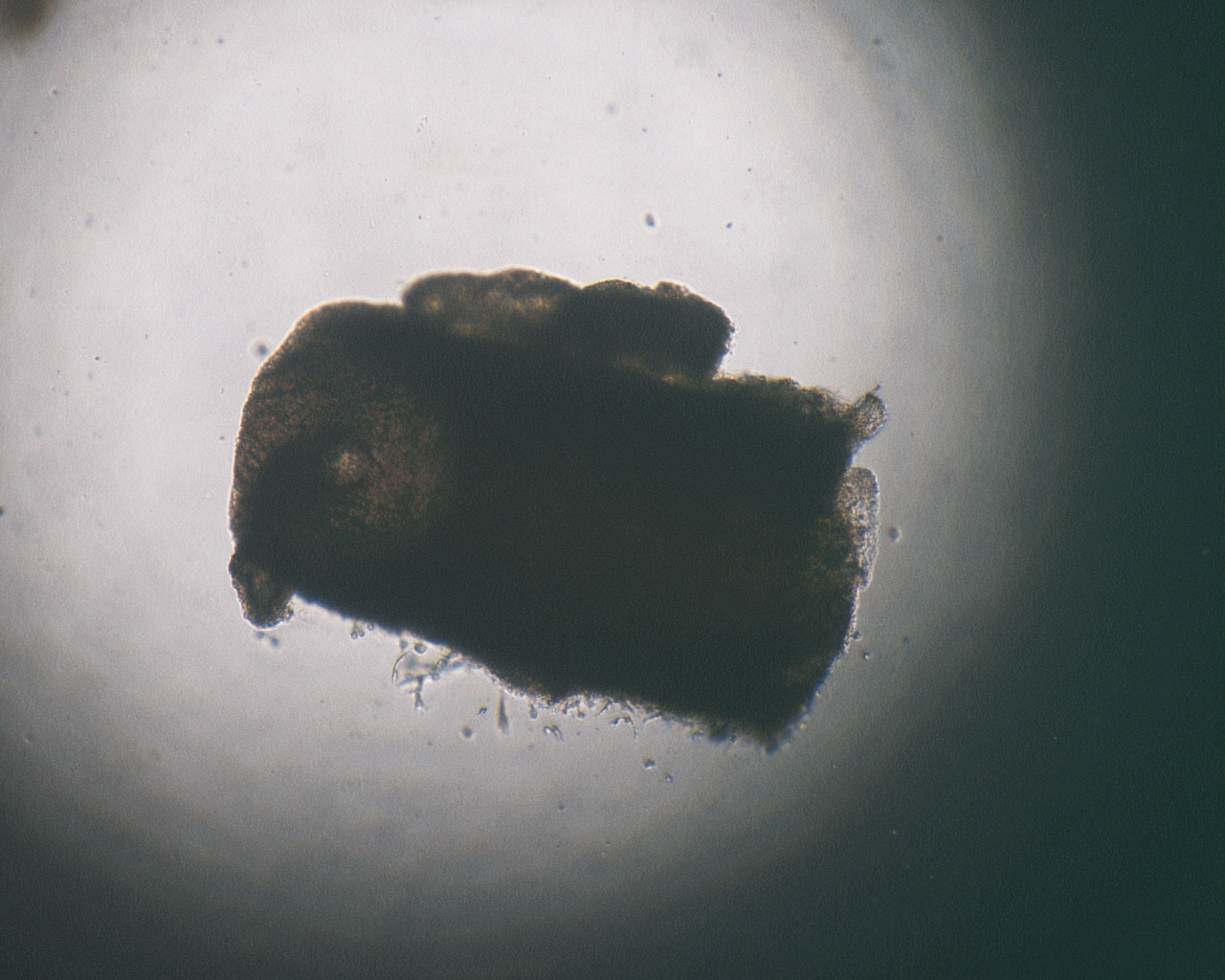

Supplement: Supplementary file 5 — Source data Fig. 3 [file 44319_2025_541_MOESM5_ESM.zip › Figure 3/3B/mild group Day5 4X.tiff]

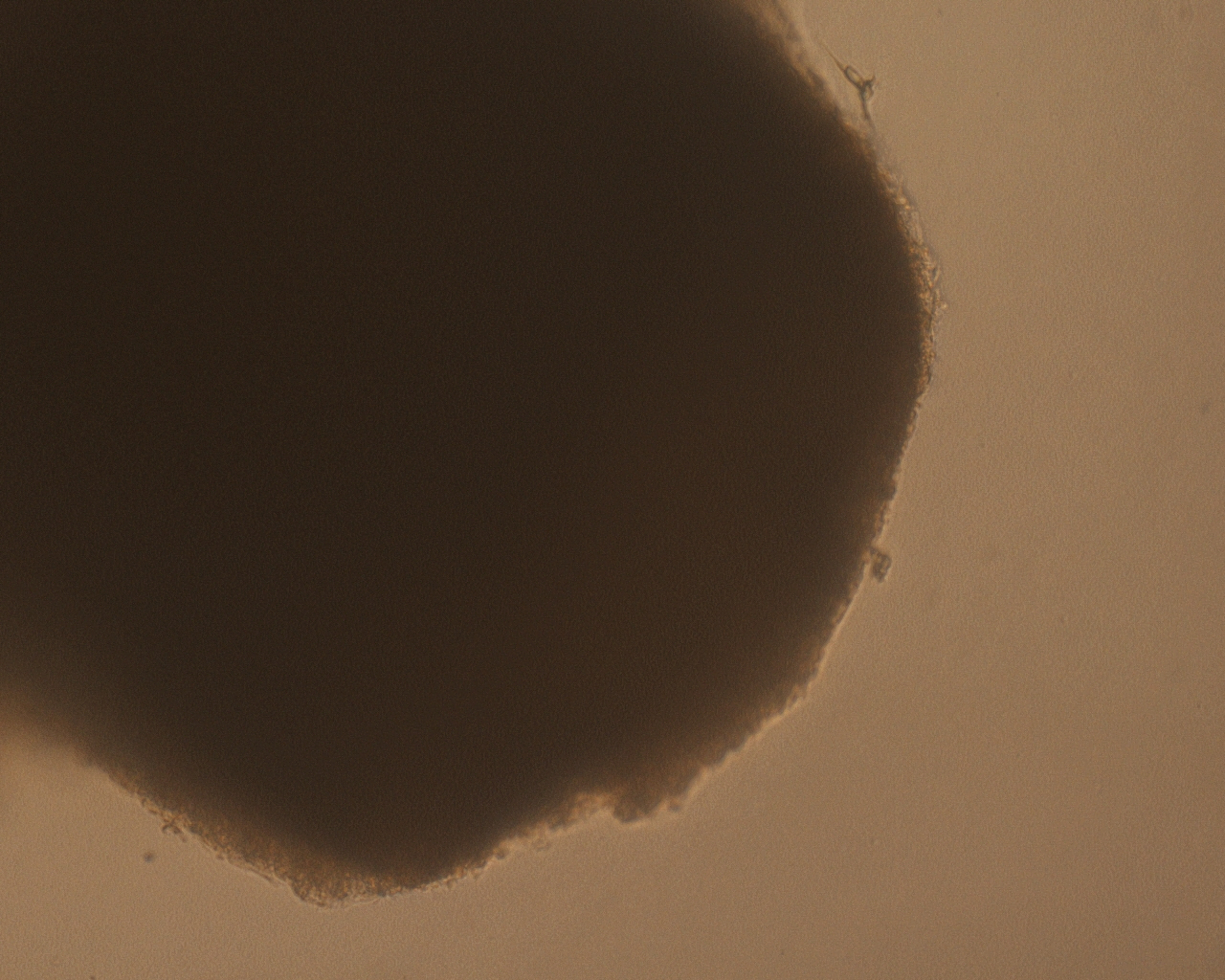

Supplement: Supplementary file 5 — Source data Fig. 3 [file 44319_2025_541_MOESM5_ESM.zip › Figure 3/3B/severe group Day3 10X.tiff]

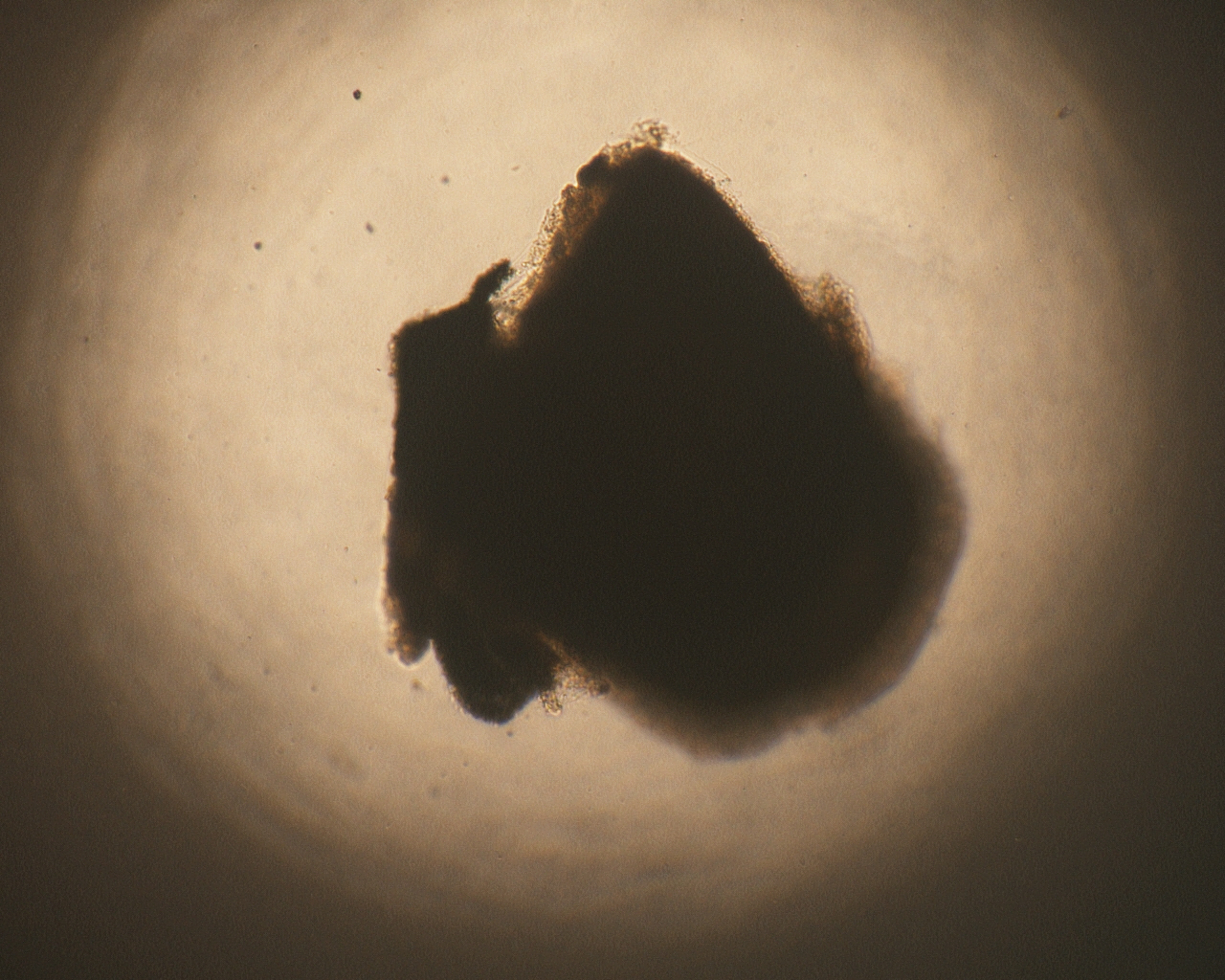

Supplement: Supplementary file 5 — Source data Fig. 3 [file 44319_2025_541_MOESM5_ESM.zip › Figure 3/3B/severe group Day3 4X.tiff]

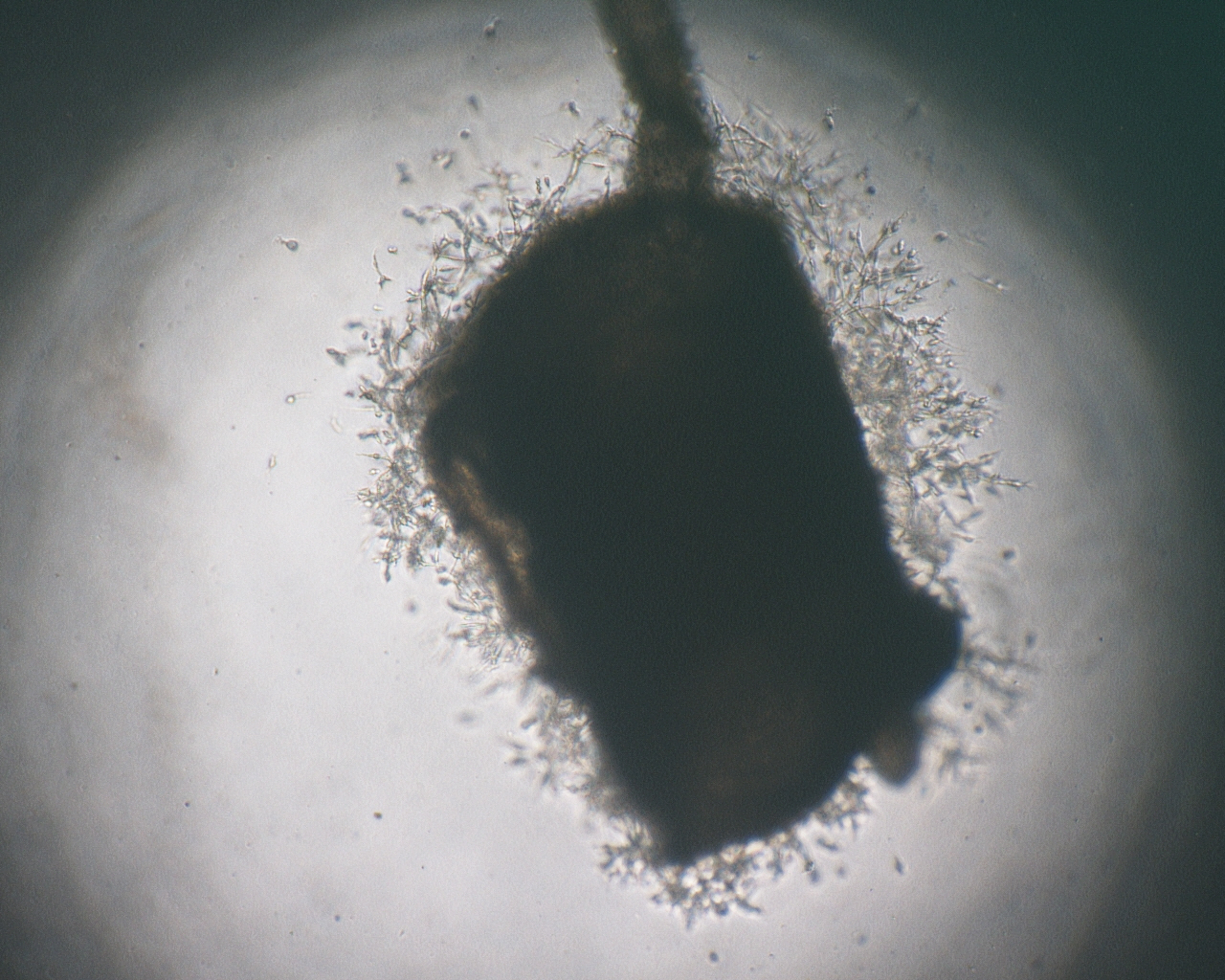

Supplement: Supplementary file 5 — Source data Fig. 3 [file 44319_2025_541_MOESM5_ESM.zip › Figure 3/3B/control group Day5 4X.tiff]

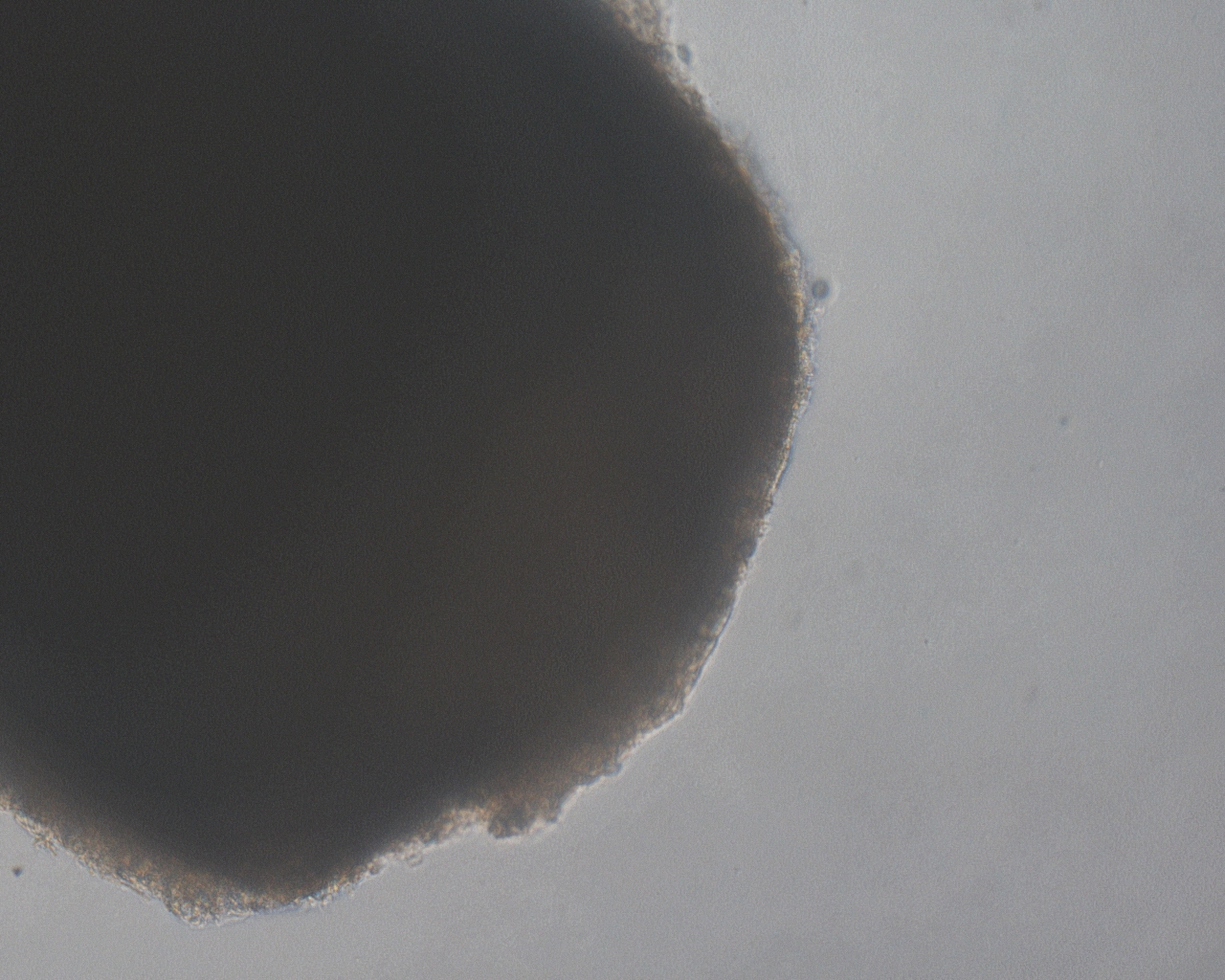

Supplement: Supplementary file 5 — Source data Fig. 3 [file 44319_2025_541_MOESM5_ESM.zip › Figure 3/3B/severe group Day5 10X.tiff]

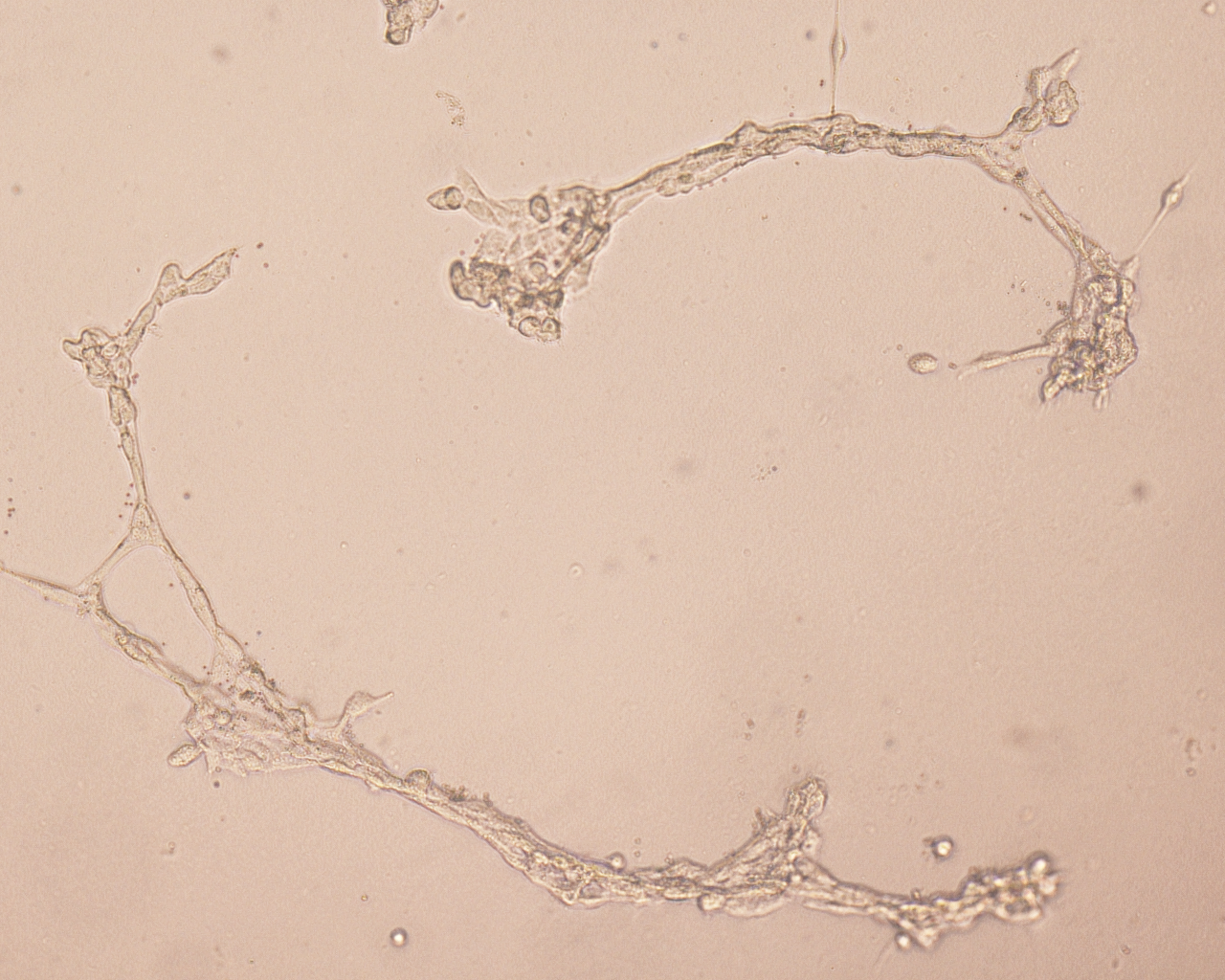

Supplement: Supplementary file 5 — Source data Fig. 3 [file 44319_2025_541_MOESM5_ESM.zip › Figure 3/3C/SFTS group anti-sVEGFR1.tiff]

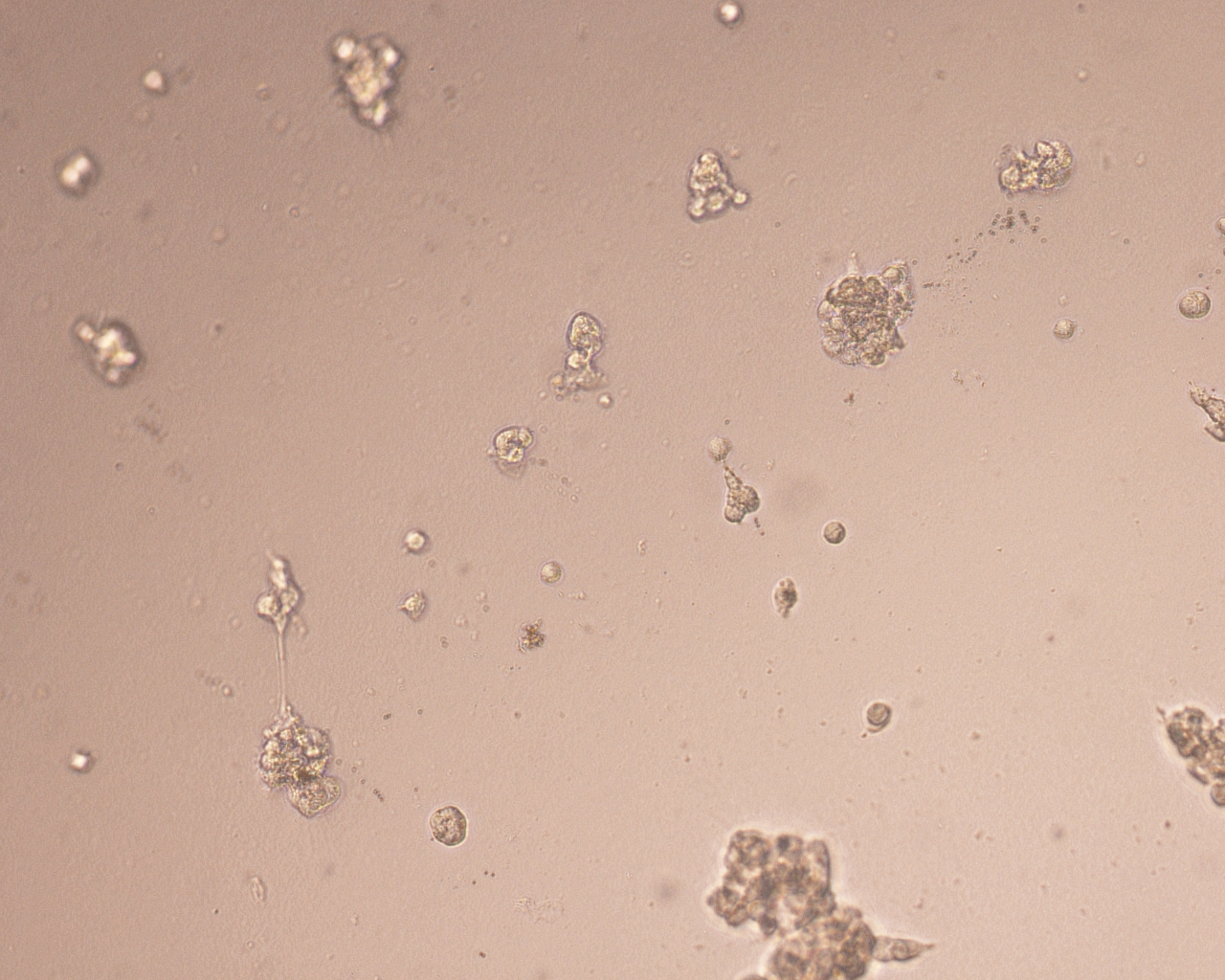

Supplement: Supplementary file 5 — Source data Fig. 3 [file 44319_2025_541_MOESM5_ESM.zip › Figure 3/3C/SFTS group anti-Con.tiff]

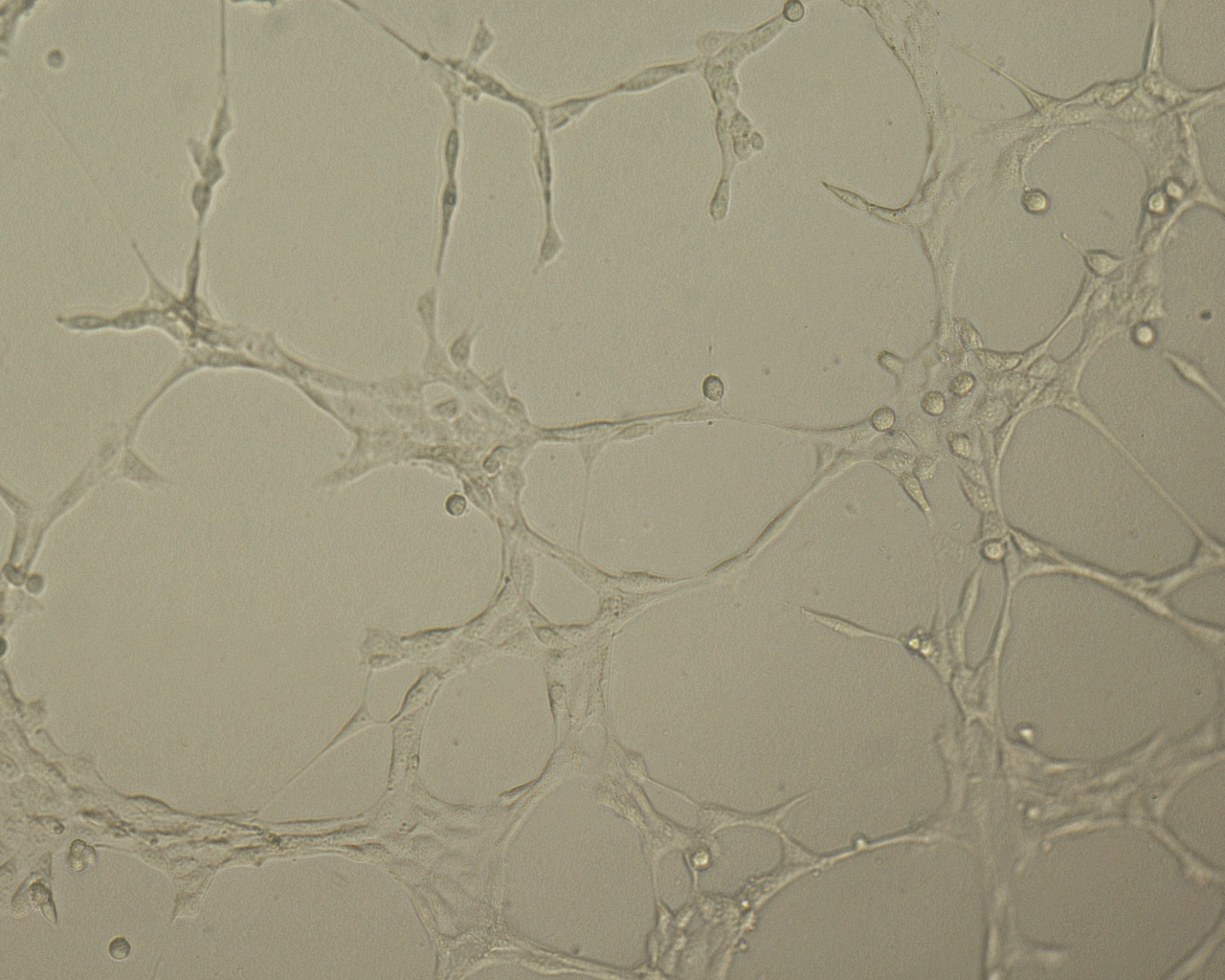

Supplement: Supplementary file 5 — Source data Fig. 3 [file 44319_2025_541_MOESM5_ESM.zip › Figure 3/3C/control group anti-sVEGFR1.tiff]

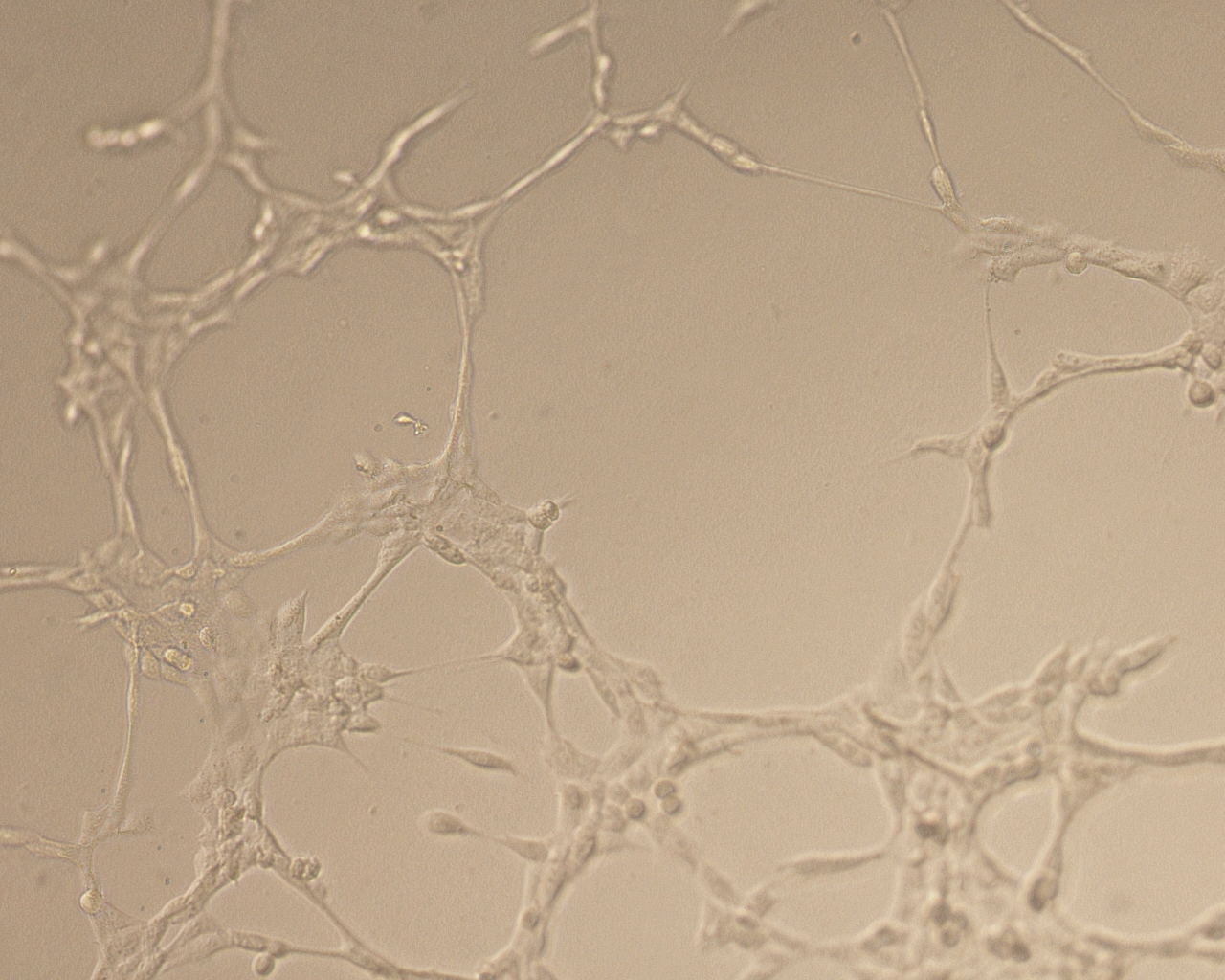

Supplement: Supplementary file 5 — Source data Fig. 3 [file 44319_2025_541_MOESM5_ESM.zip › Figure 3/3C/control group anti-Con.tiff]

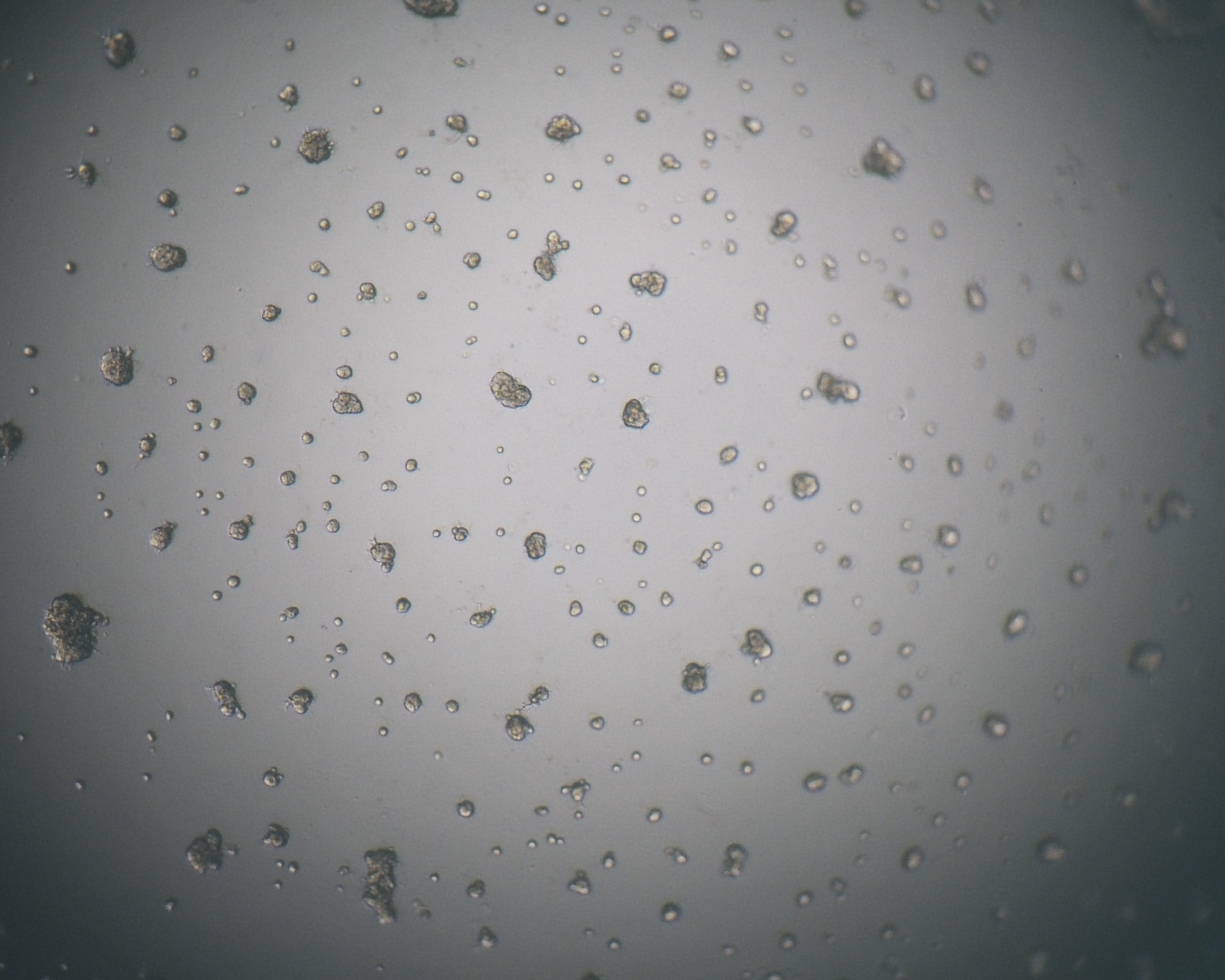

Supplement: Supplementary file 5 — Source data Fig. 3 [file 44319_2025_541_MOESM5_ESM.zip › Figure 3/3D/SFTS group none 24h.tiff]

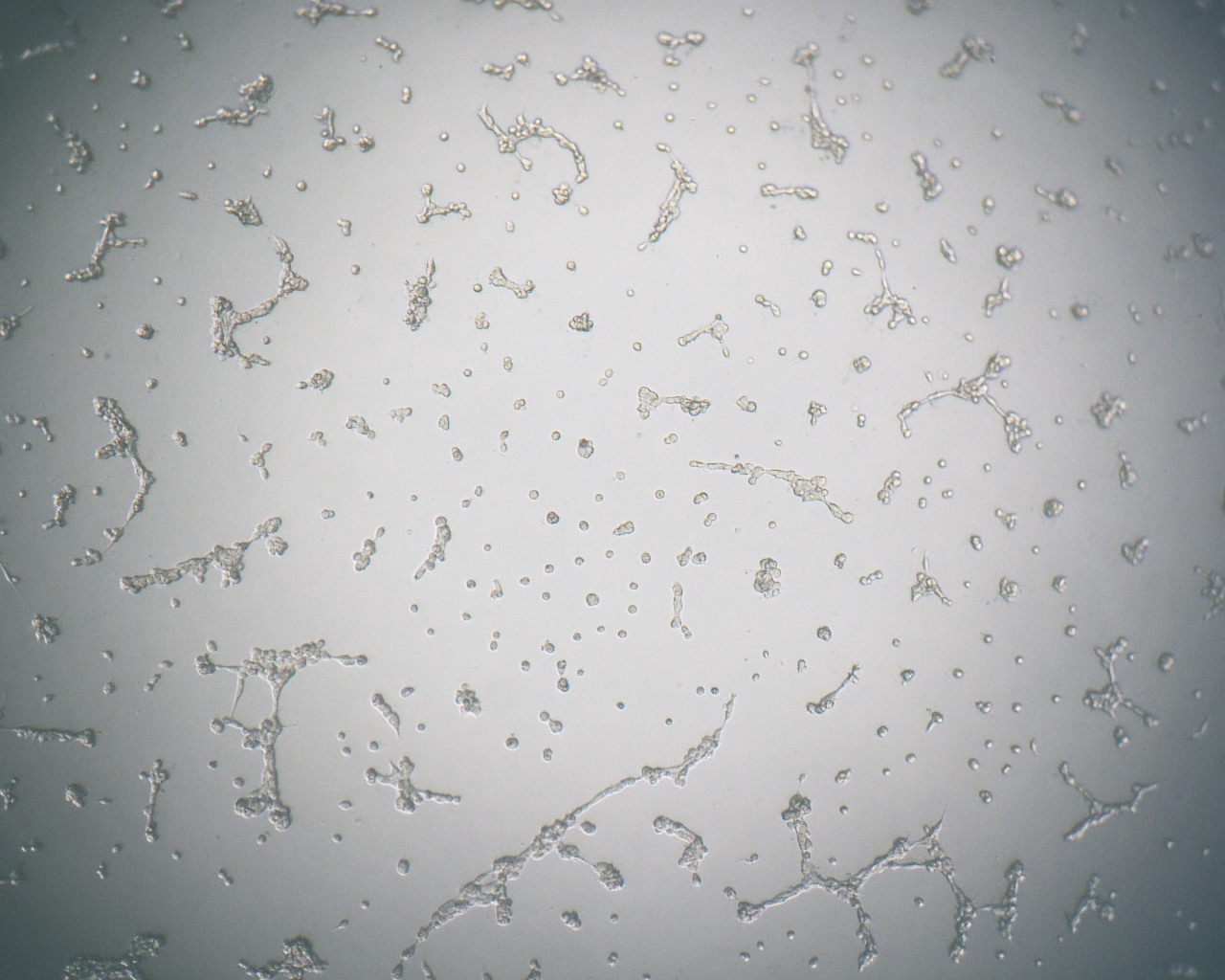

Supplement: Supplementary file 5 — Source data Fig. 3 [file 44319_2025_541_MOESM5_ESM.zip › Figure 3/3D/SFTS group VEGFA 12h.tiff]

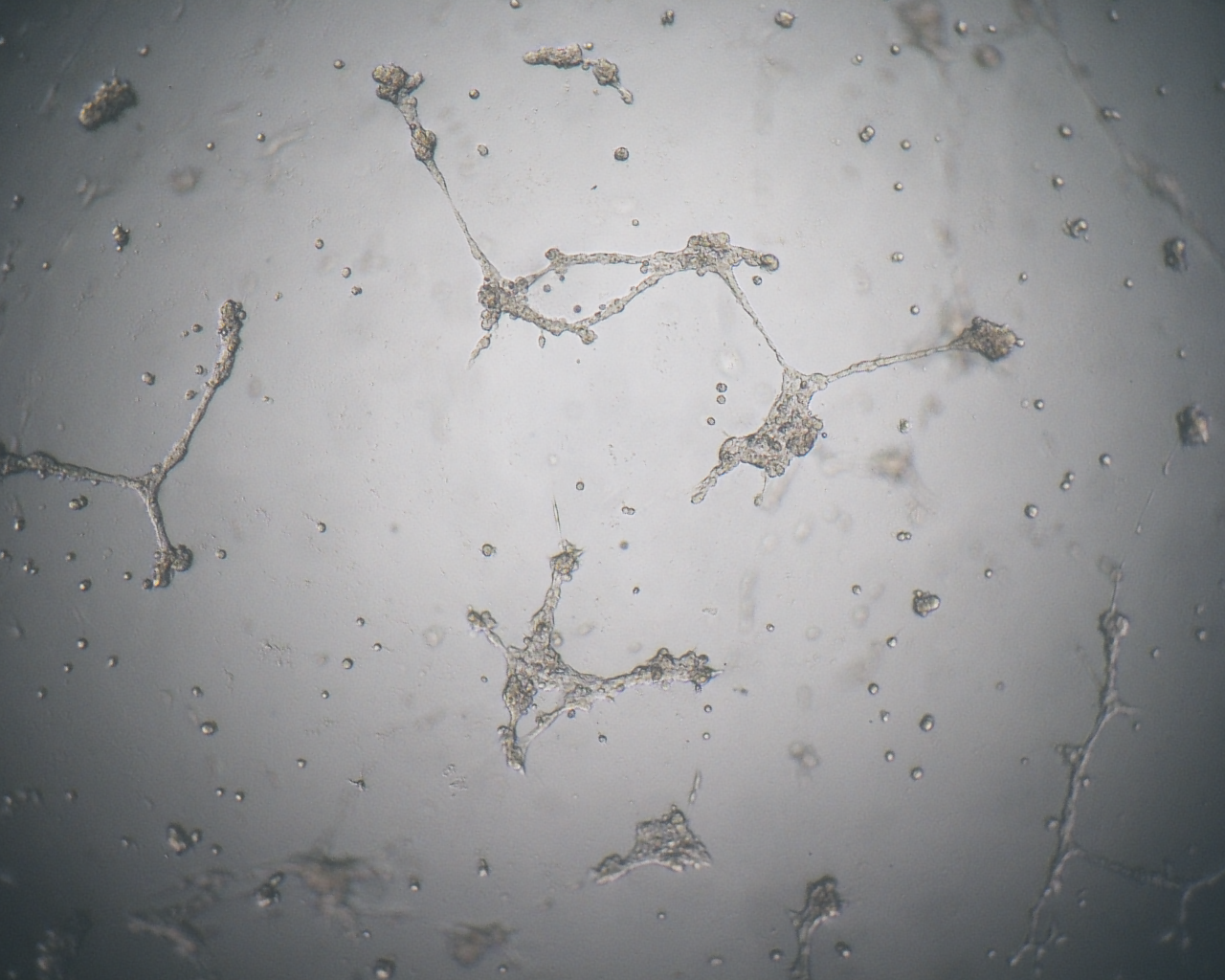

Supplement: Supplementary file 5 — Source data Fig. 3 [file 44319_2025_541_MOESM5_ESM.zip › Figure 3/3D/control group sVEGFR1 24h.tiff]

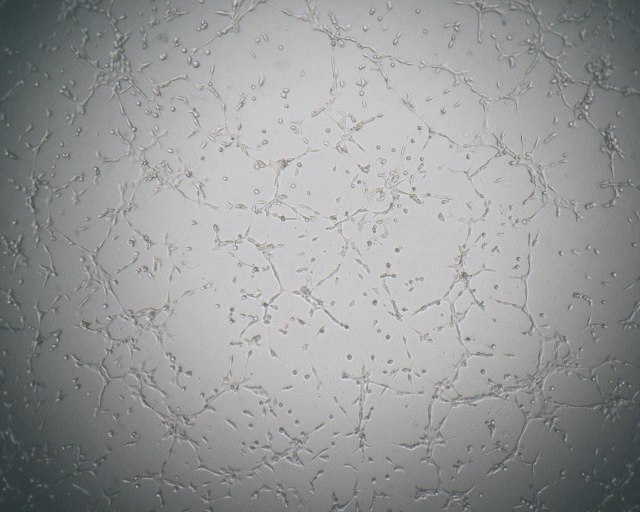

Supplement: Supplementary file 5 — Source data Fig. 3 [file 44319_2025_541_MOESM5_ESM.zip › Figure 3/3D/control group none 12h.tiff]

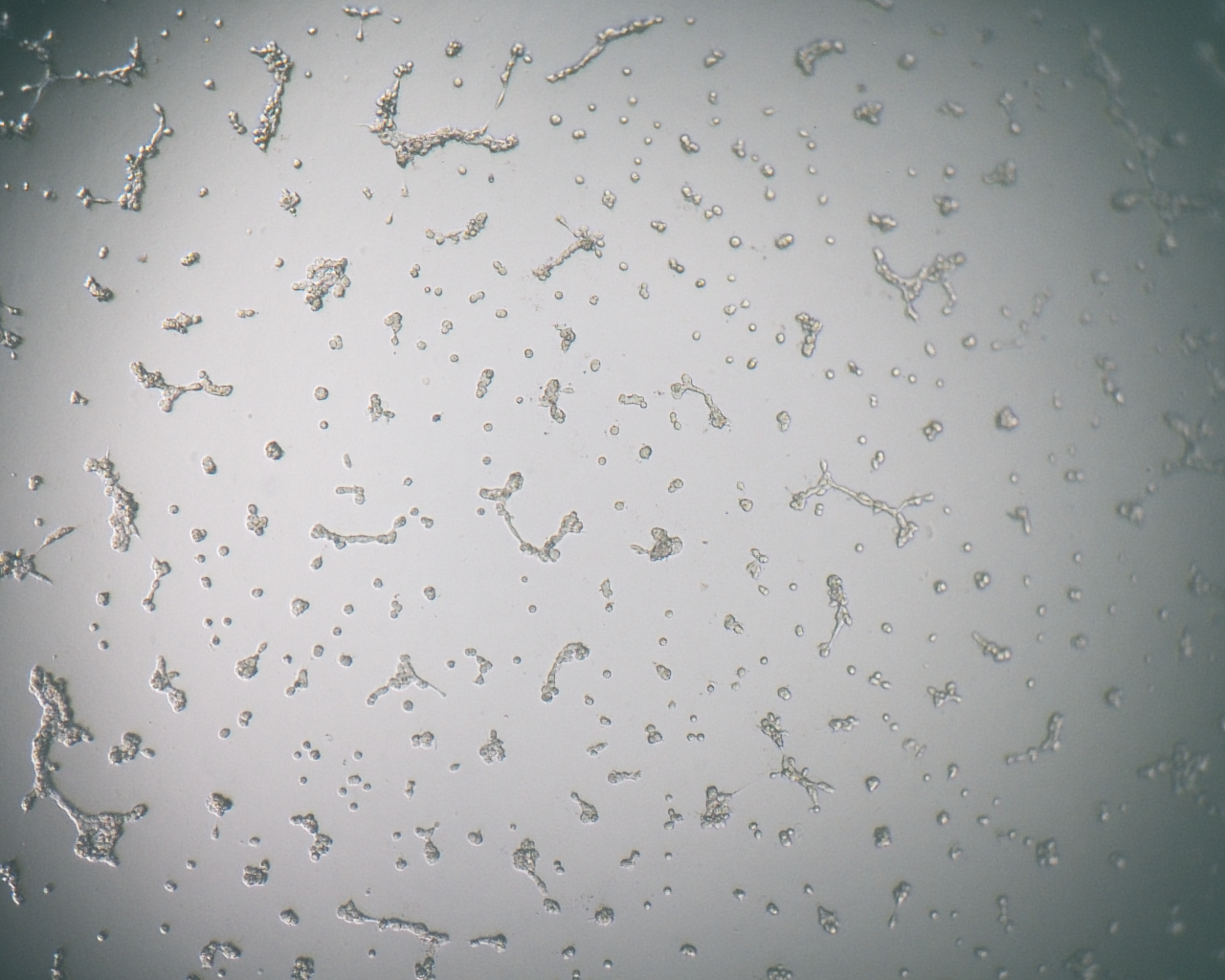

Supplement: Supplementary file 5 — Source data Fig. 3 [file 44319_2025_541_MOESM5_ESM.zip › Figure 3/3D/SFTS group none 12h.tiff]

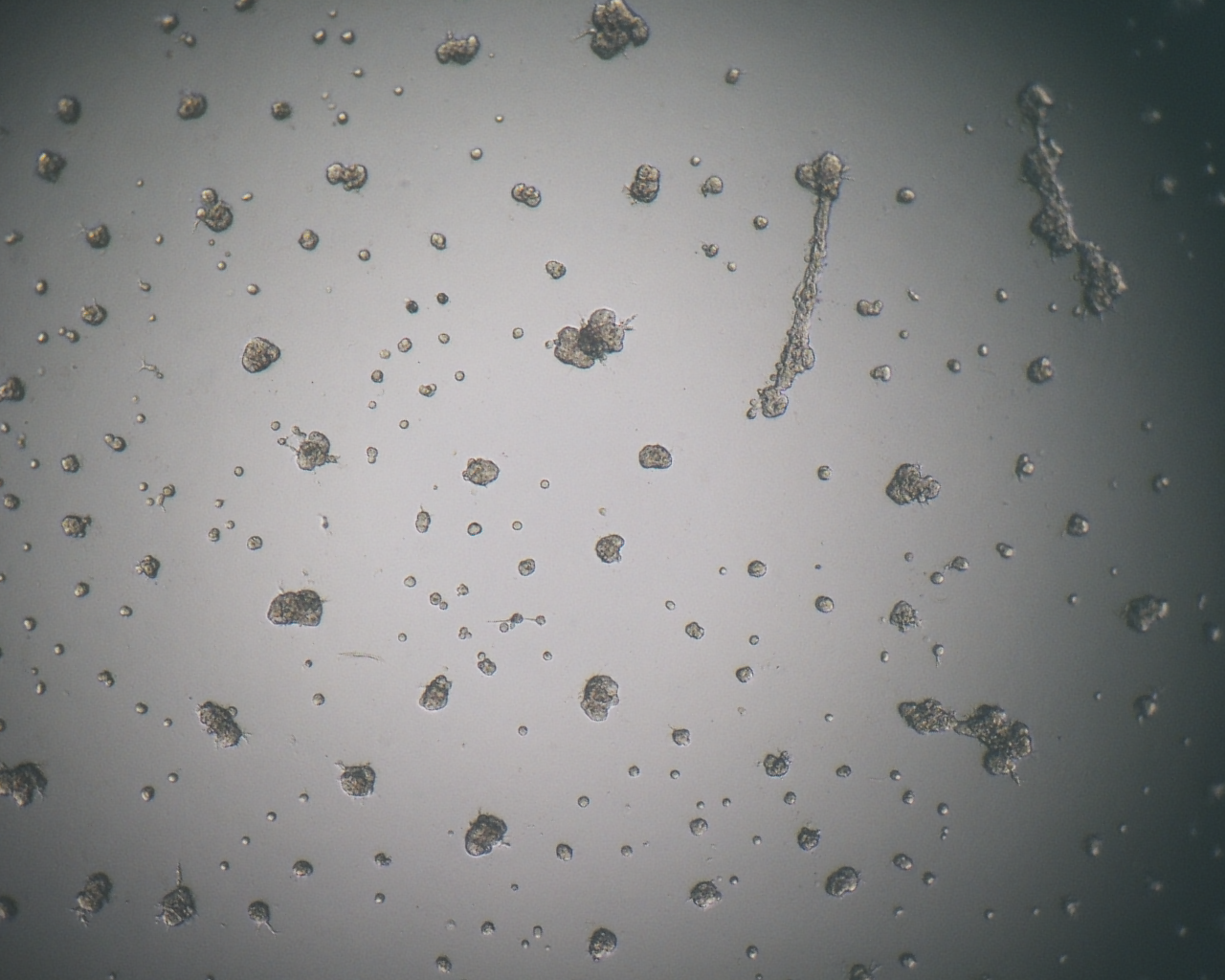

Supplement: Supplementary file 5 — Source data Fig. 3 [file 44319_2025_541_MOESM5_ESM.zip › Figure 3/3D/SFTS group VEGFA 24h.tiff]

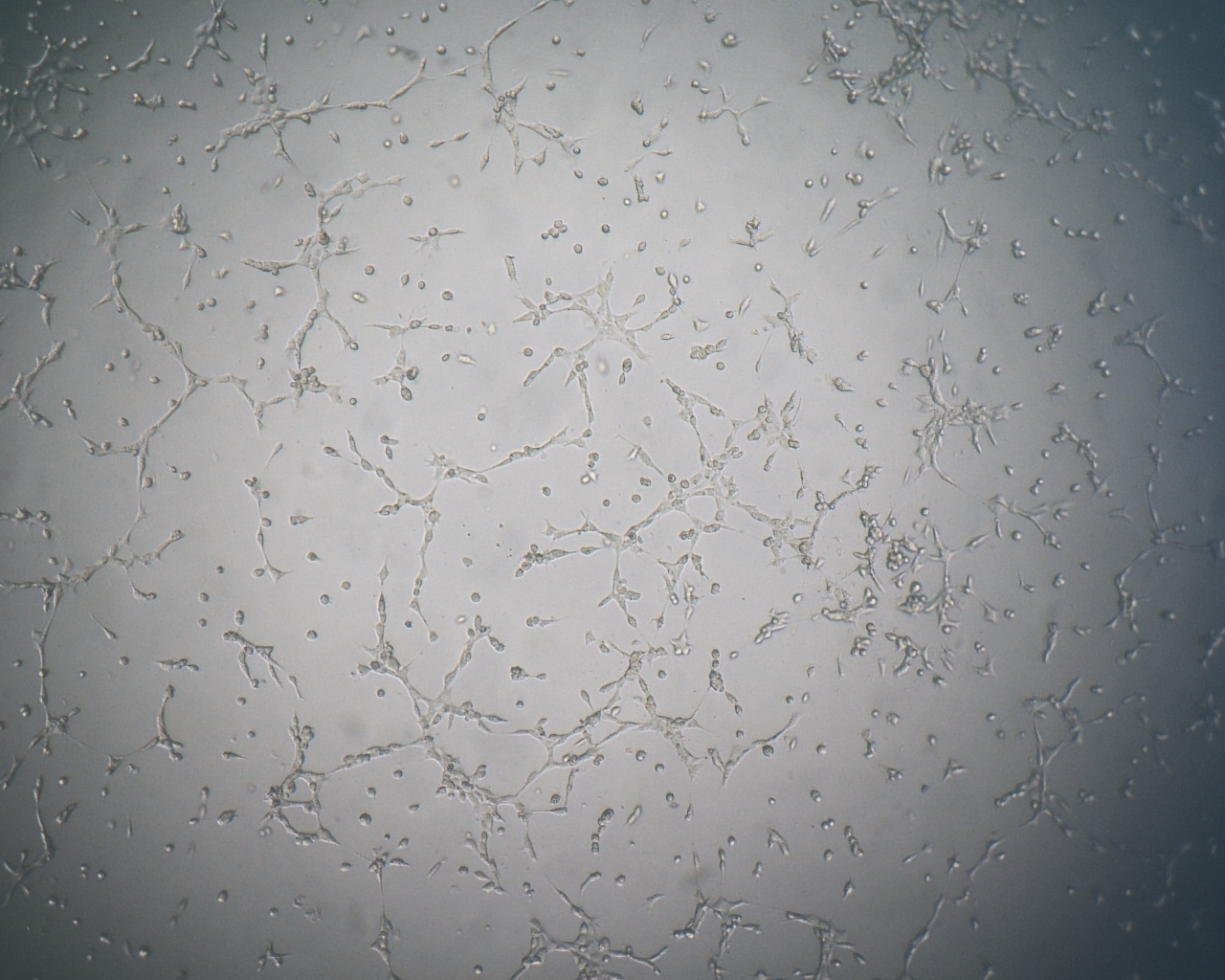

Supplement: Supplementary file 5 — Source data Fig. 3 [file 44319_2025_541_MOESM5_ESM.zip › Figure 3/3D/control group sVEGFR1 12h.tiff]

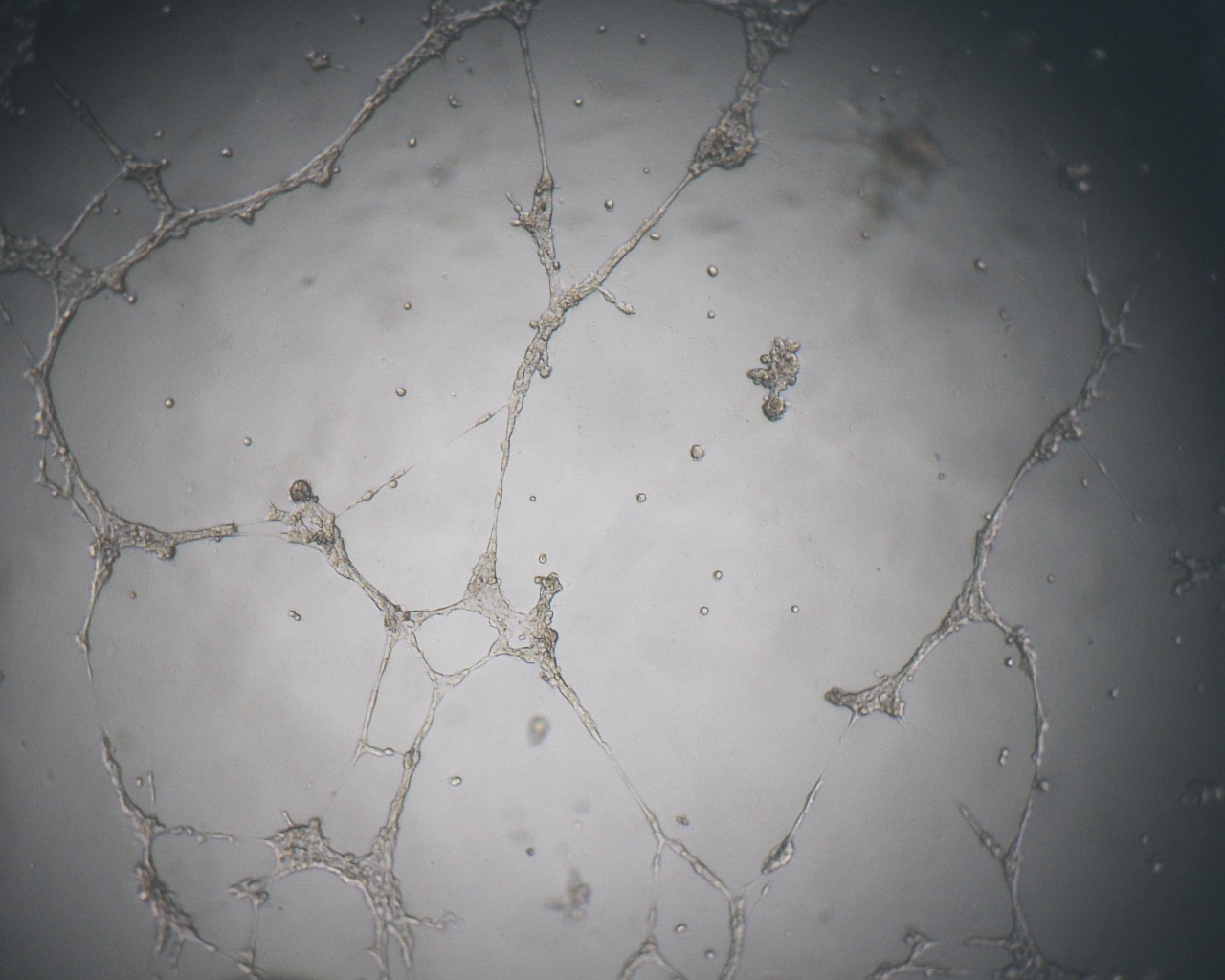

Supplement: Supplementary file 5 — Source data Fig. 3 [file 44319_2025_541_MOESM5_ESM.zip › Figure 3/3D/control group none 24h.tiff]

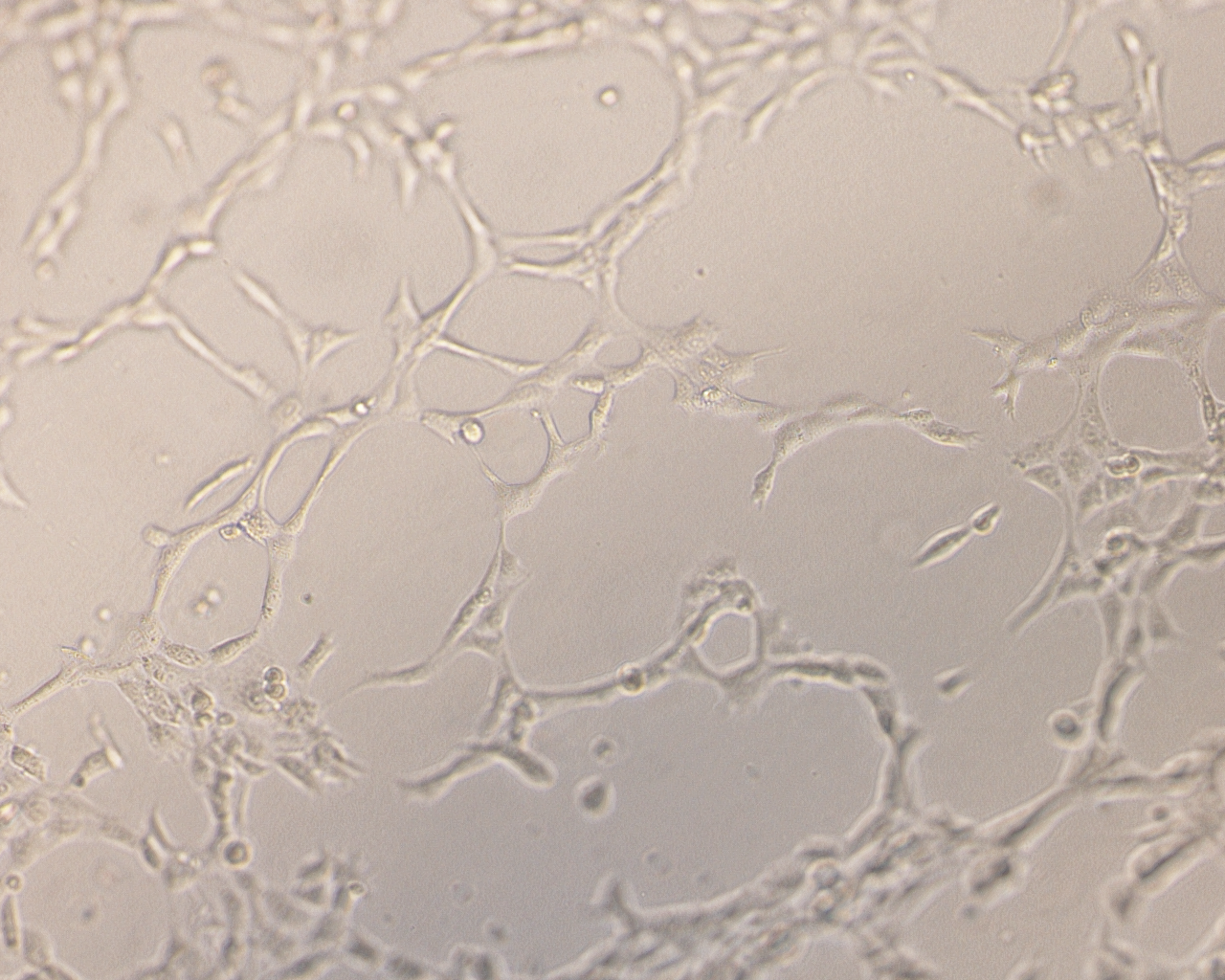

Supplement: Supplementary file 5 — Source data Fig. 3 [file 44319_2025_541_MOESM5_ESM.zip › Figure 3/3A/control group.tiff]

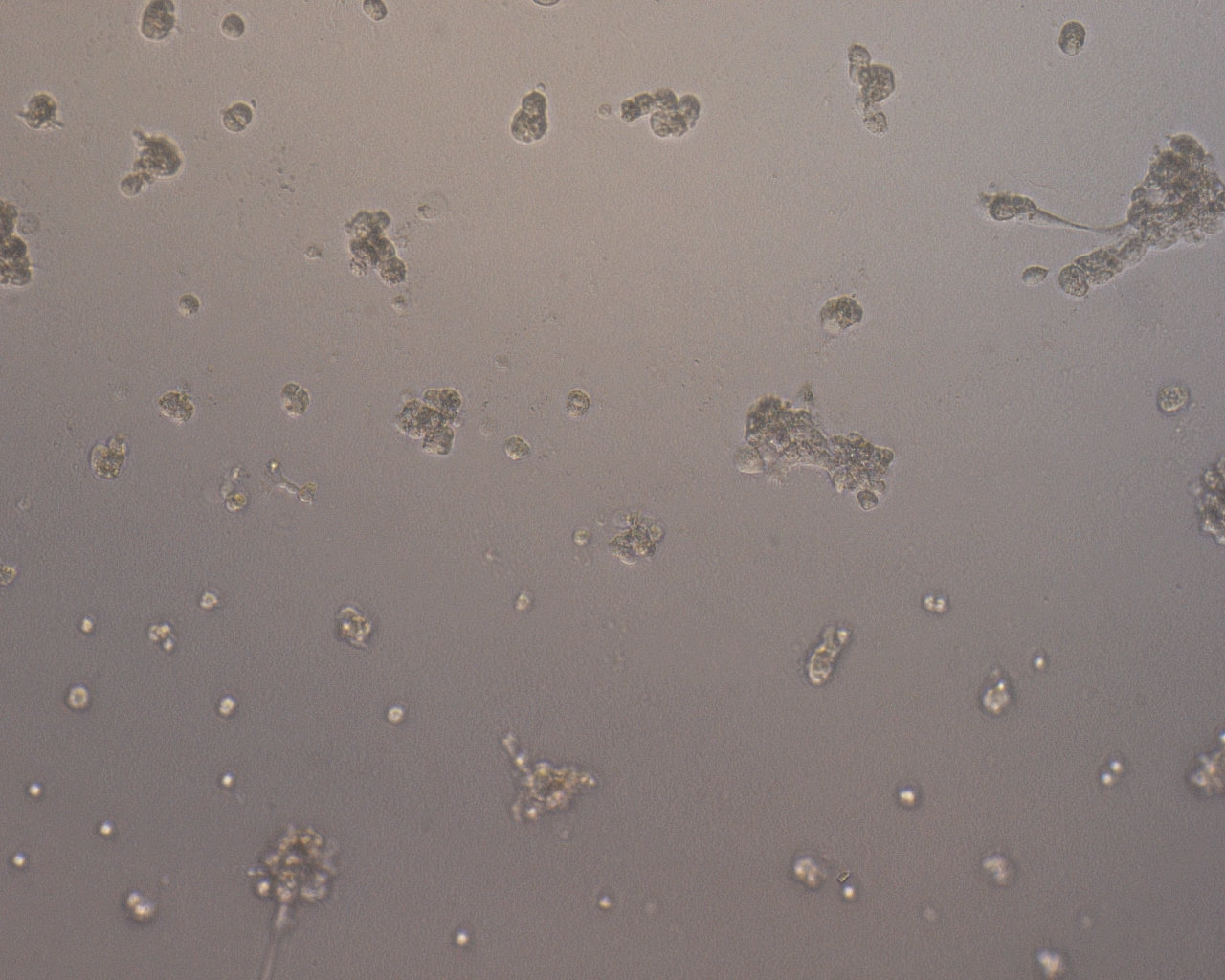

Supplement: Supplementary file 5 — Source data Fig. 3 [file 44319_2025_541_MOESM5_ESM.zip › Figure 3/3A/mild group.tiff]

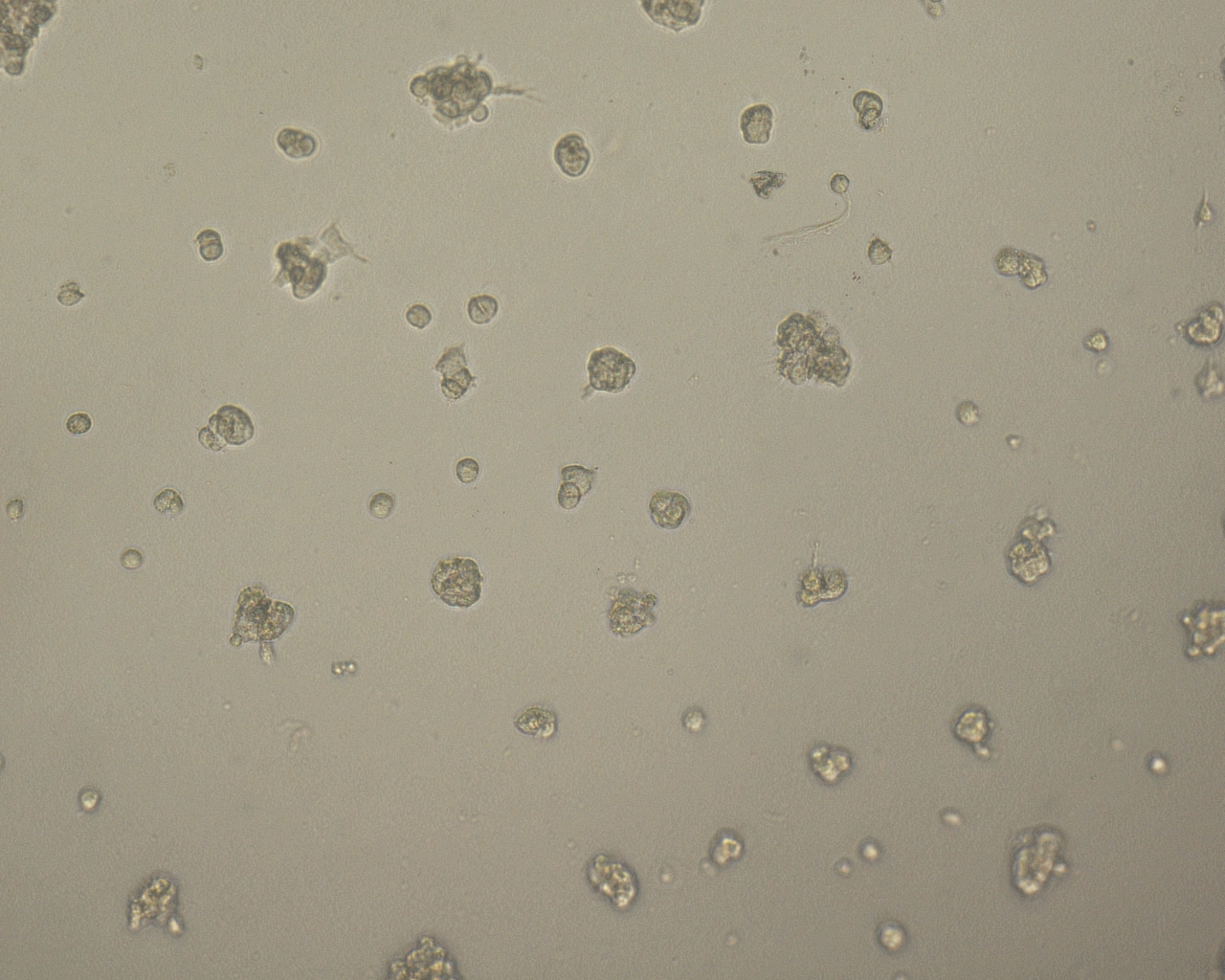

Supplement: Supplementary file 5 — Source data Fig. 3 [file 44319_2025_541_MOESM5_ESM.zip › Figure 3/3A/severe group.tiff]

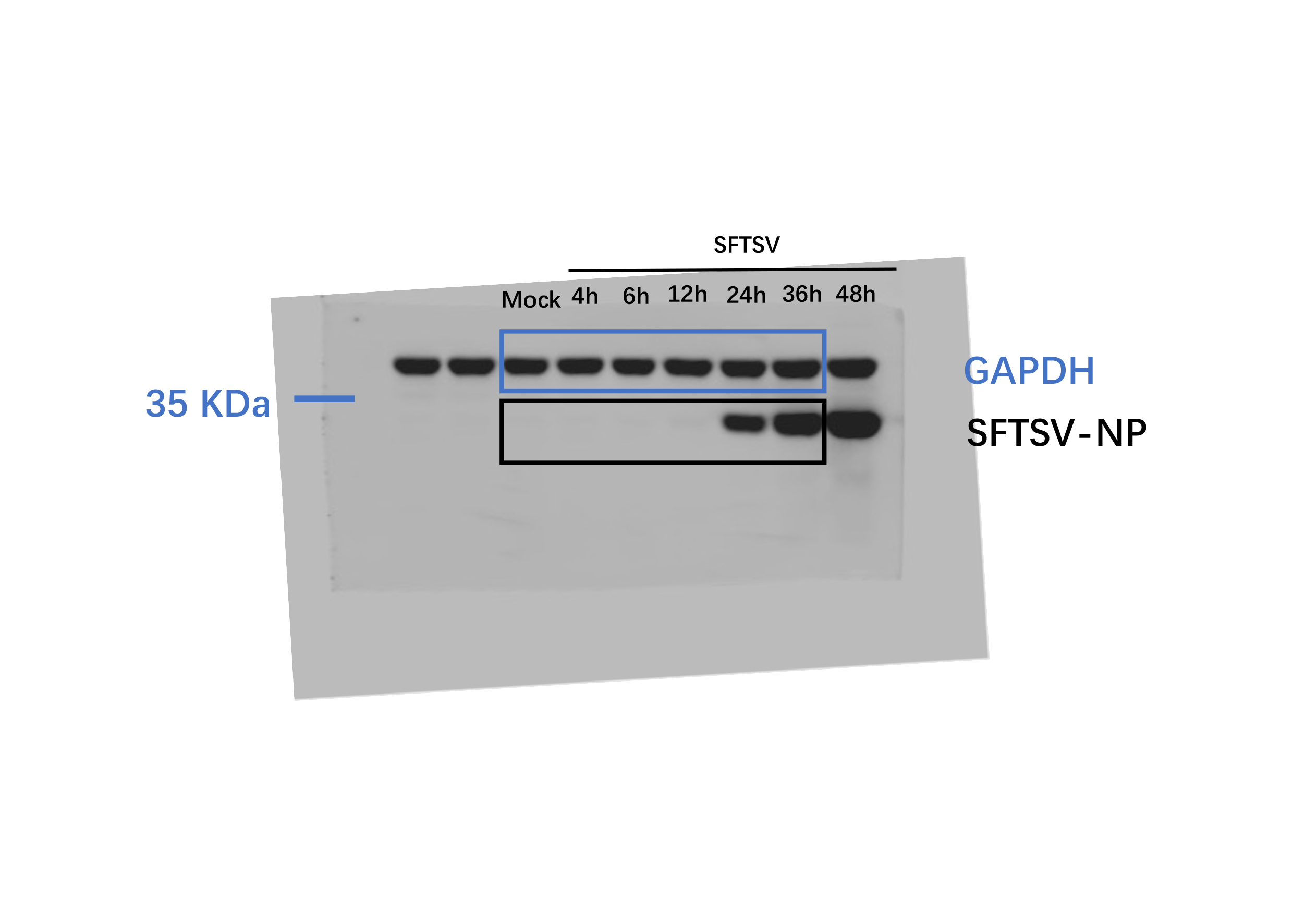

Supplement: Supplementary file 6 — Source data Fig. 4 [file 44319_2025_541_MOESM6_ESM.zip › Figure 4/4C/western NP and GAPDH.tiff]

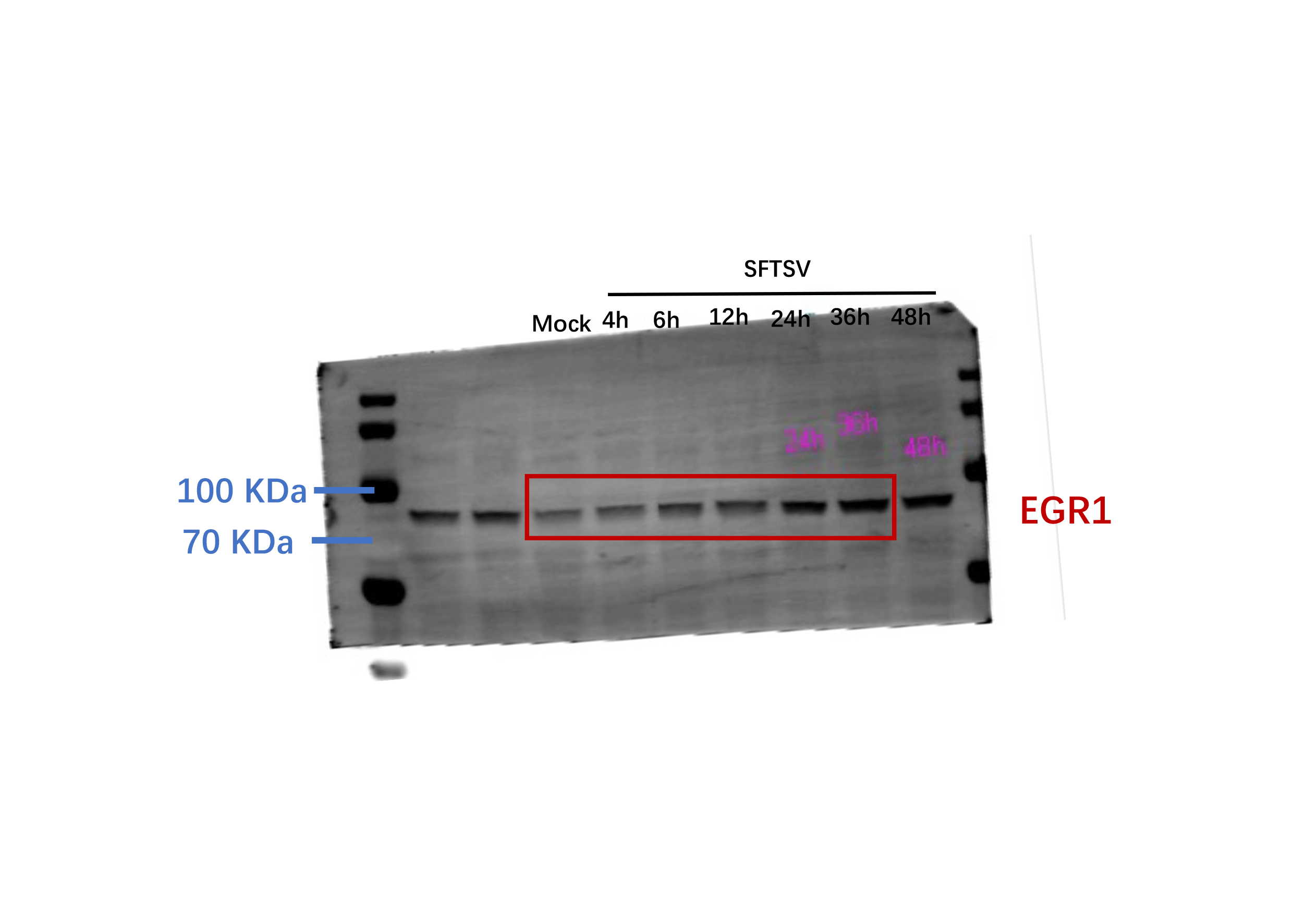

Supplement: Supplementary file 6 — Source data Fig. 4 [file 44319_2025_541_MOESM6_ESM.zip › Figure 4/4C/western EGR1.tiff]

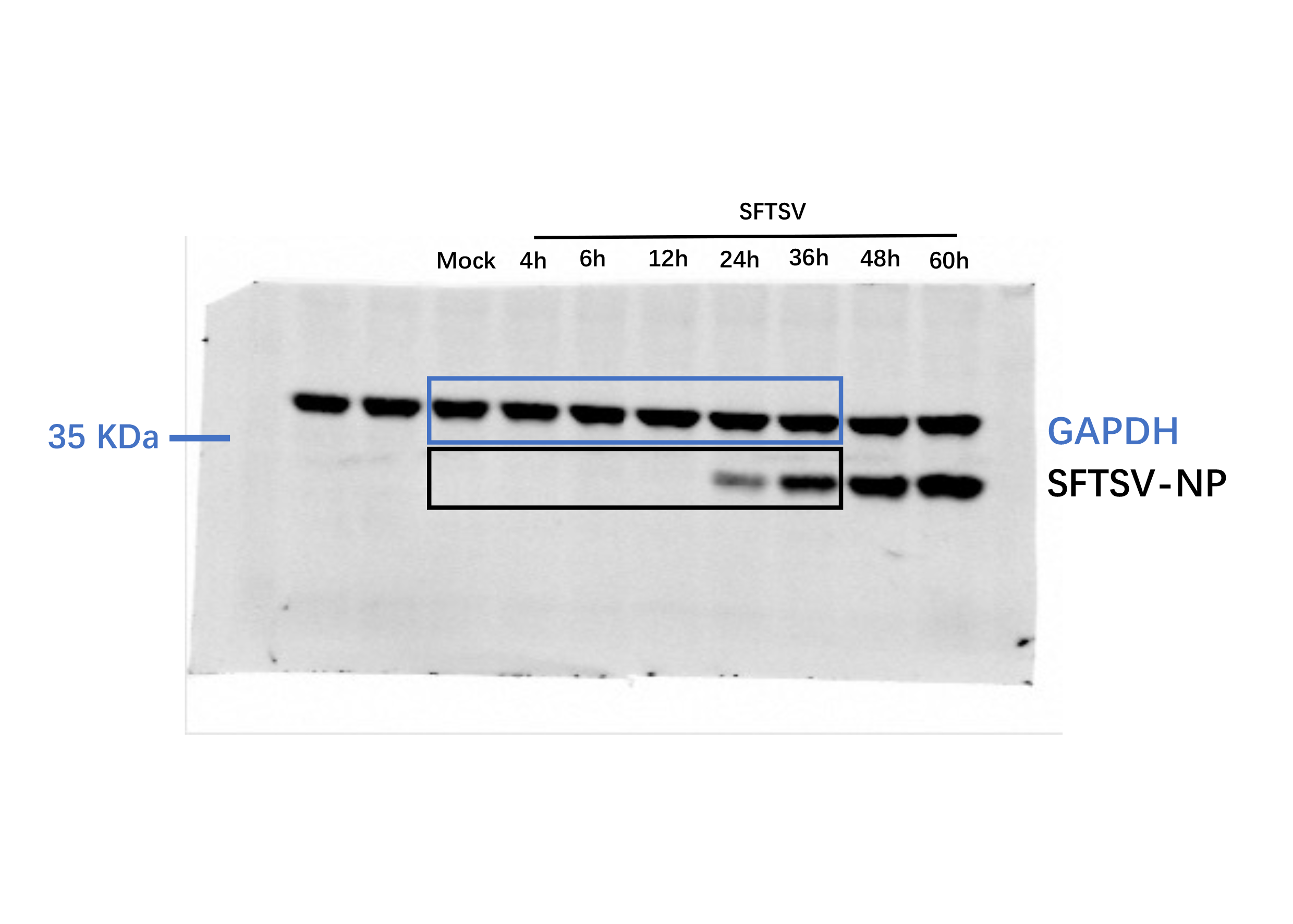

Supplement: Supplementary file 6 — Source data Fig. 4 [file 44319_2025_541_MOESM6_ESM.zip › Figure 4/4D/western NP and GAPDH.tiff]

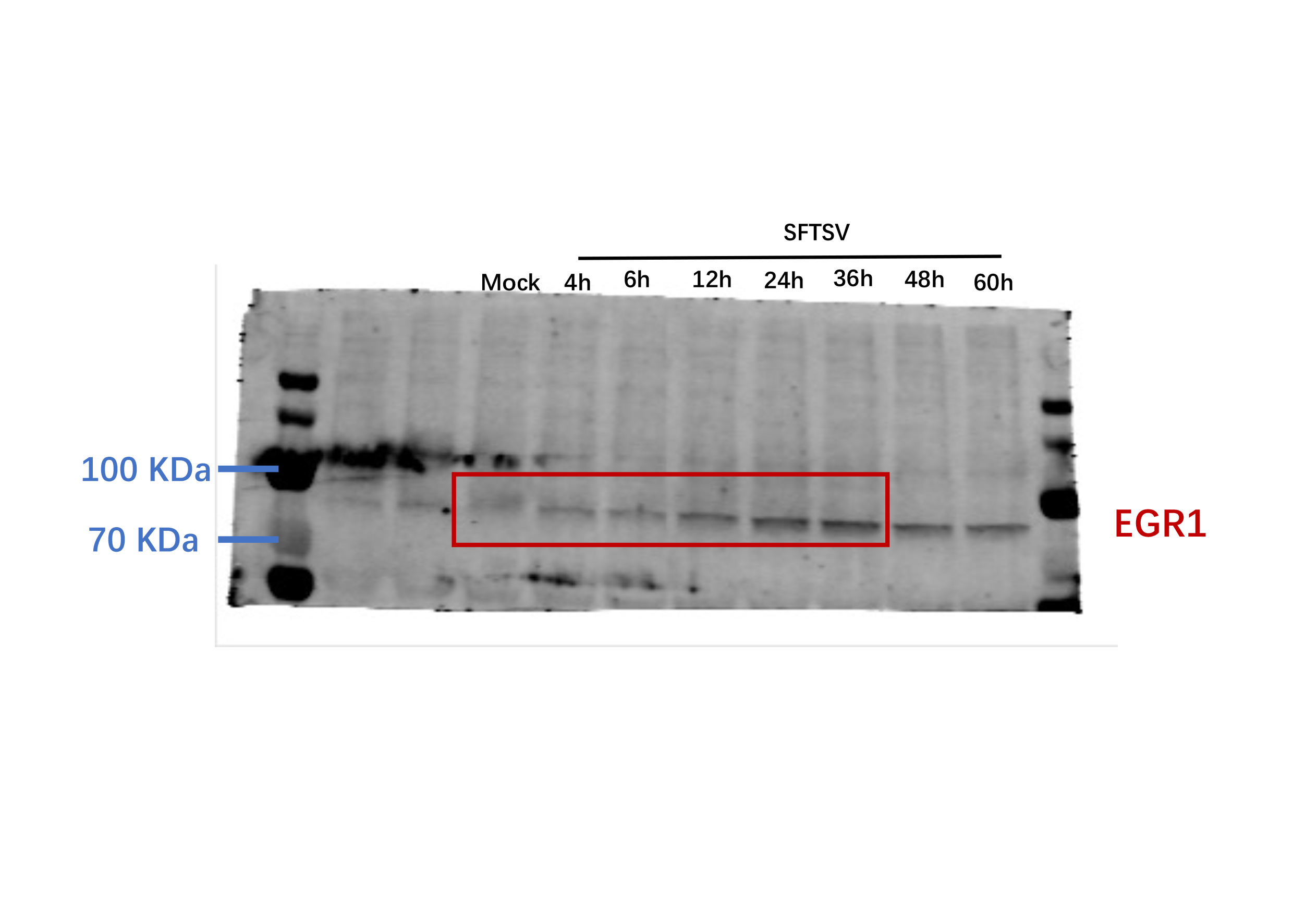

Supplement: Supplementary file 6 — Source data Fig. 4 [file 44319_2025_541_MOESM6_ESM.zip › Figure 4/4D/western EGR1.tiff]

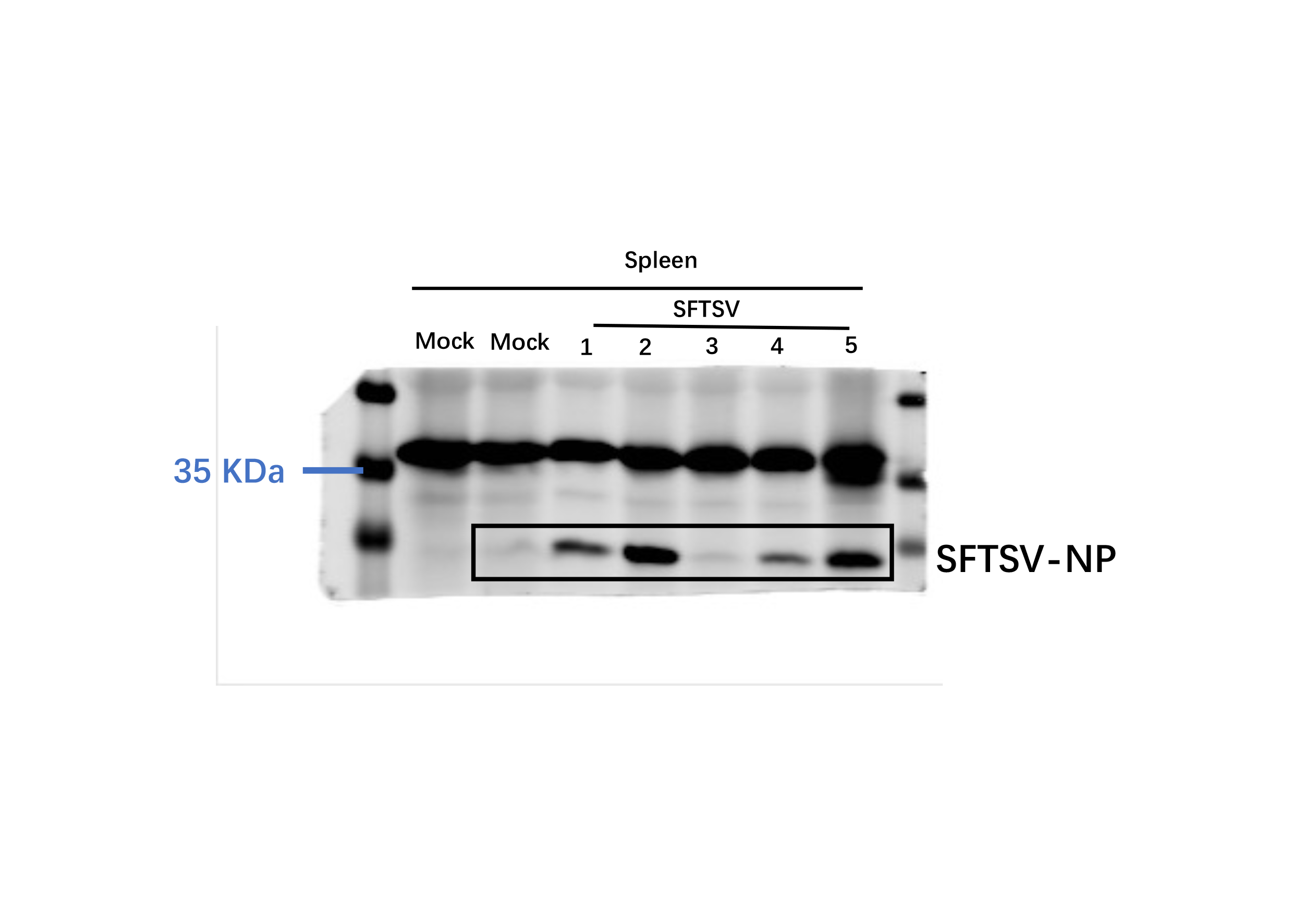

Supplement: Supplementary file 6 — Source data Fig. 4 [file 44319_2025_541_MOESM6_ESM.zip › Figure 4/4H/western NP.tiff]

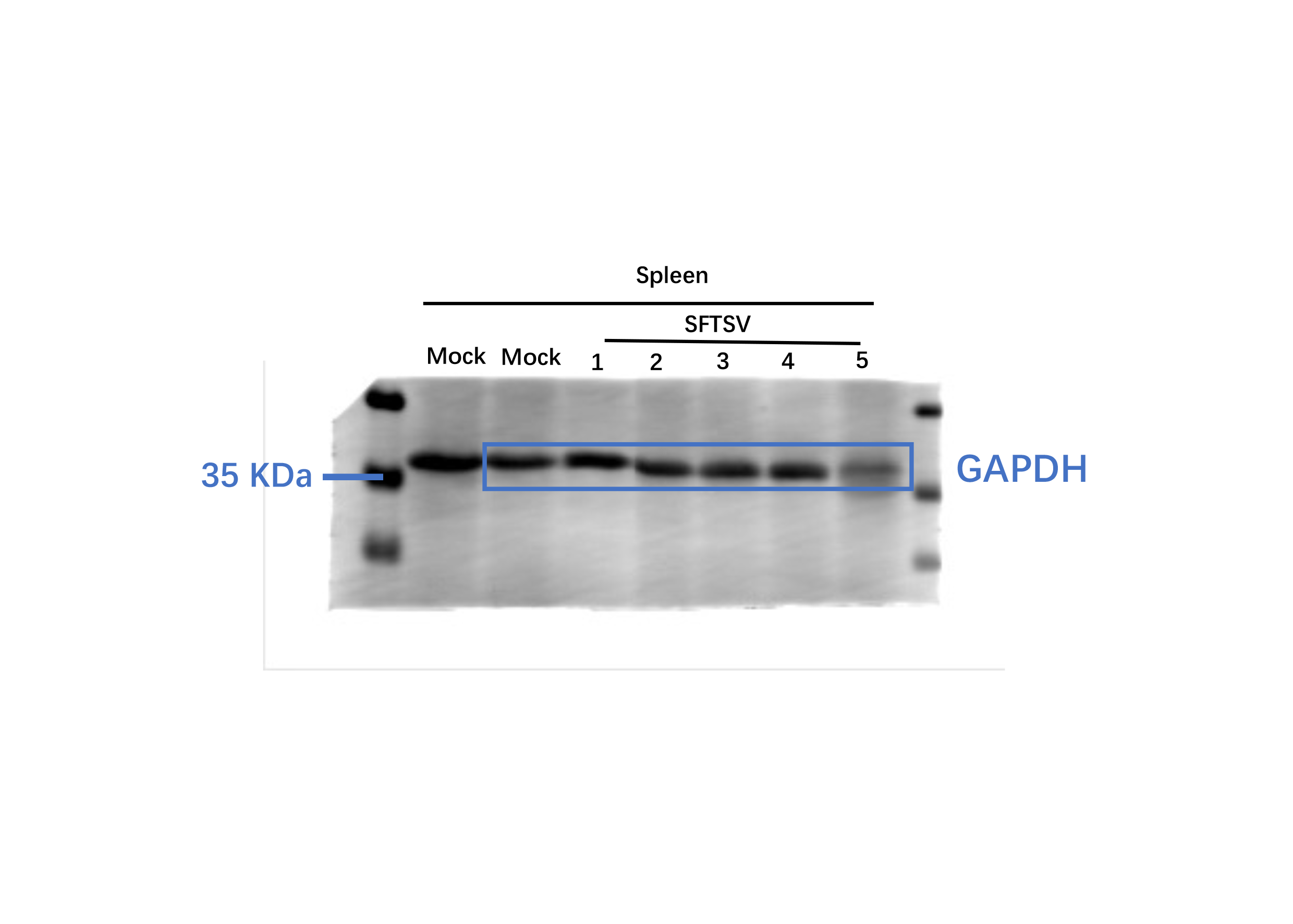

Supplement: Supplementary file 6 — Source data Fig. 4 [file 44319_2025_541_MOESM6_ESM.zip › Figure 4/4H/western GAPDH.tiff]

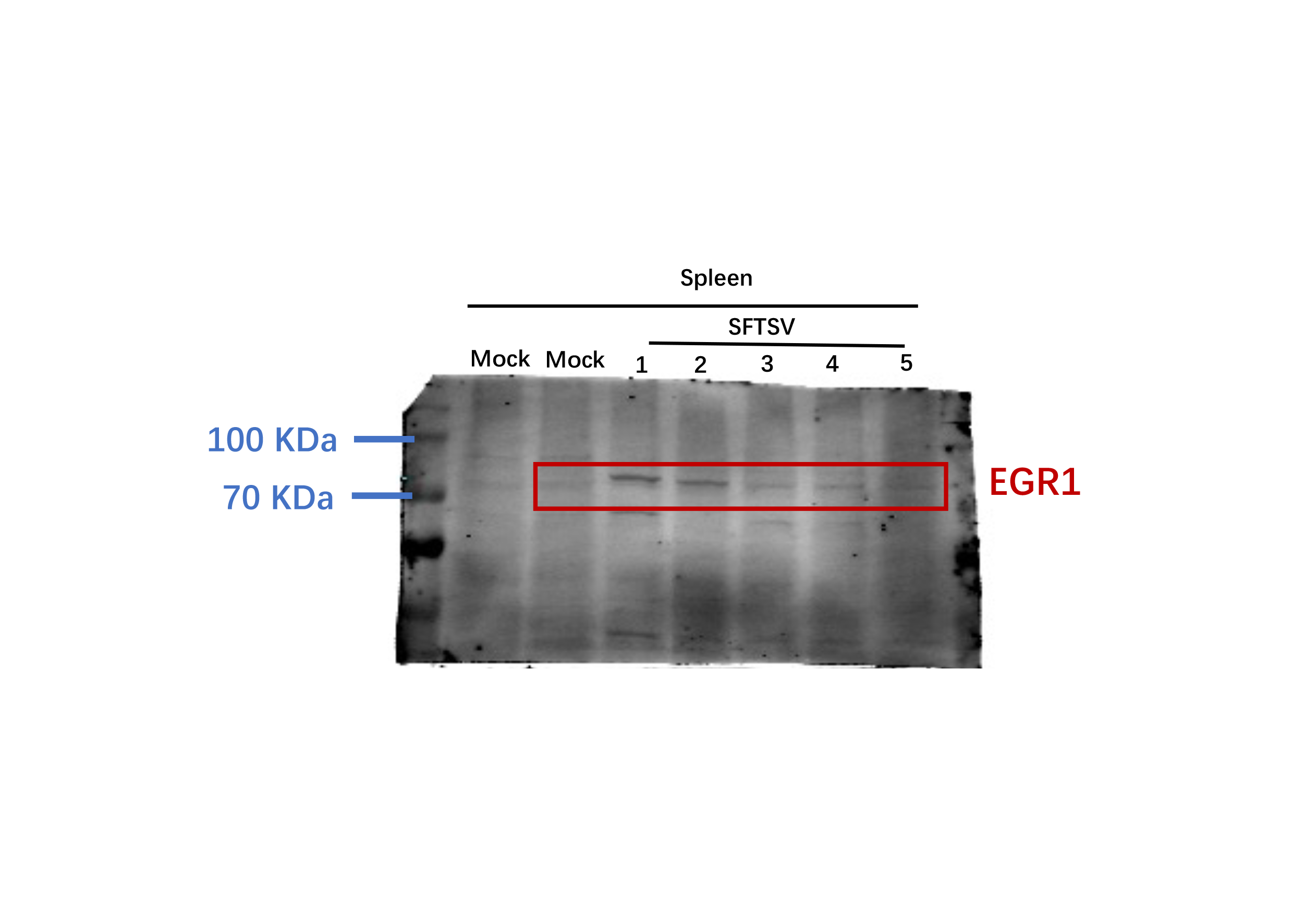

Supplement: Supplementary file 6 — Source data Fig. 4 [file 44319_2025_541_MOESM6_ESM.zip › Figure 4/4H/western EGR1.tiff]

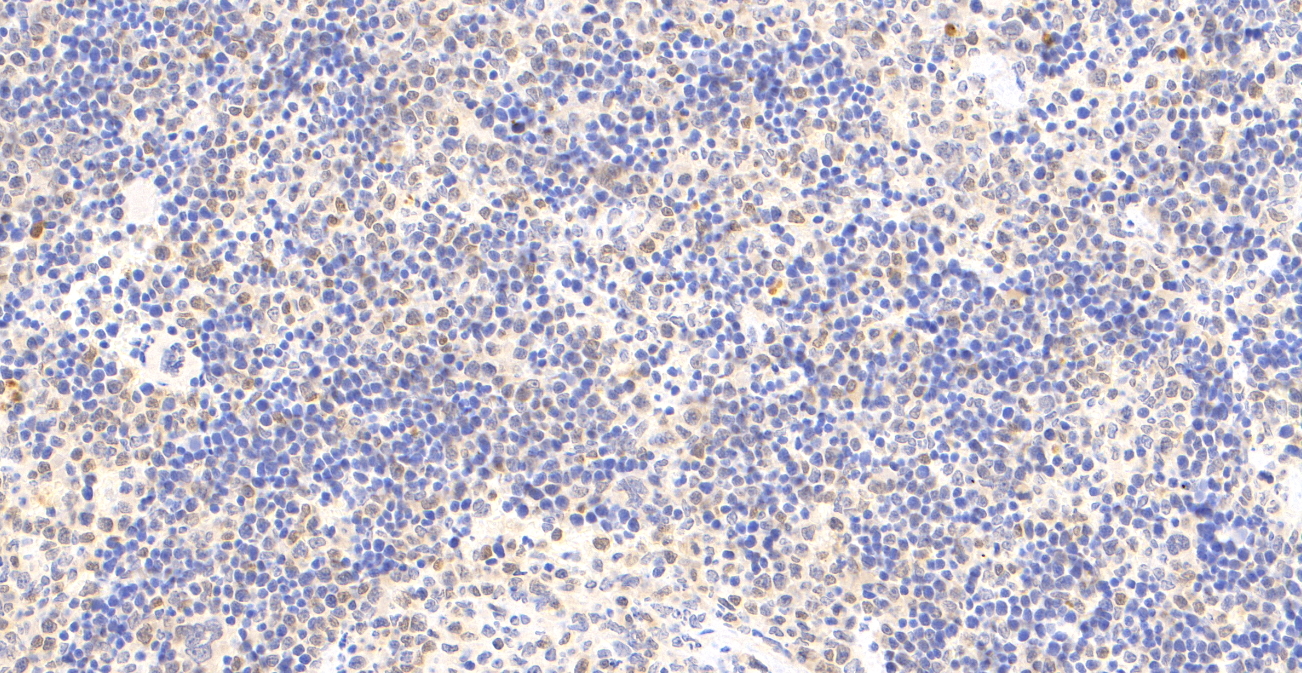

Supplement: Supplementary file 6 — Source data Fig. 4 [file 44319_2025_541_MOESM6_ESM.zip › Figure 4/4I/Mock_spleen EGR1.tiff]

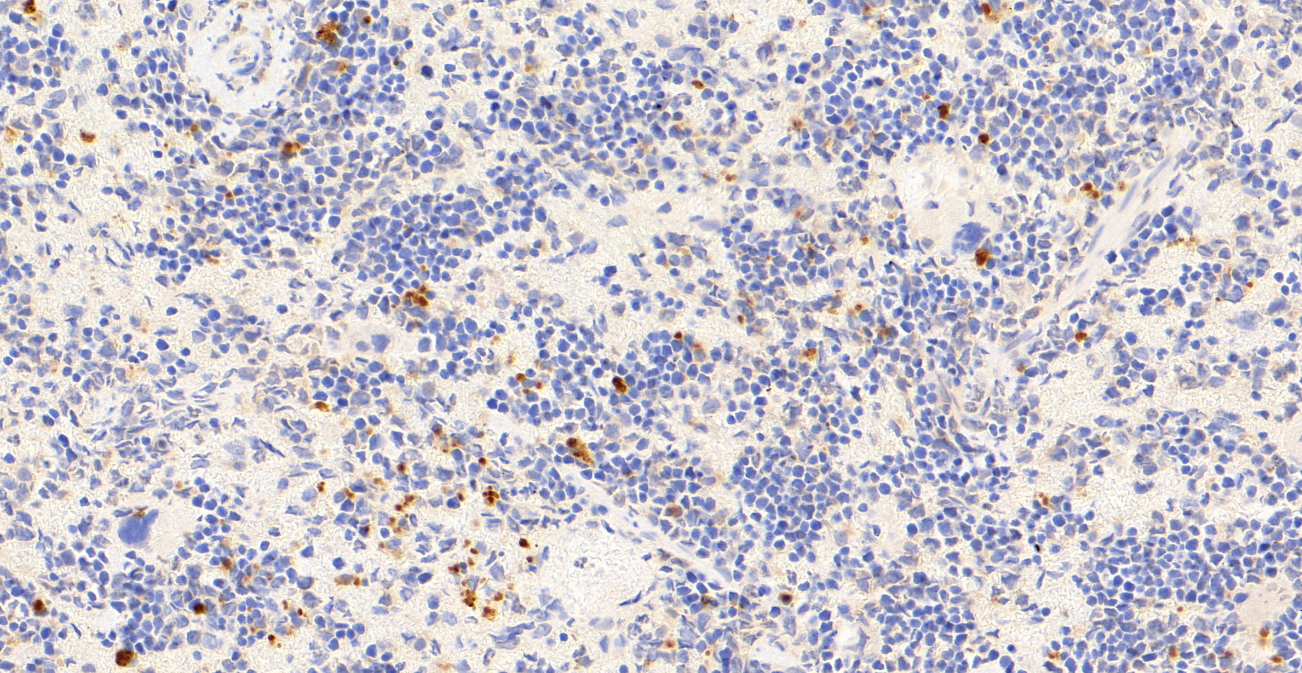

Supplement: Supplementary file 6 — Source data Fig. 4 [file 44319_2025_541_MOESM6_ESM.zip › Figure 4/4I/SFTSV_spleen EGR1.tiff]

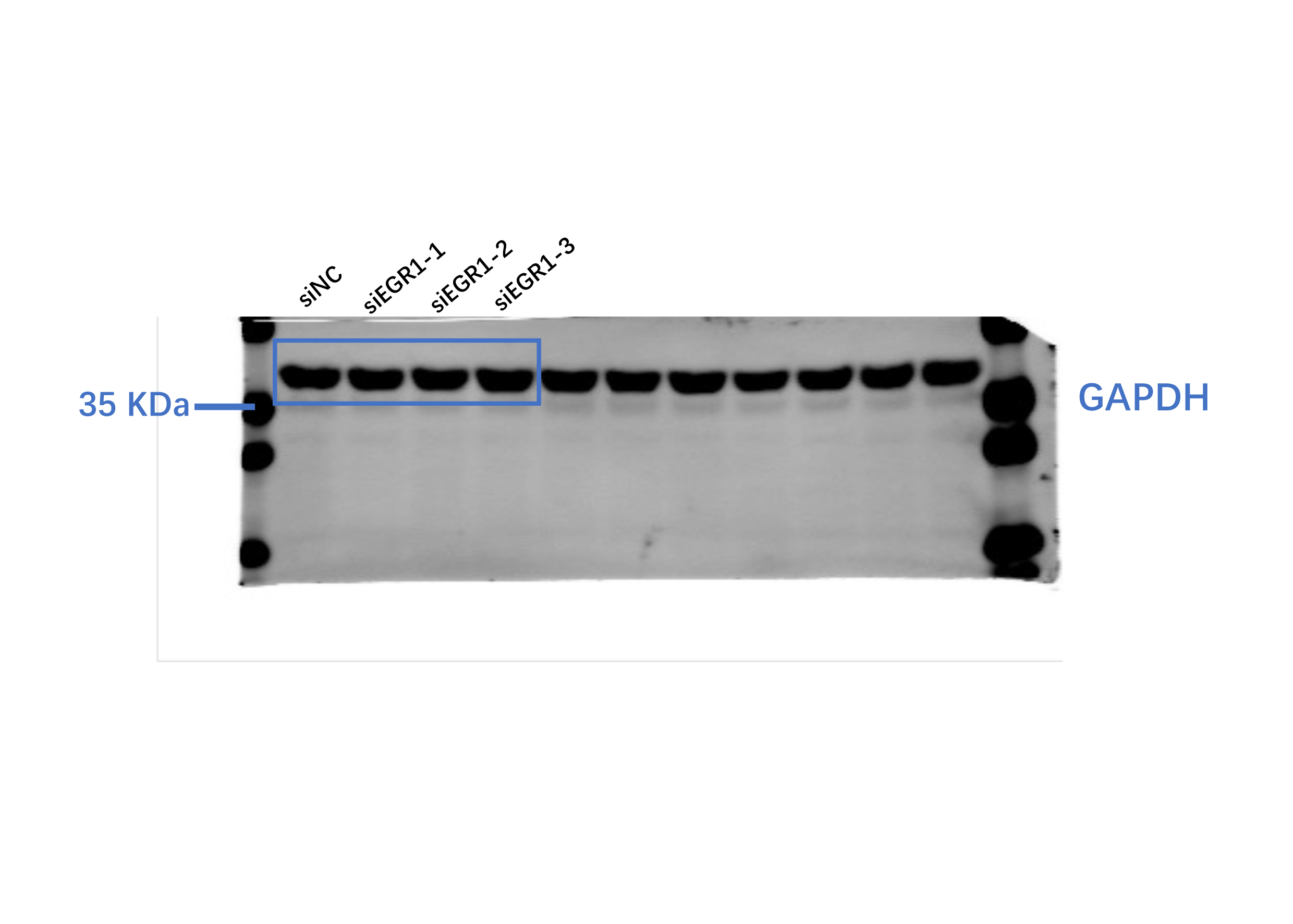

Supplement: Supplementary file 7 — Source data Fig. 5 [file 44319_2025_541_MOESM7_ESM.zip › Figure 5/5G/western GAPDH.tiff]

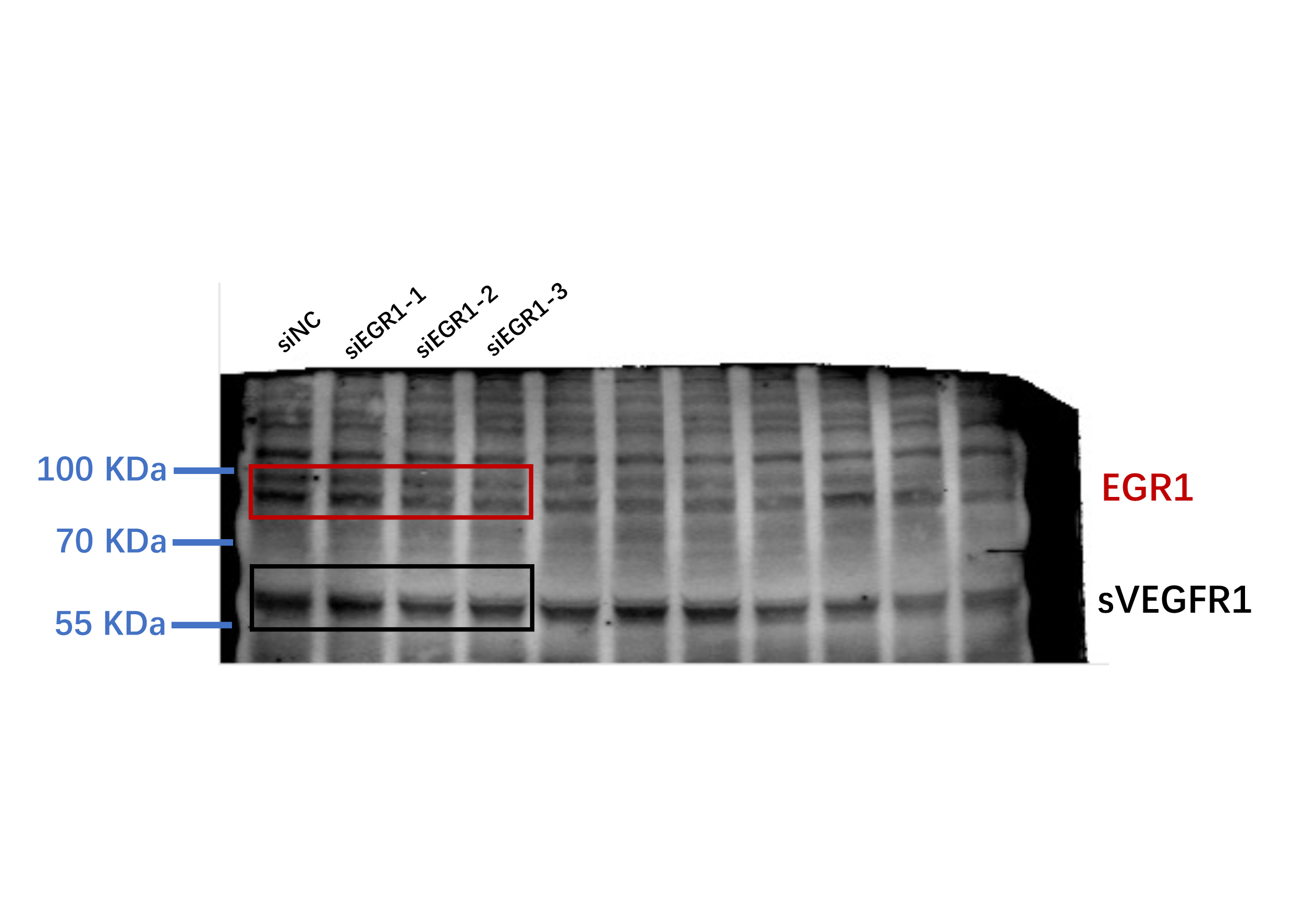

Supplement: Supplementary file 7 — Source data Fig. 5 [file 44319_2025_541_MOESM7_ESM.zip › Figure 5/5G/western EGR1 and sVEGFR1.tiff]

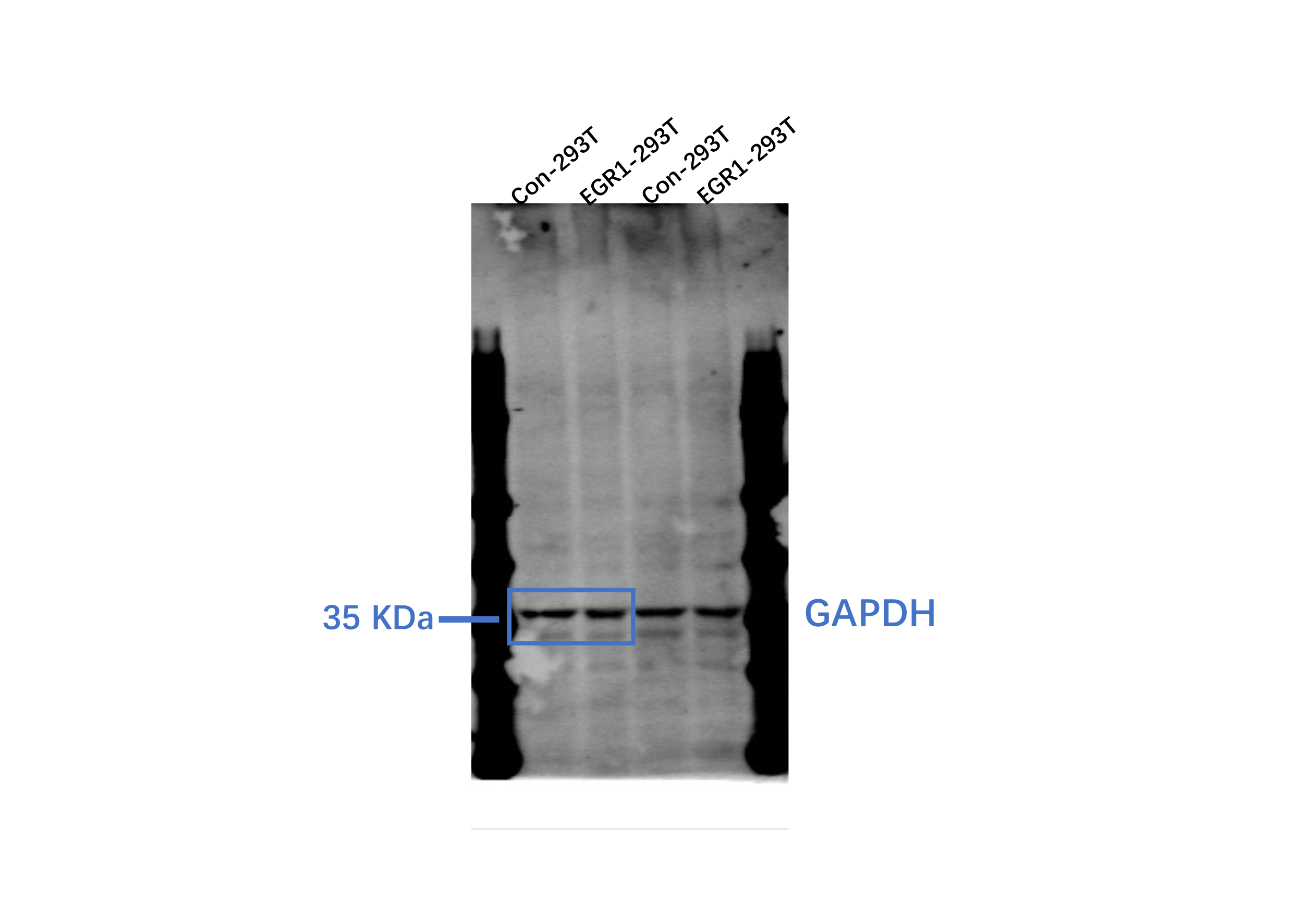

Supplement: Supplementary file 7 — Source data Fig. 5 [file 44319_2025_541_MOESM7_ESM.zip › Figure 5/5E/western GAPDH.tiff]

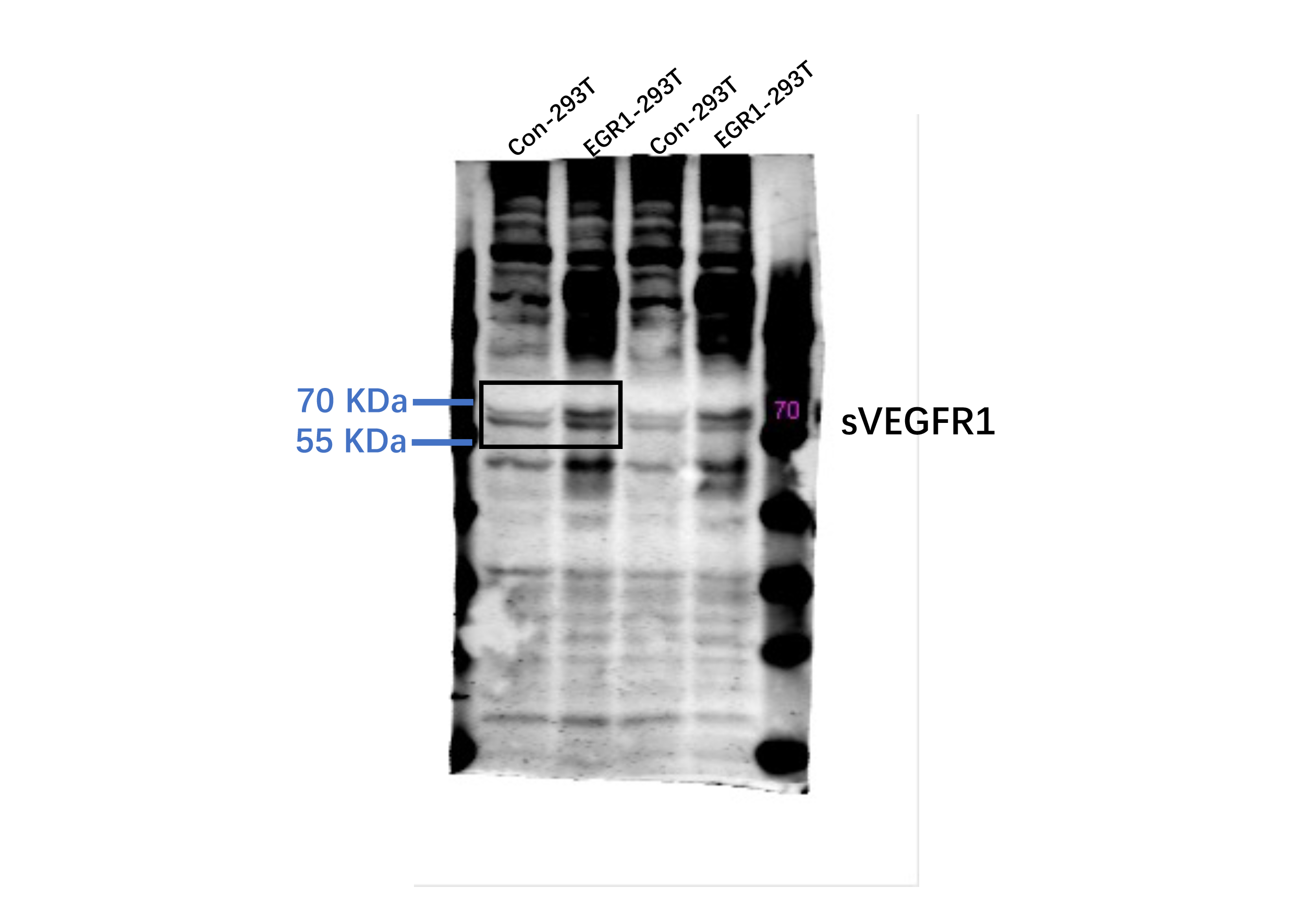

Supplement: Supplementary file 7 — Source data Fig. 5 [file 44319_2025_541_MOESM7_ESM.zip › Figure 5/5E/western sVEGFR1.tiff]

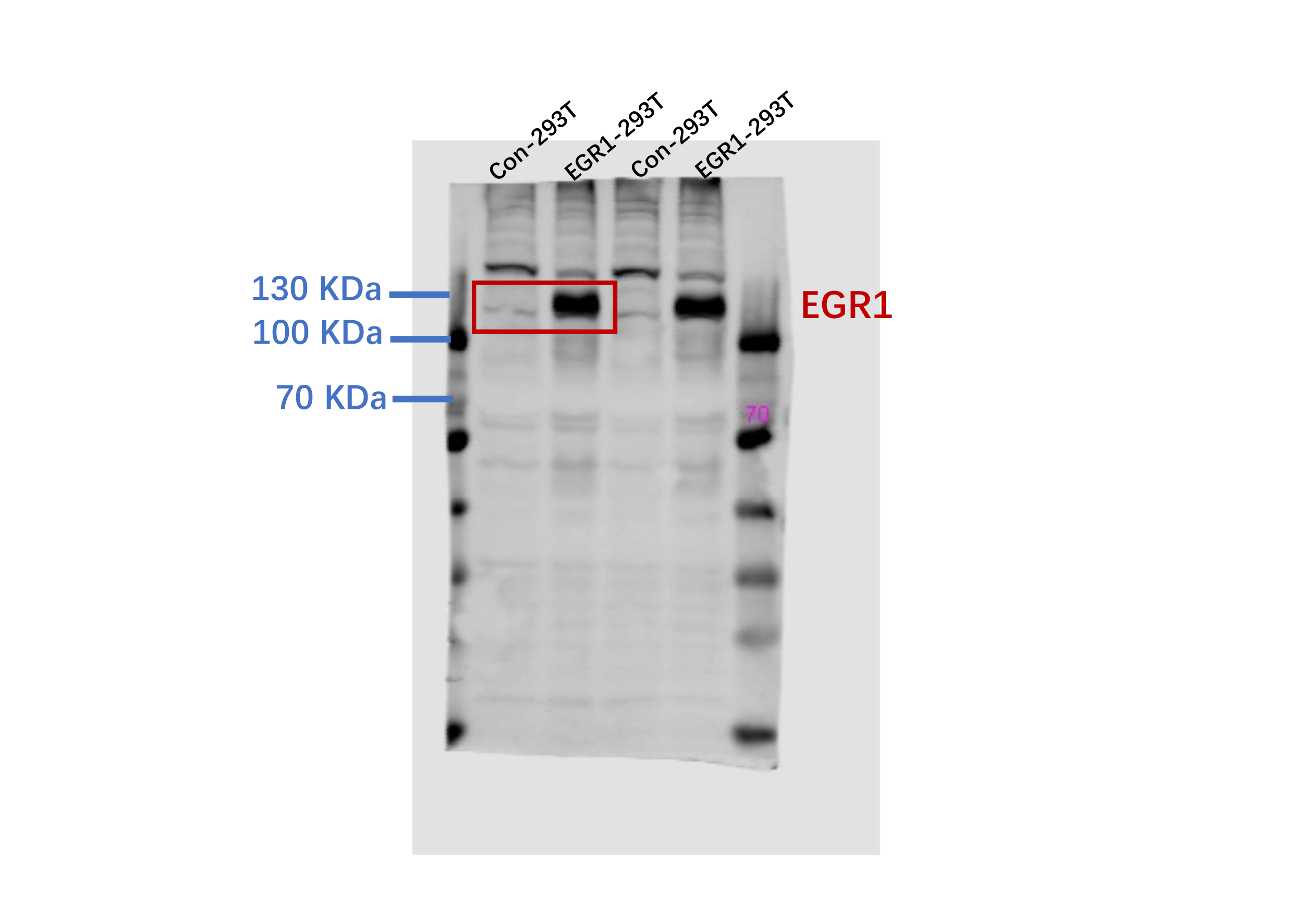

Supplement: Supplementary file 7 — Source data Fig. 5 [file 44319_2025_541_MOESM7_ESM.zip › Figure 5/5E/western EGR1.tiff]

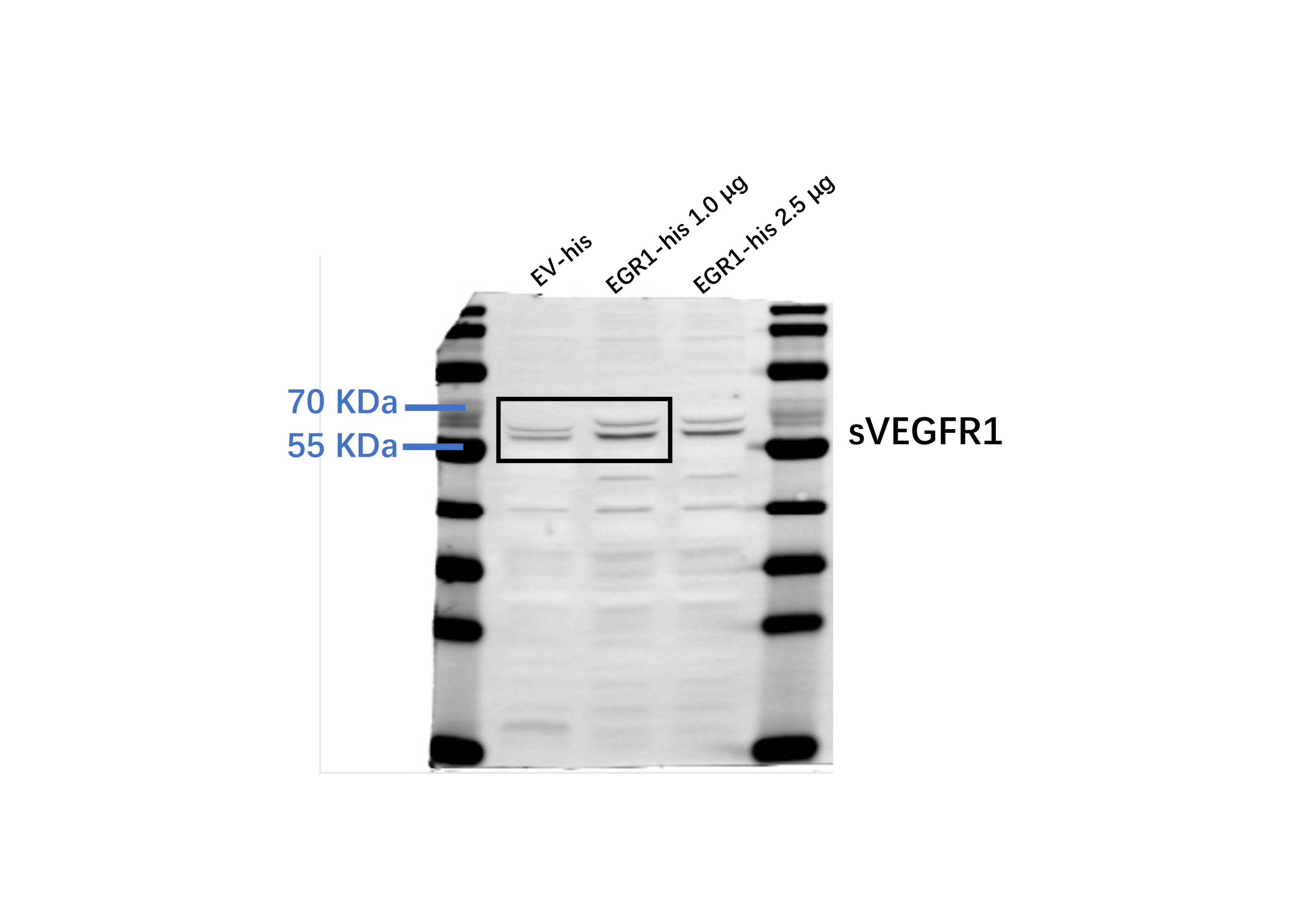

Supplement: Supplementary file 7 — Source data Fig. 5 [file 44319_2025_541_MOESM7_ESM.zip › Figure 5/5B/western sVEGFR1.tiff]

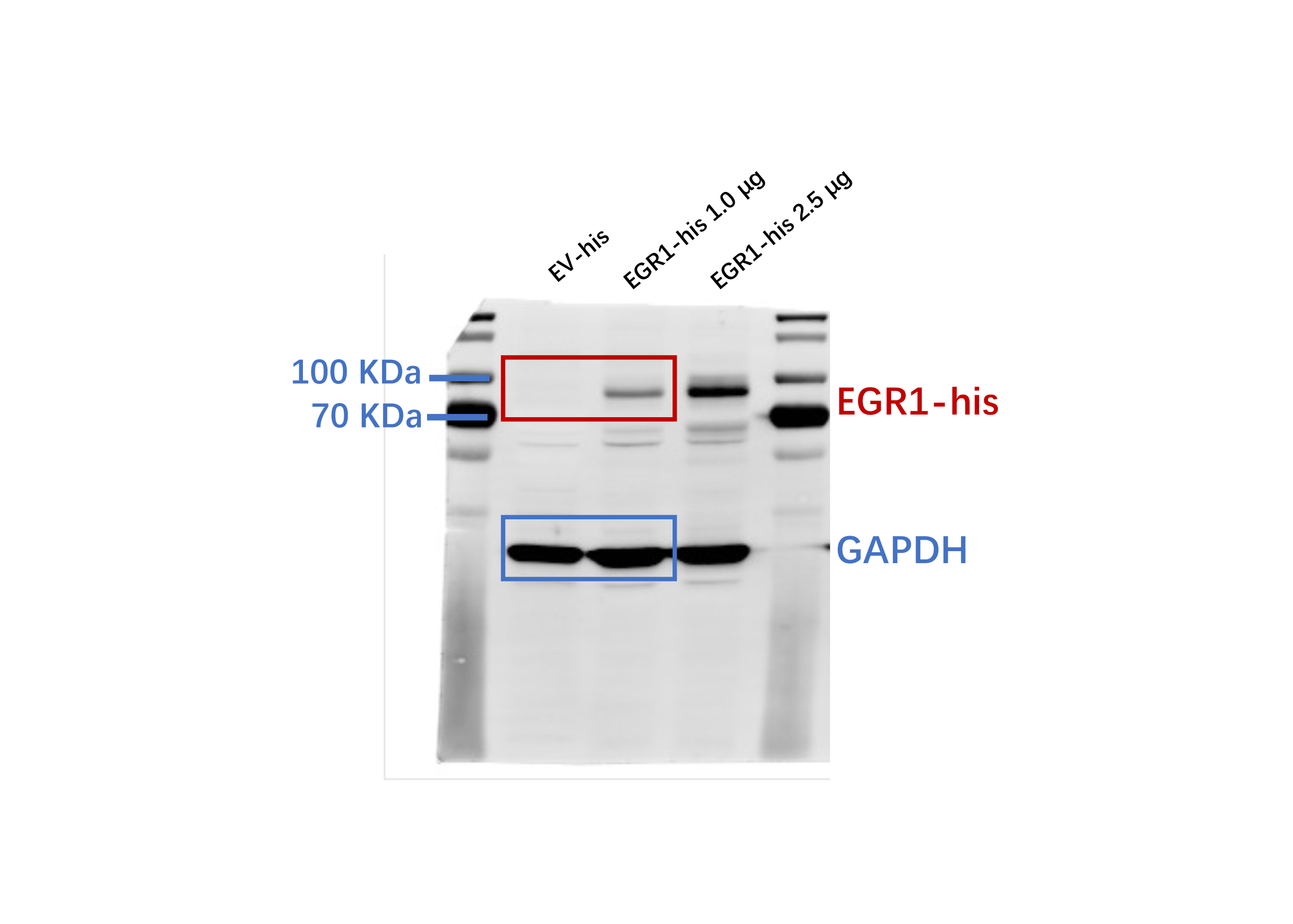

Supplement: Supplementary file 7 — Source data Fig. 5 [file 44319_2025_541_MOESM7_ESM.zip › Figure 5/5B/western His-tag and GAPDH.tiff]

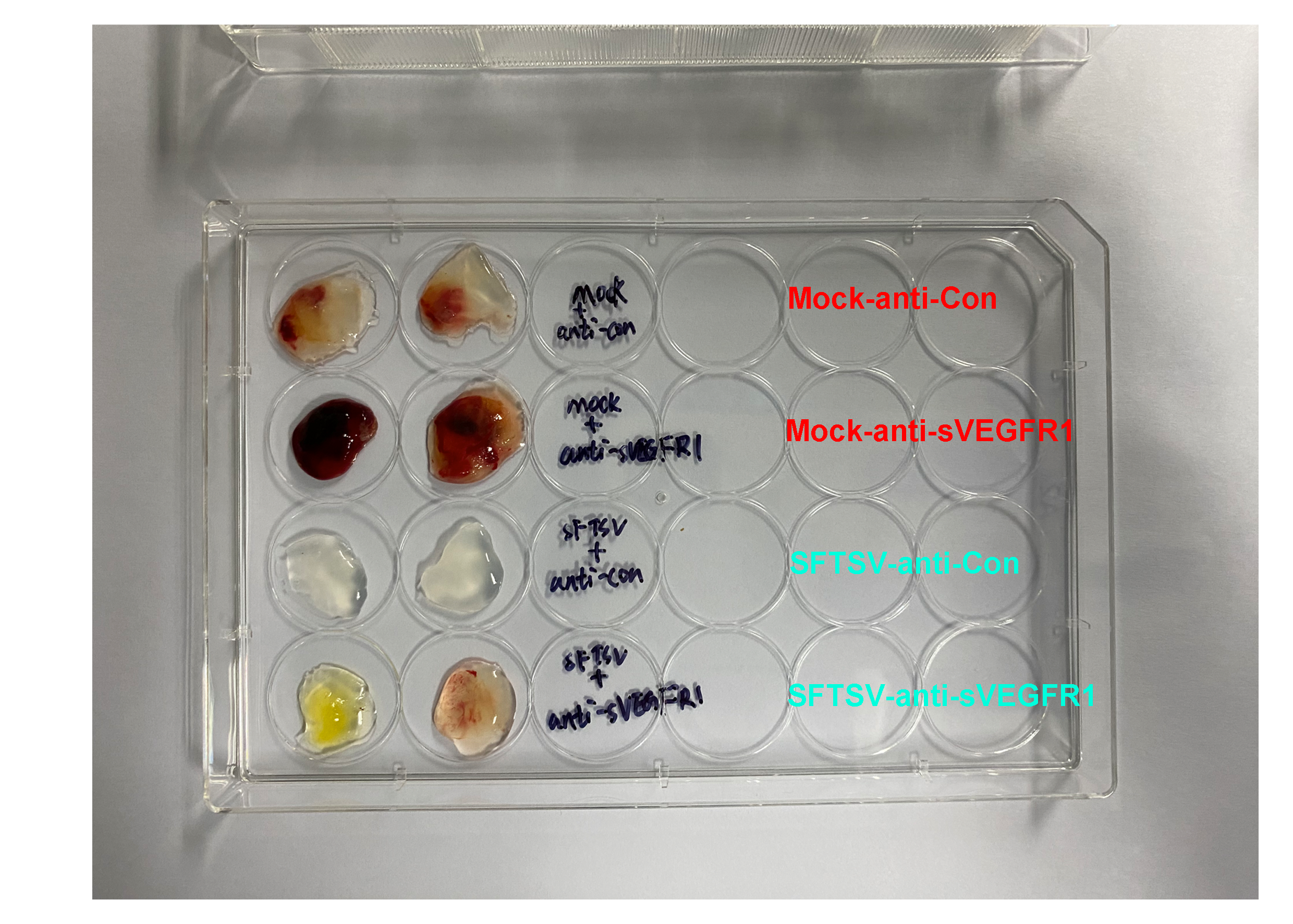

Supplement: Supplementary file 8 — Source data Fig. 6 [file 44319_2025_541_MOESM8_ESM.zip › Figure 6/6B/plugs from hu-PBL mice.tiff]

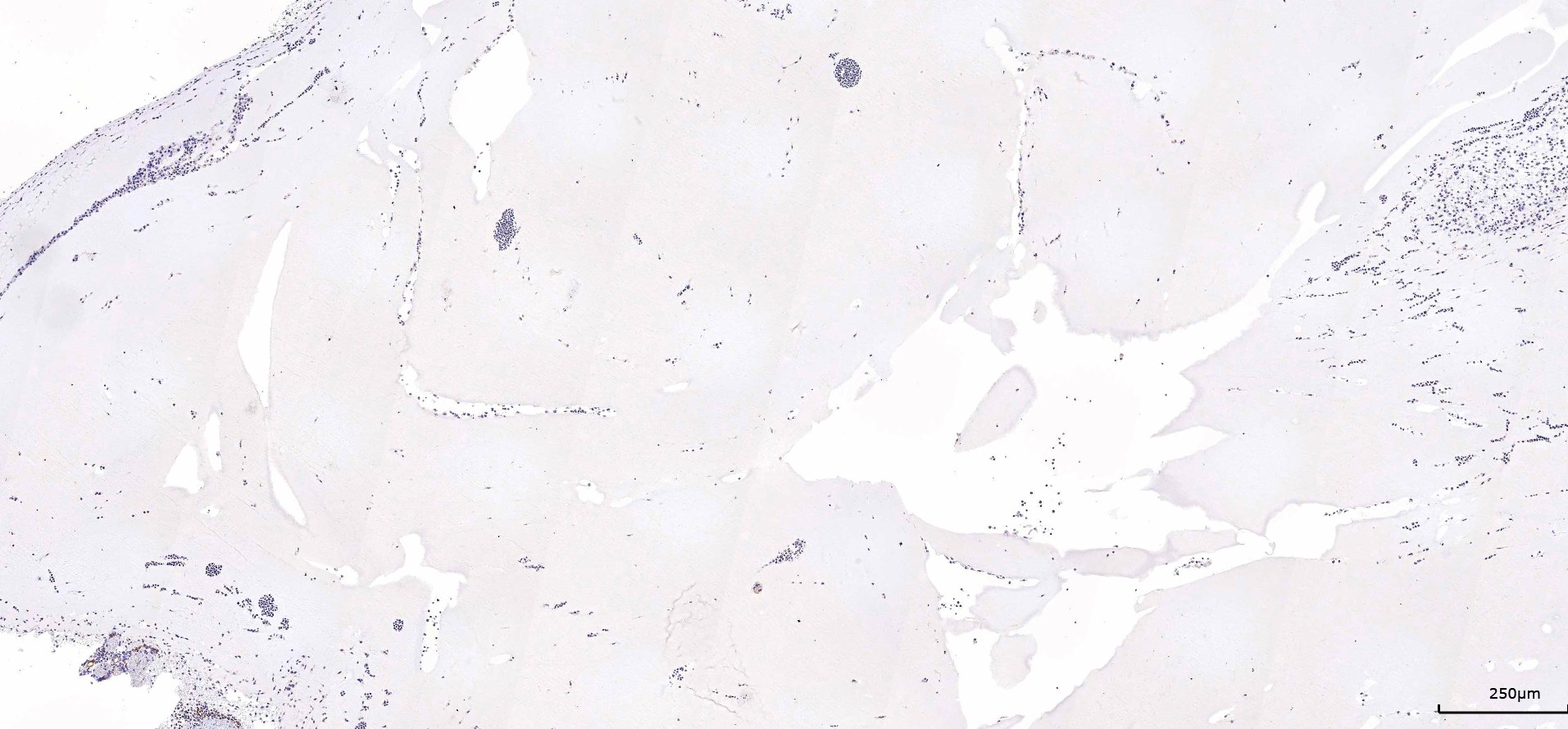

Supplement: Supplementary file 8 — Source data Fig. 6 [file 44319_2025_541_MOESM8_ESM.zip › Figure 6/6D/SFTSV_plug anti-sVEGFR1-CD31 4.00X.tiff]

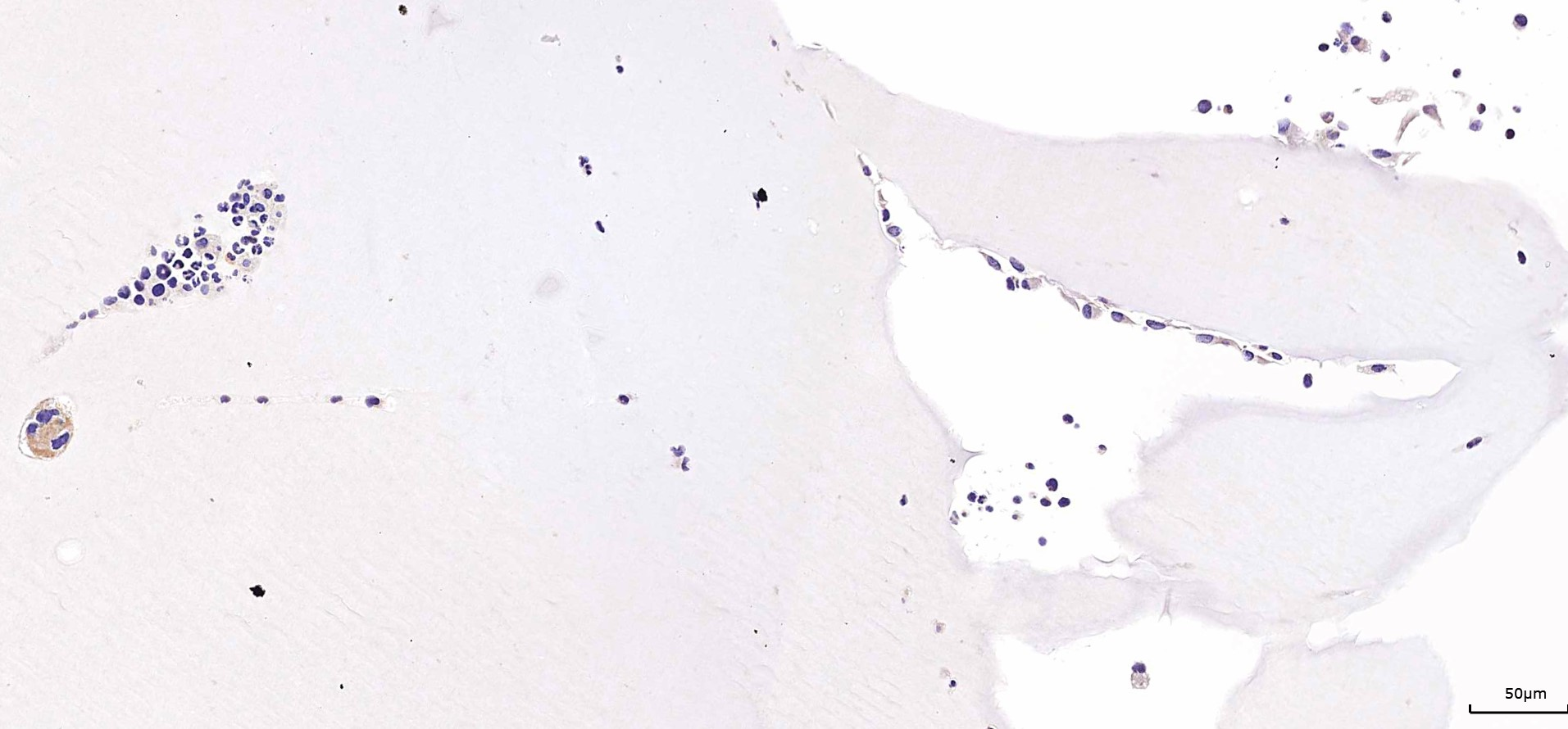

Supplement: Supplementary file 8 — Source data Fig. 6 [file 44319_2025_541_MOESM8_ESM.zip › Figure 6/6D/SFTSV_plug anti-sVEGFR1-CD31 20.00X.tiff]

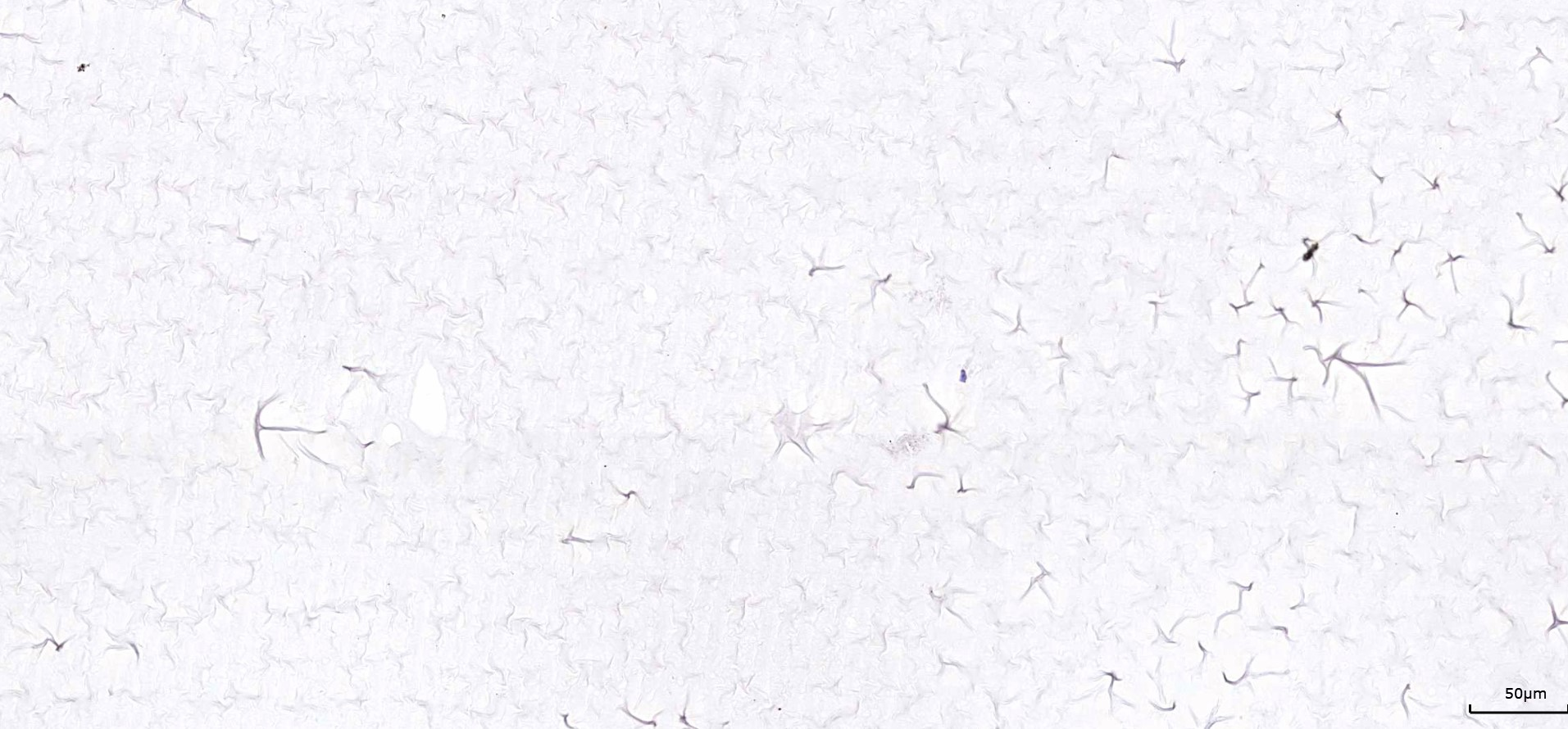

Supplement: Supplementary file 8 — Source data Fig. 6 [file 44319_2025_541_MOESM8_ESM.zip › Figure 6/6D/SFTSV_plug anti-Con-CD31 20.00X.tiff]

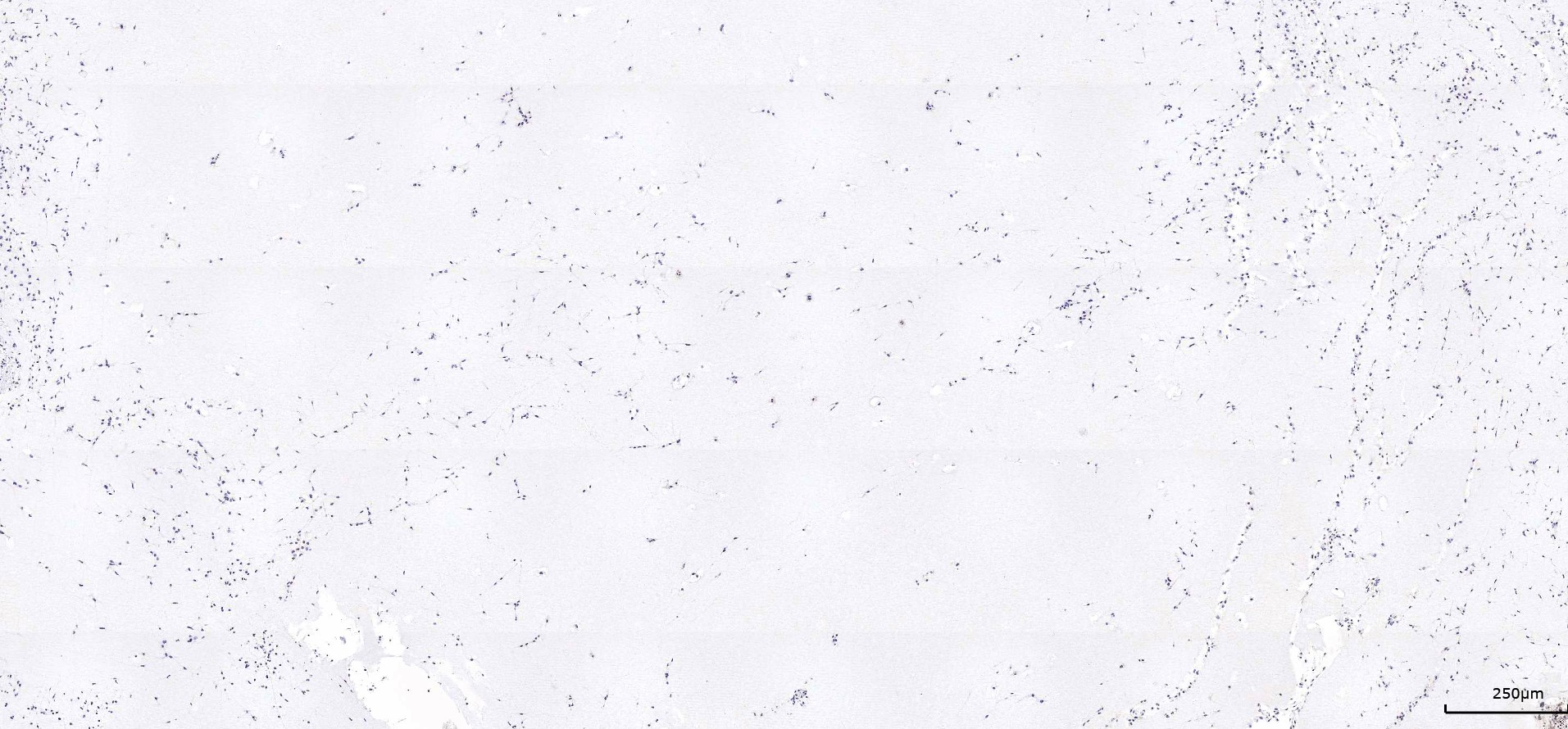

Supplement: Supplementary file 8 — Source data Fig. 6 [file 44319_2025_541_MOESM8_ESM.zip › Figure 6/6D/mock_plug anti-Con-CD31 4.00X.tiff]

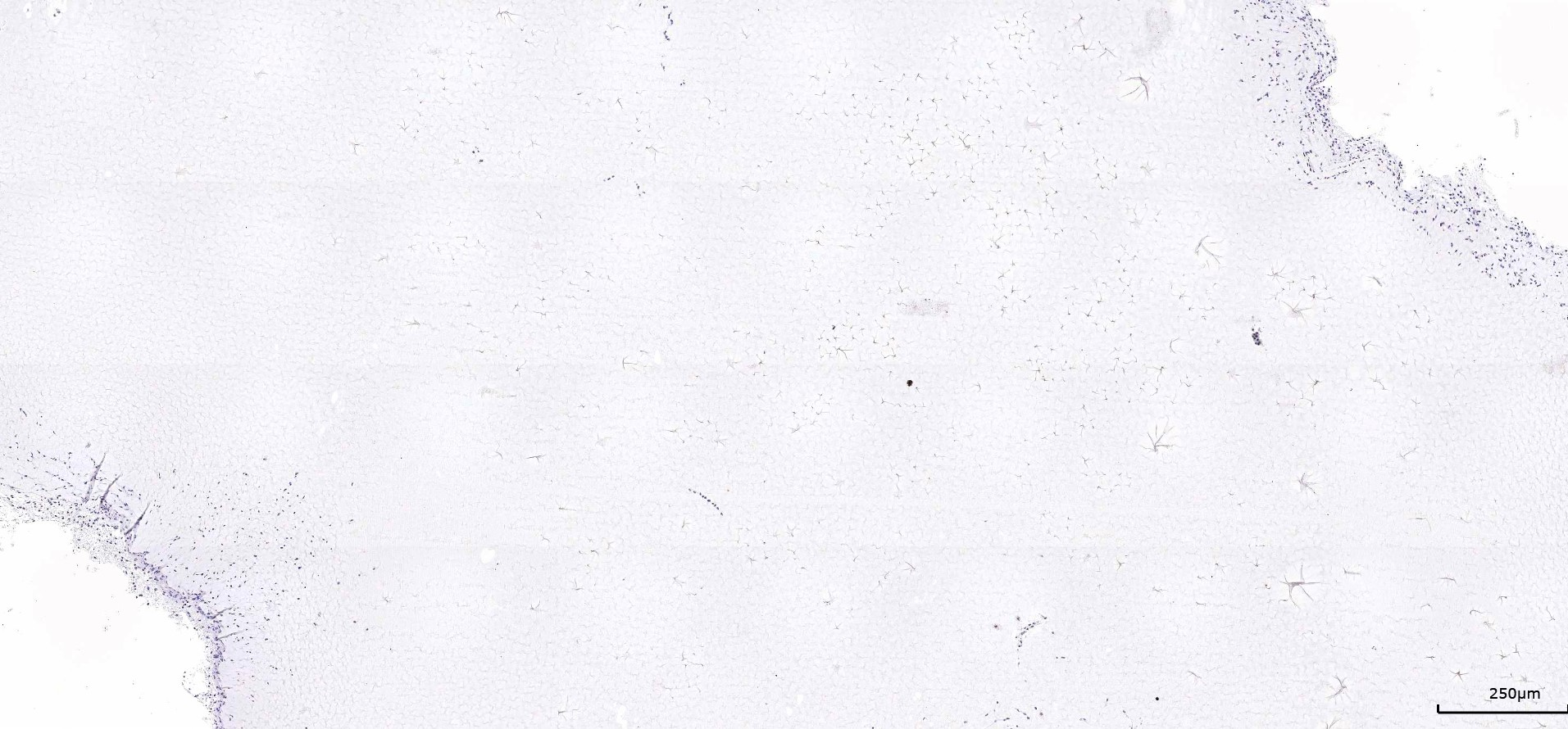

Supplement: Supplementary file 8 — Source data Fig. 6 [file 44319_2025_541_MOESM8_ESM.zip › Figure 6/6D/SFTSV_plug anti-Con-CD31 4.00X.tiff]

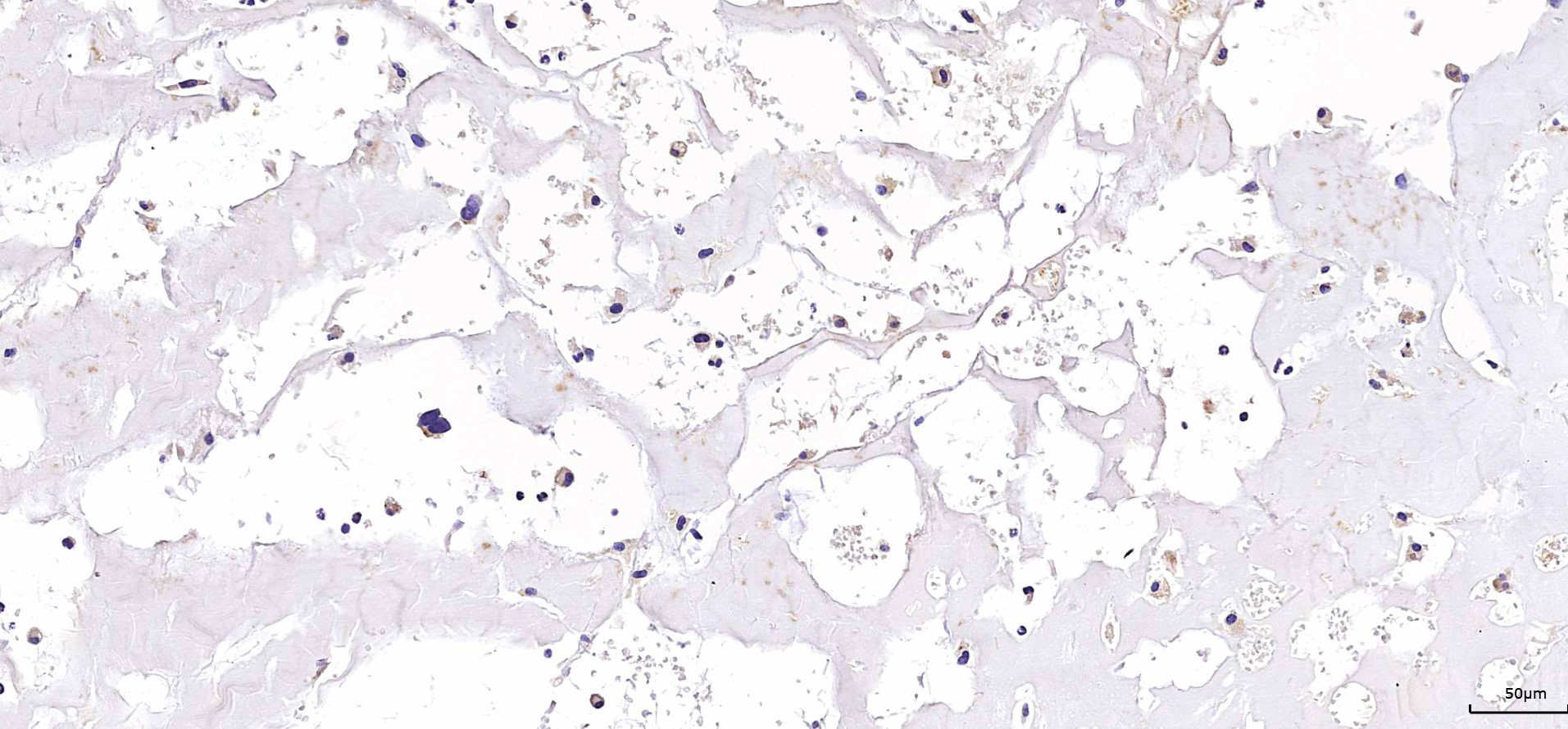

Supplement: Supplementary file 8 — Source data Fig. 6 [file 44319_2025_541_MOESM8_ESM.zip › Figure 6/6D/mock_plug anti-sVEGFR1-CD31 20.00X.tiff]

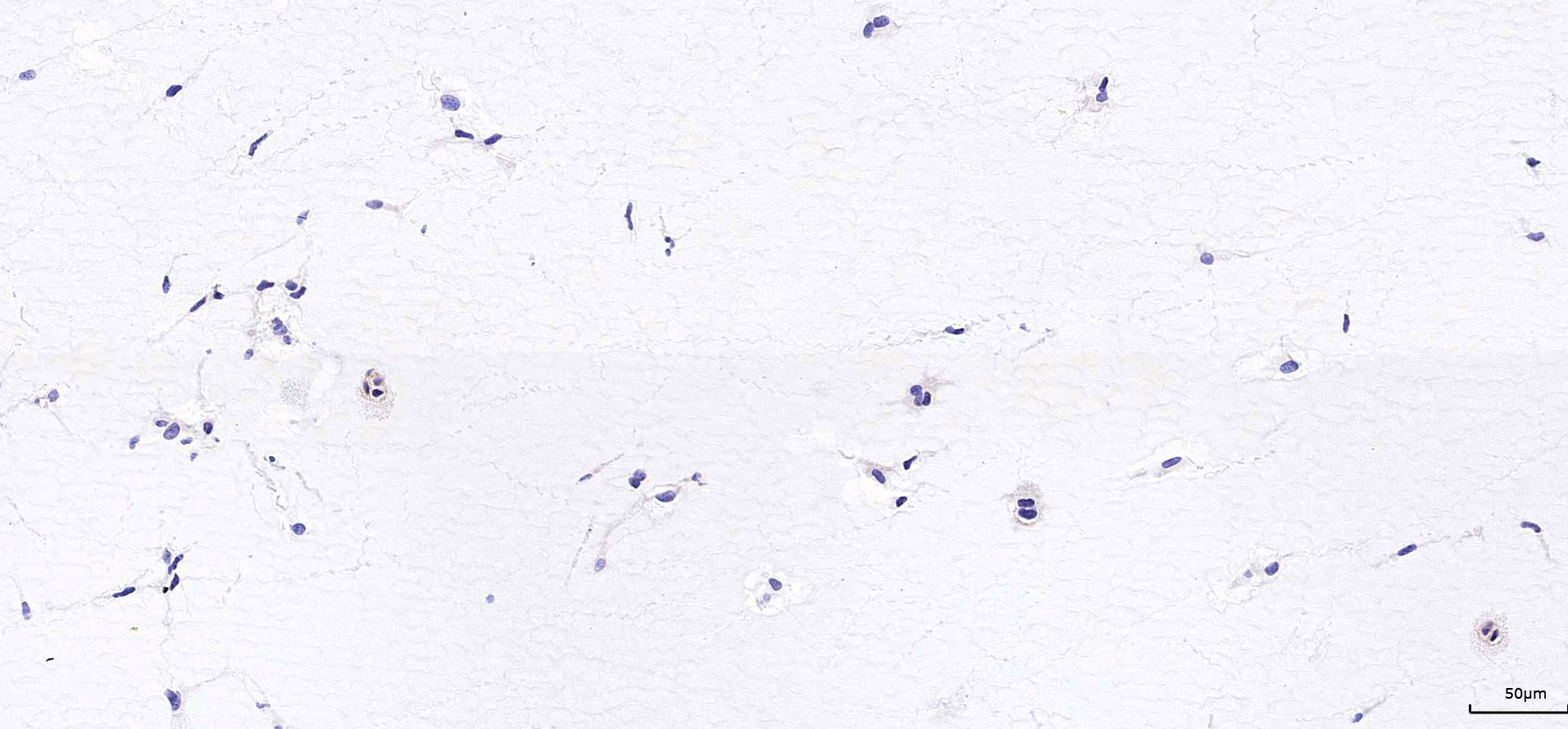

Supplement: Supplementary file 8 — Source data Fig. 6 [file 44319_2025_541_MOESM8_ESM.zip › Figure 6/6D/mock_plug anti-Con-CD31 20.00X.tiff]

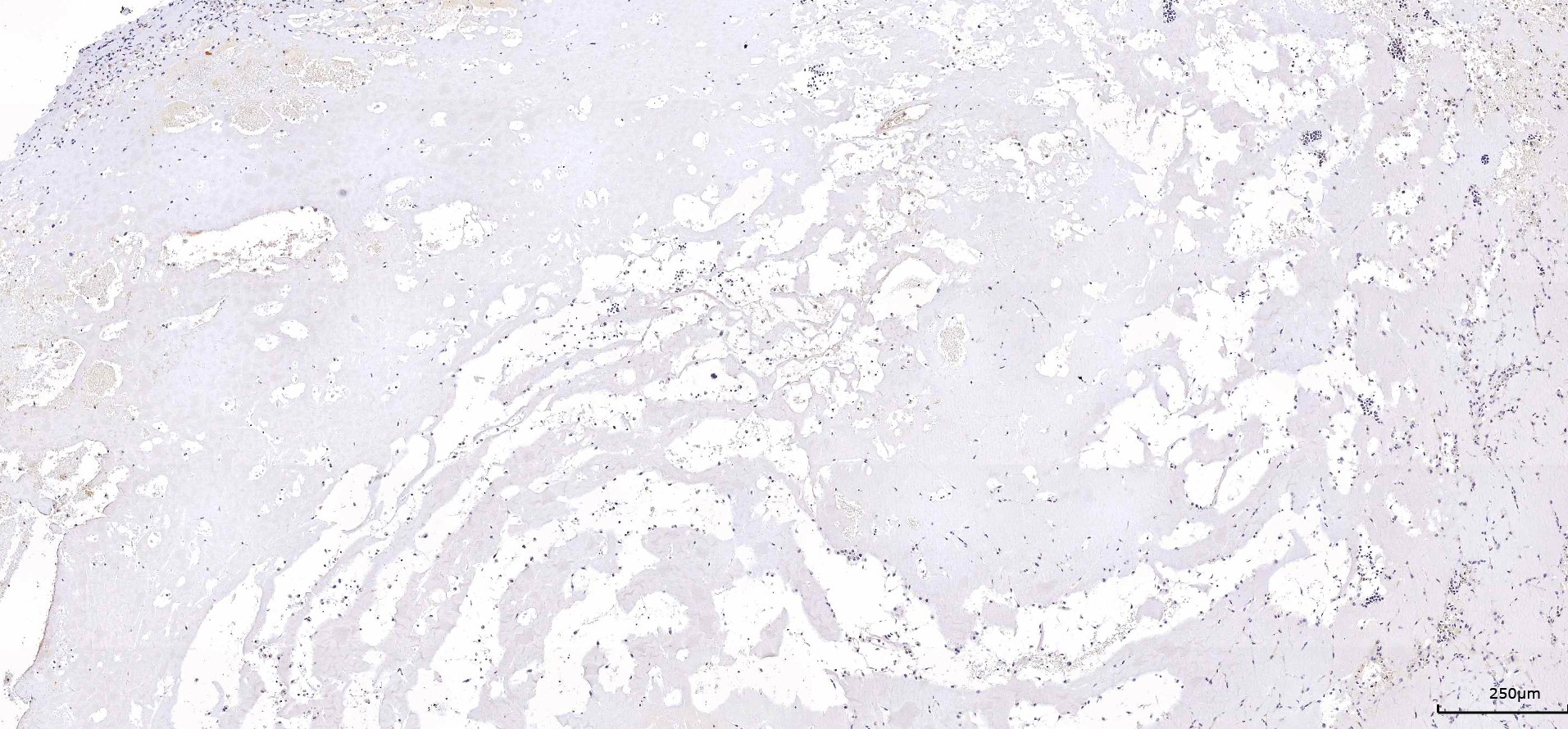

Supplement: Supplementary file 8 — Source data Fig. 6 [file 44319_2025_541_MOESM8_ESM.zip › Figure 6/6D/mock_plug anti-sVEGFR1-CD31 4.00X.tiff]

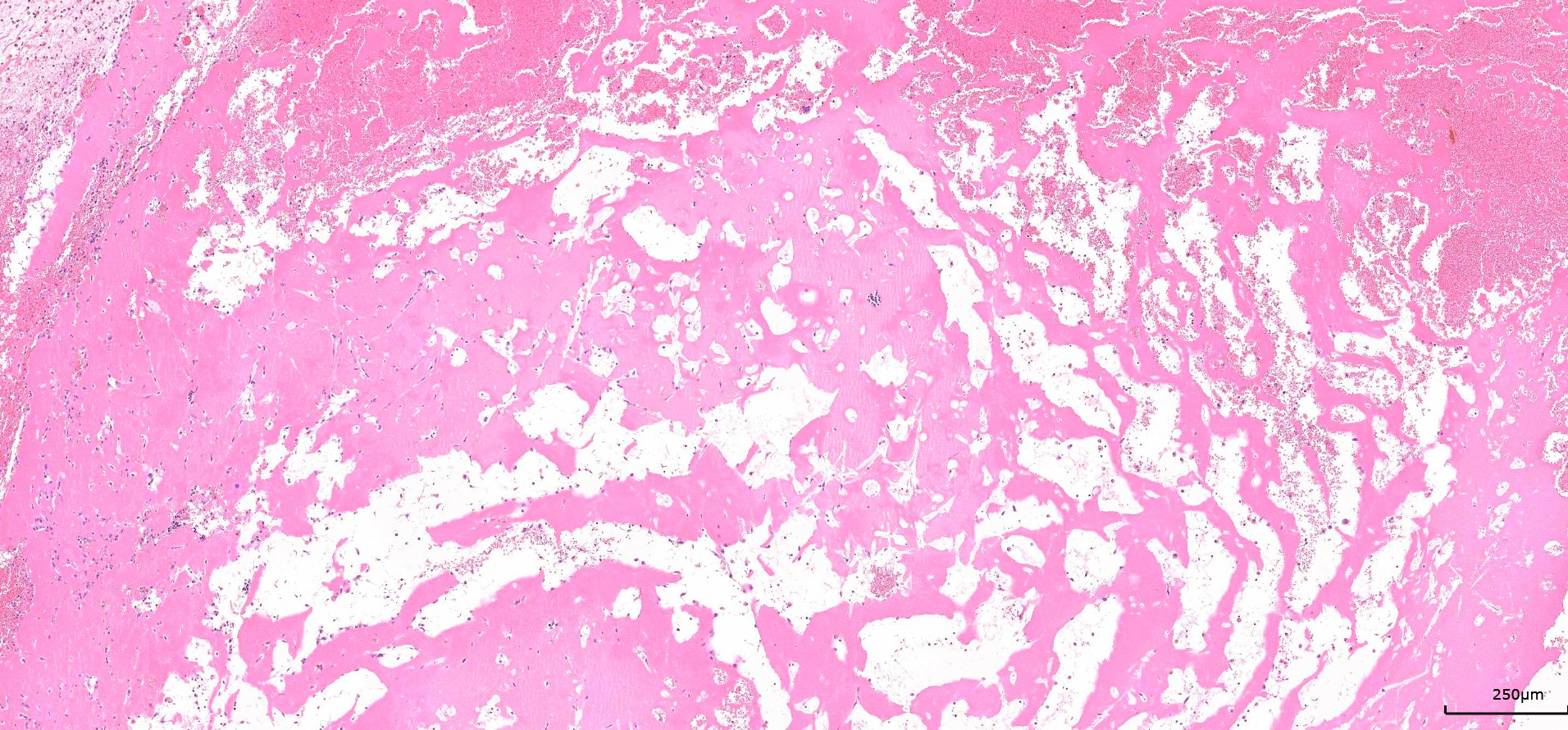

Supplement: Supplementary file 8 — Source data Fig. 6 [file 44319_2025_541_MOESM8_ESM.zip › Figure 6/6C/mock_plug anti-sVEGFR1 4X.tiff]

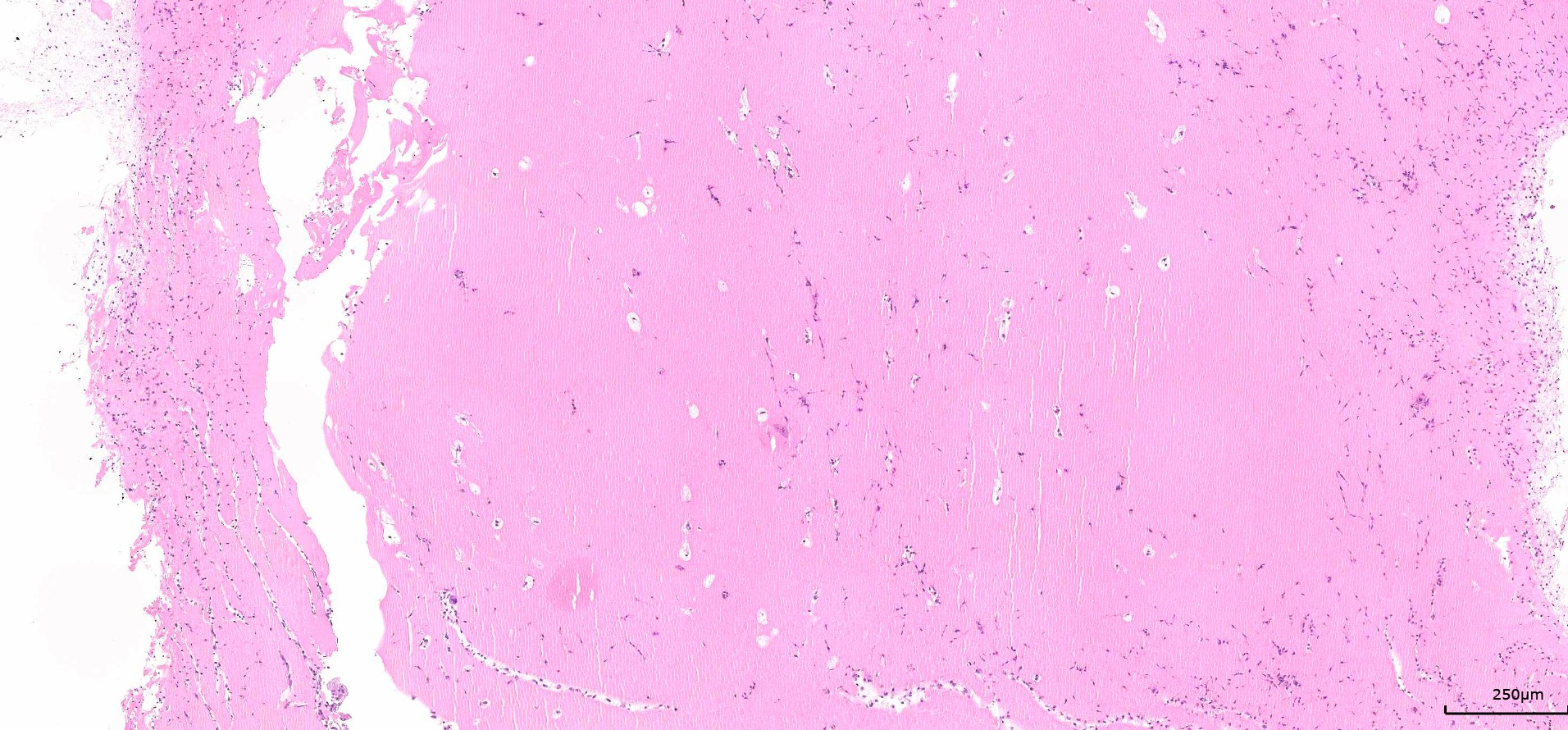

Supplement: Supplementary file 8 — Source data Fig. 6 [file 44319_2025_541_MOESM8_ESM.zip › Figure 6/6C/mock_plug anti-Con 4X.tiff]

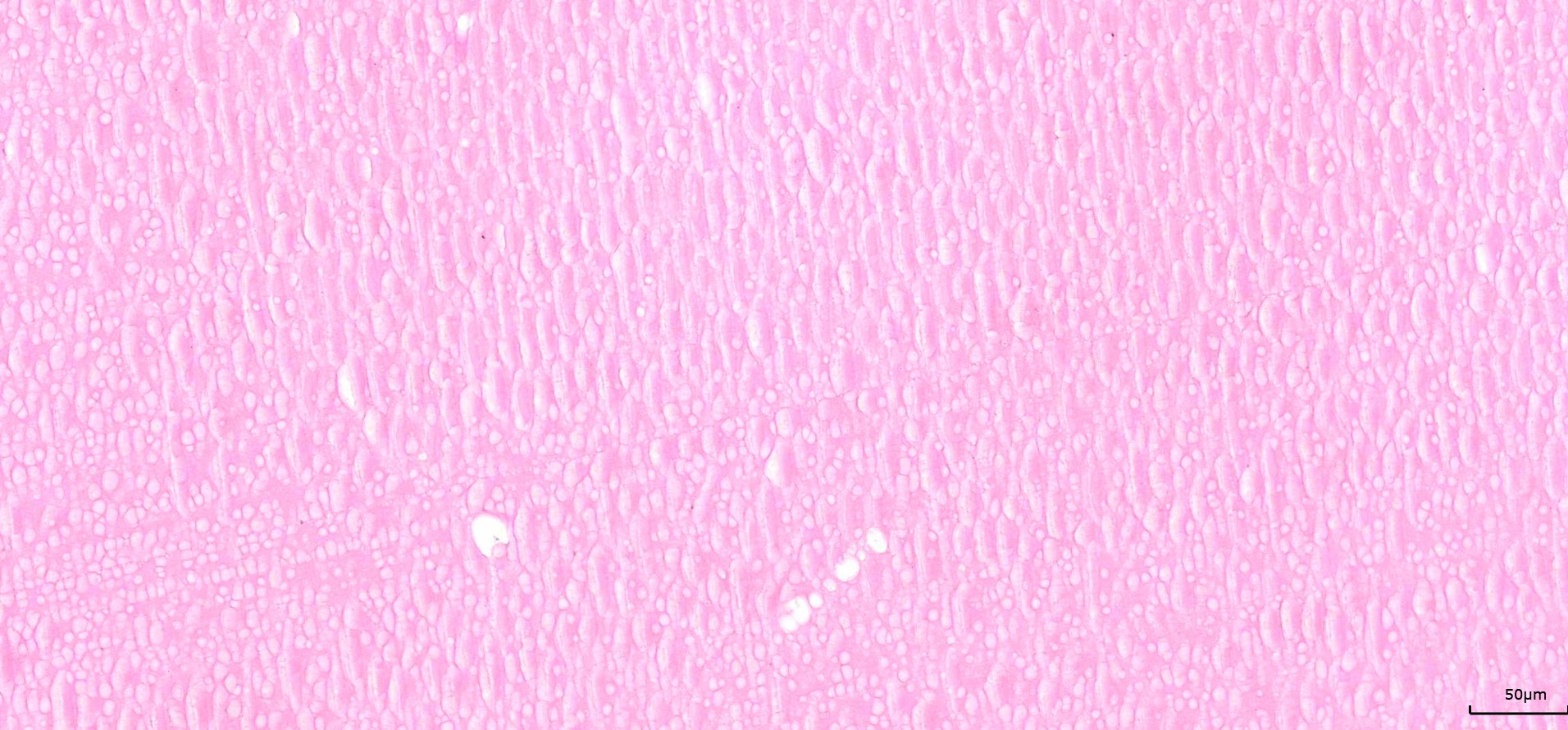

Supplement: Supplementary file 8 — Source data Fig. 6 [file 44319_2025_541_MOESM8_ESM.zip › Figure 6/6C/SFTSV_plug anti-Con 20.00X.tiff]

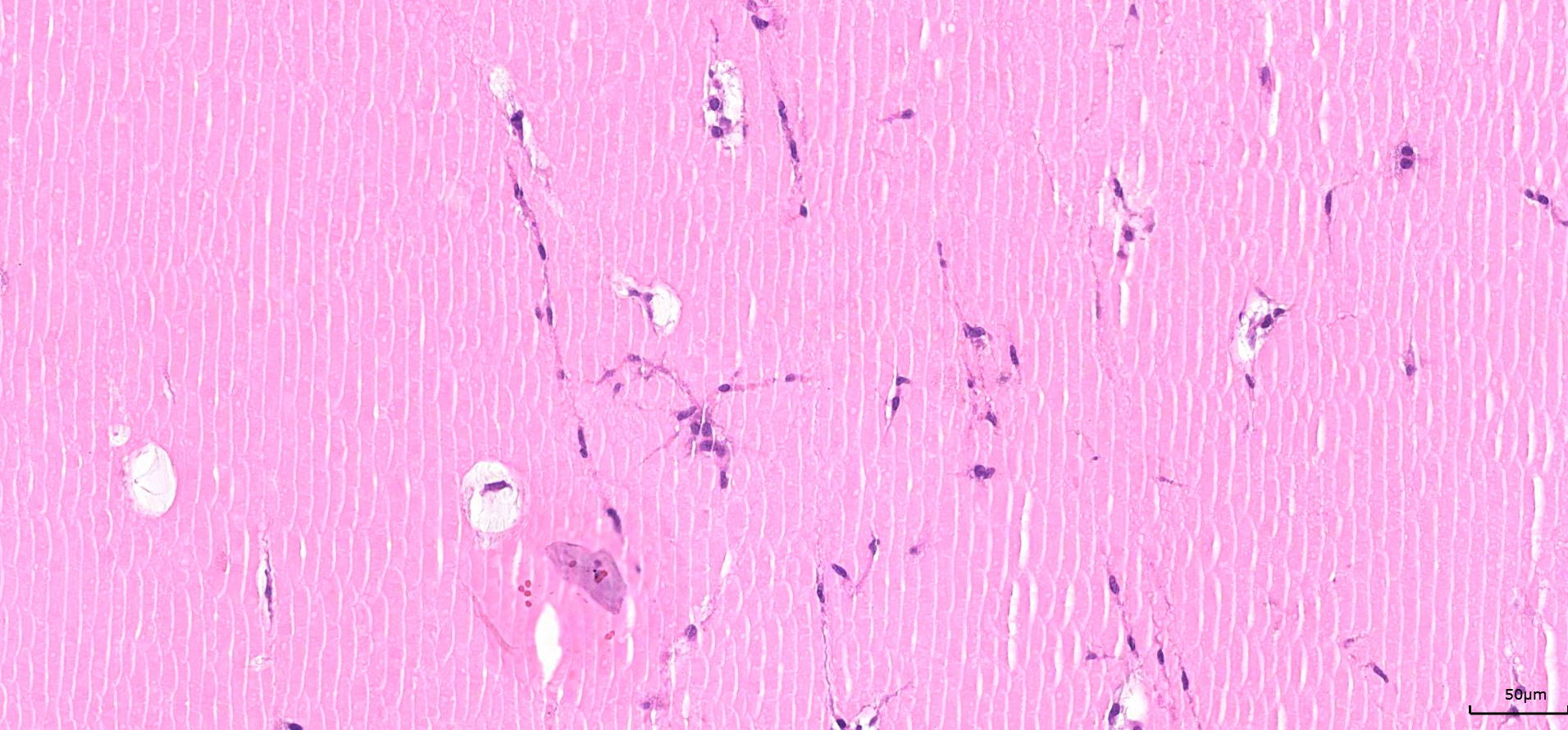

Supplement: Supplementary file 8 — Source data Fig. 6 [file 44319_2025_541_MOESM8_ESM.zip › Figure 6/6C/mock_plug anti-Con 20.00X.tiff]

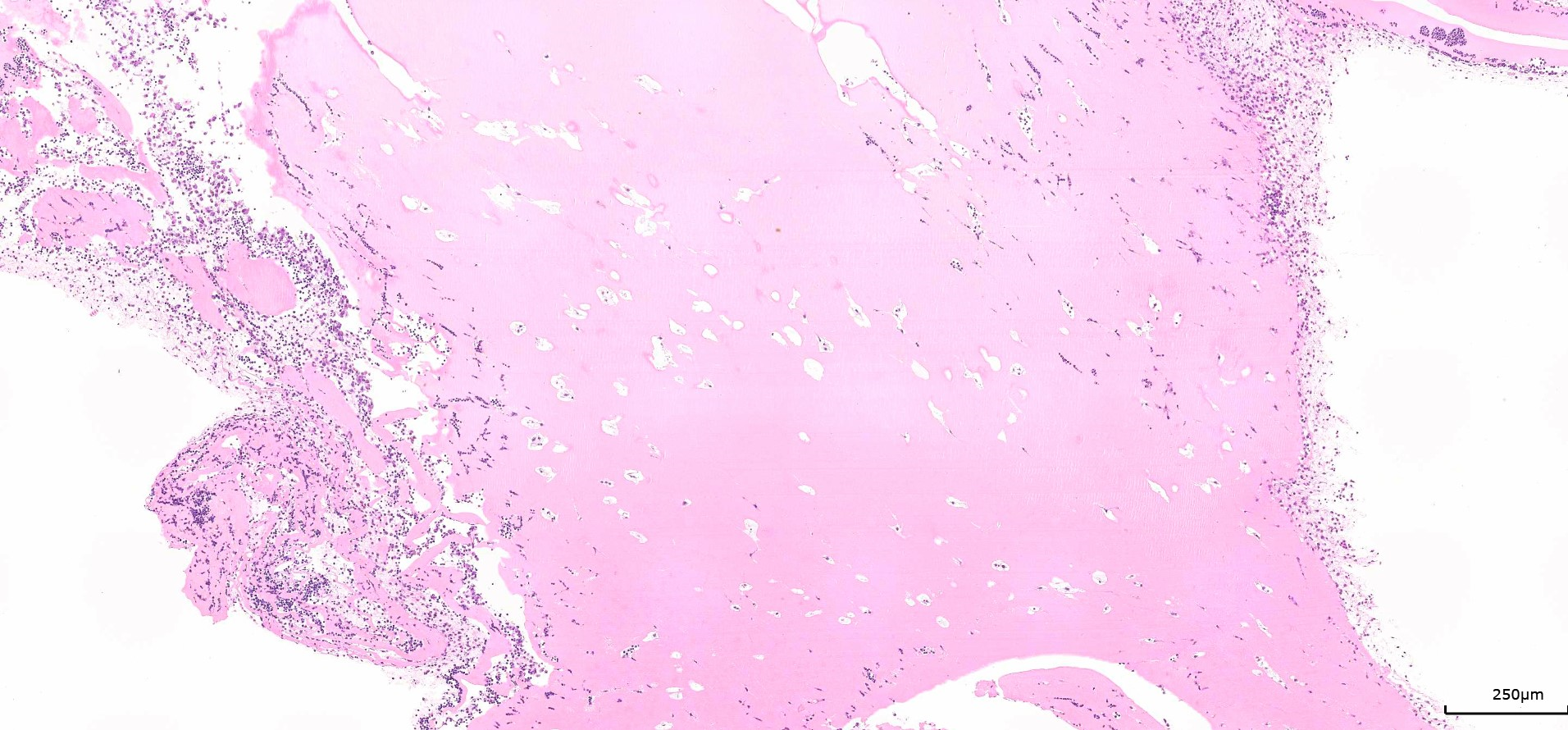

Supplement: Supplementary file 8 — Source data Fig. 6 [file 44319_2025_541_MOESM8_ESM.zip › Figure 6/6C/SFTSV_plug anti-sVEGFR1 4X.tiff]

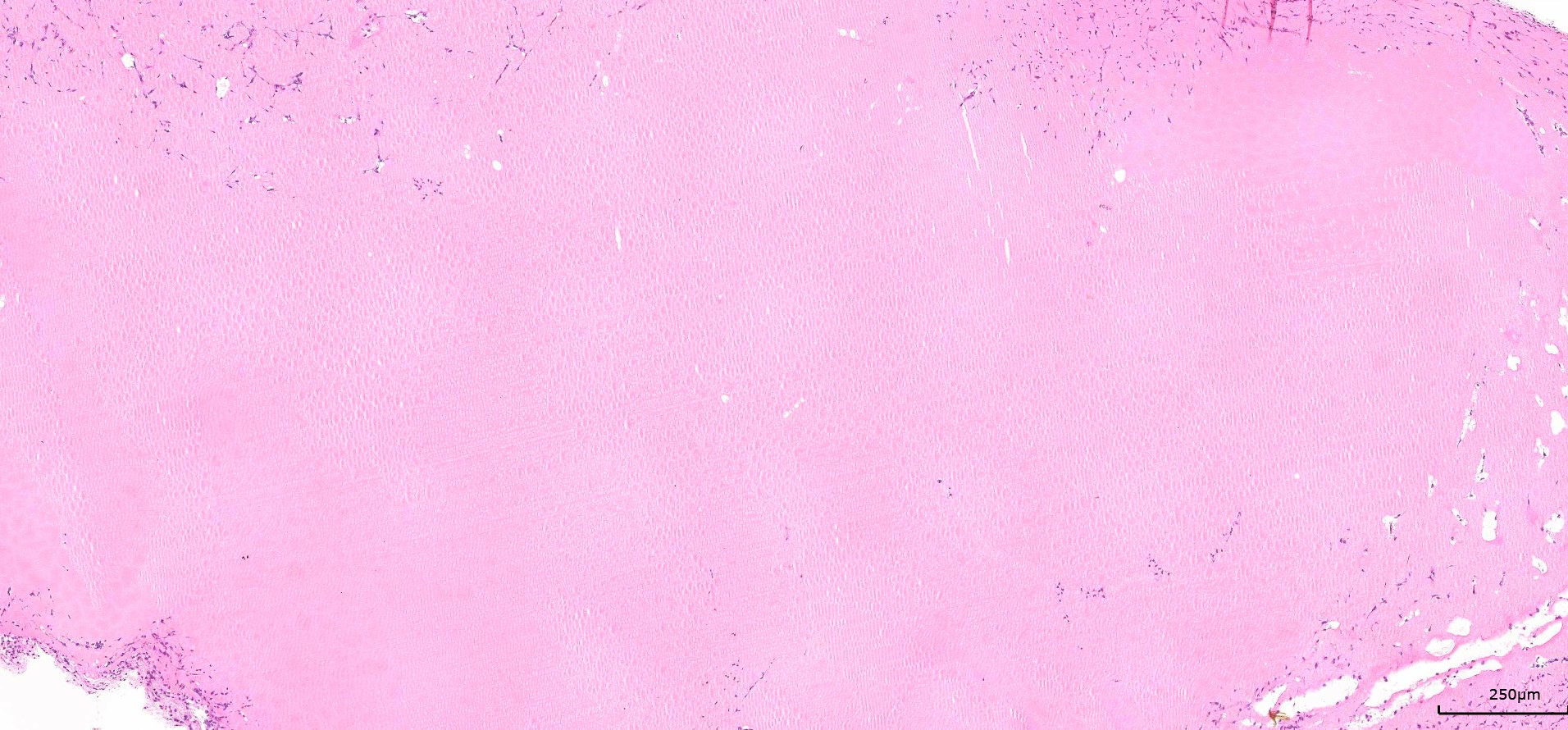

Supplement: Supplementary file 8 — Source data Fig. 6 [file 44319_2025_541_MOESM8_ESM.zip › Figure 6/6C/SFTSV_plug anti-Con 4X.tiff]

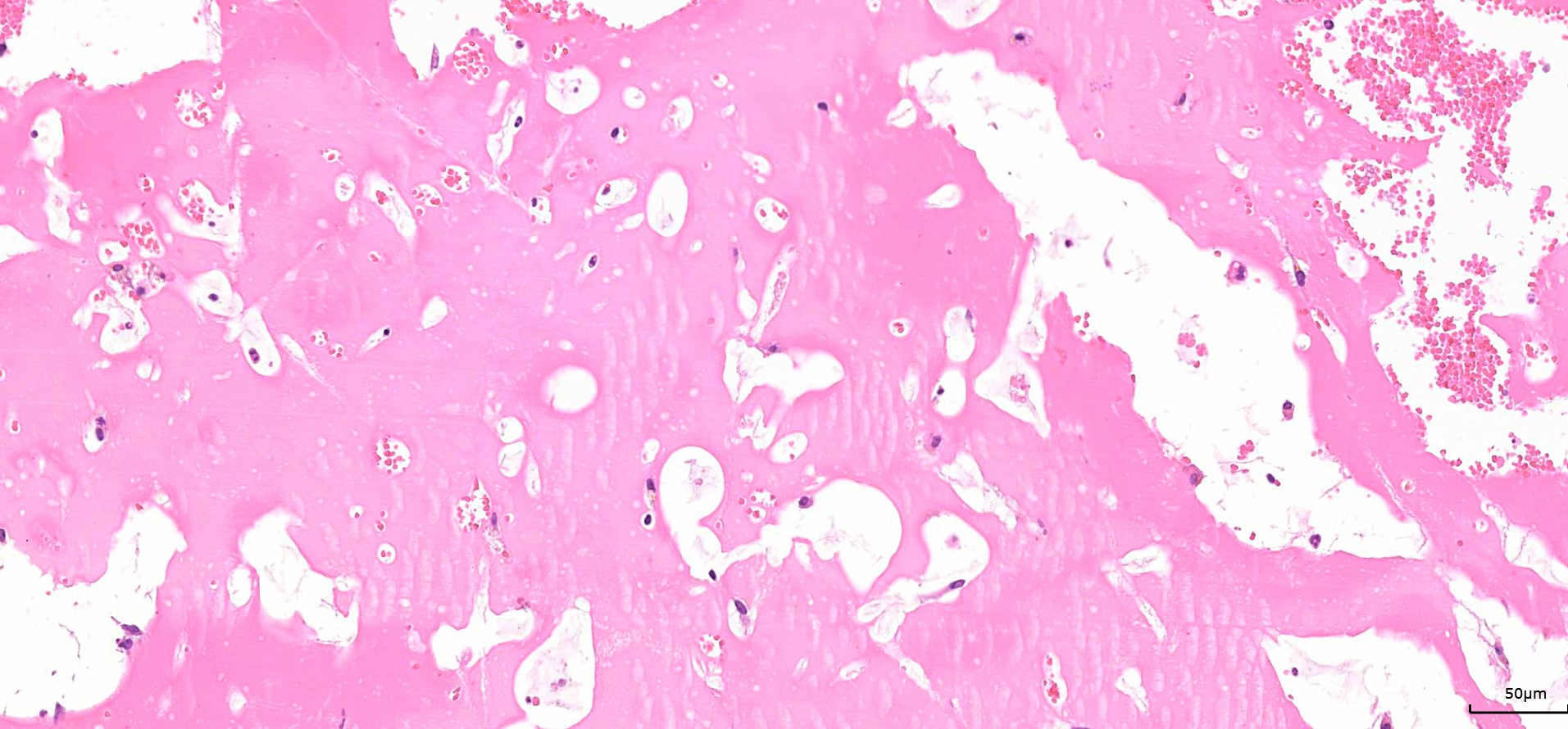

Supplement: Supplementary file 8 — Source data Fig. 6 [file 44319_2025_541_MOESM8_ESM.zip › Figure 6/6C/mock_plug anti-sVEGFR1 20.00X.tiff]

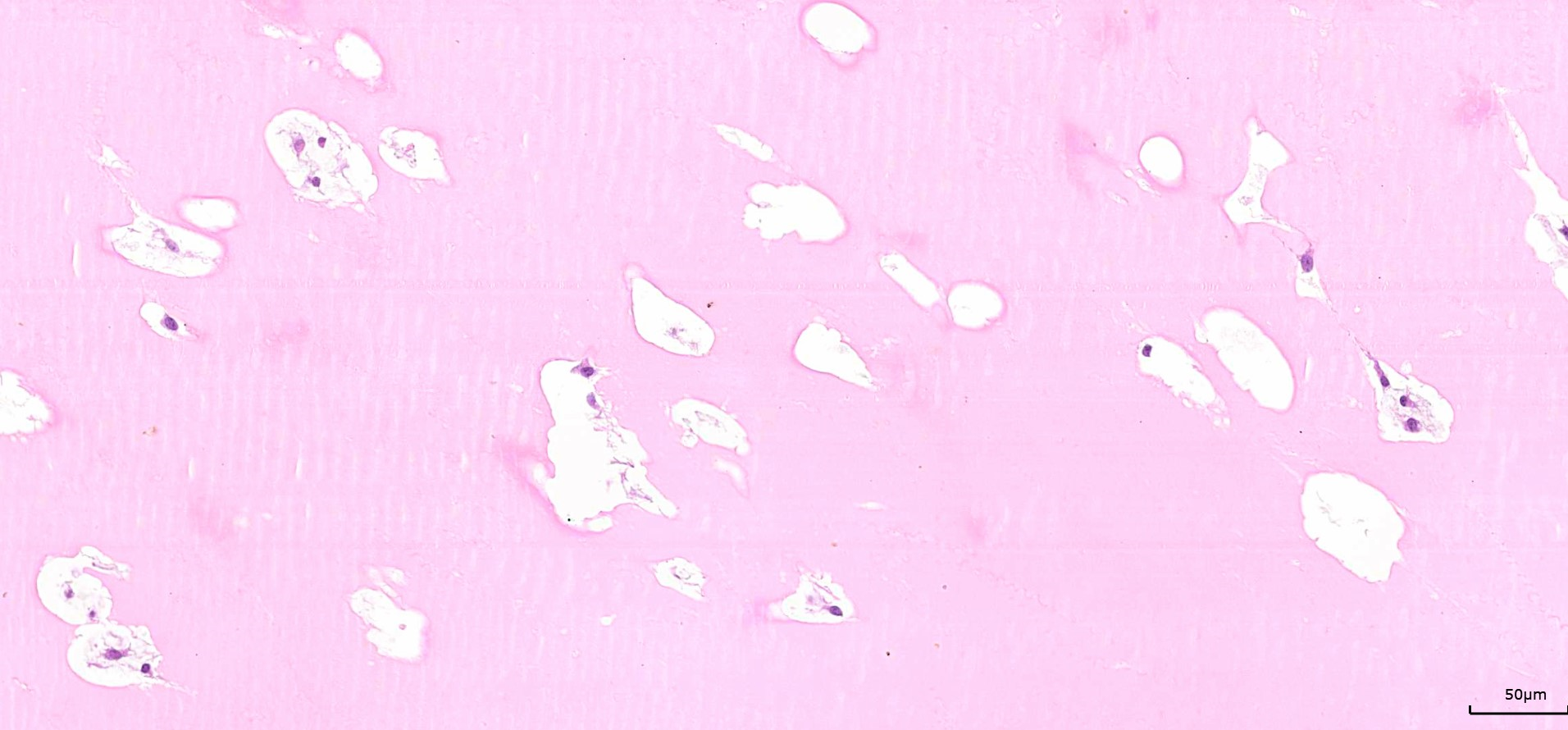

Supplement: Supplementary file 8 — Source data Fig. 6 [file 44319_2025_541_MOESM8_ESM.zip › Figure 6/6C/SFTSV_plug anti-sVEGFR1 20.00X.tiff]

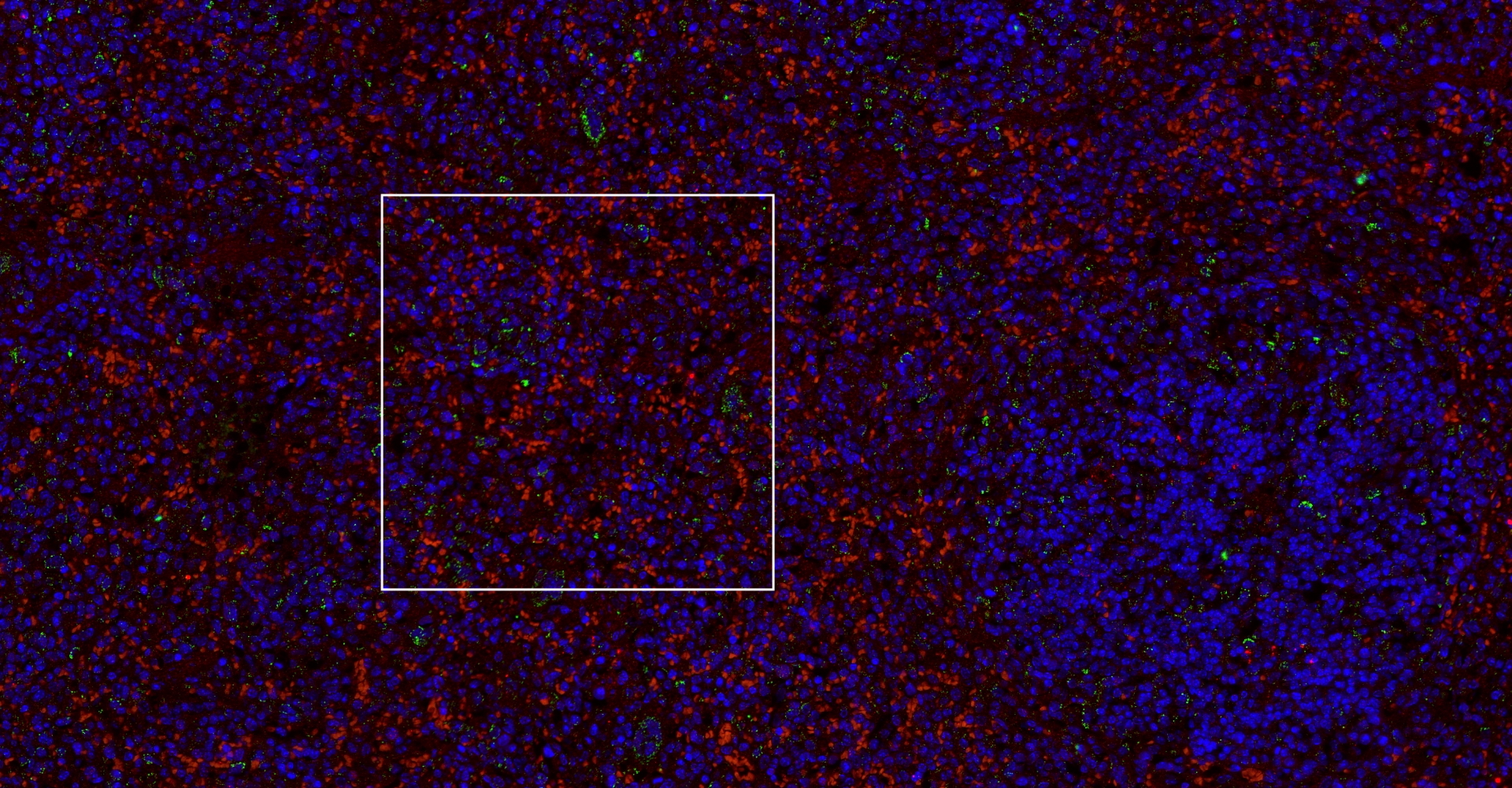

Supplement: Supplementary file 9 — Source data Fig. 7 [file 44319_2025_541_MOESM9_ESM.zip › Figure 7/7J/WT_spleen merge 20.0x.tiff]

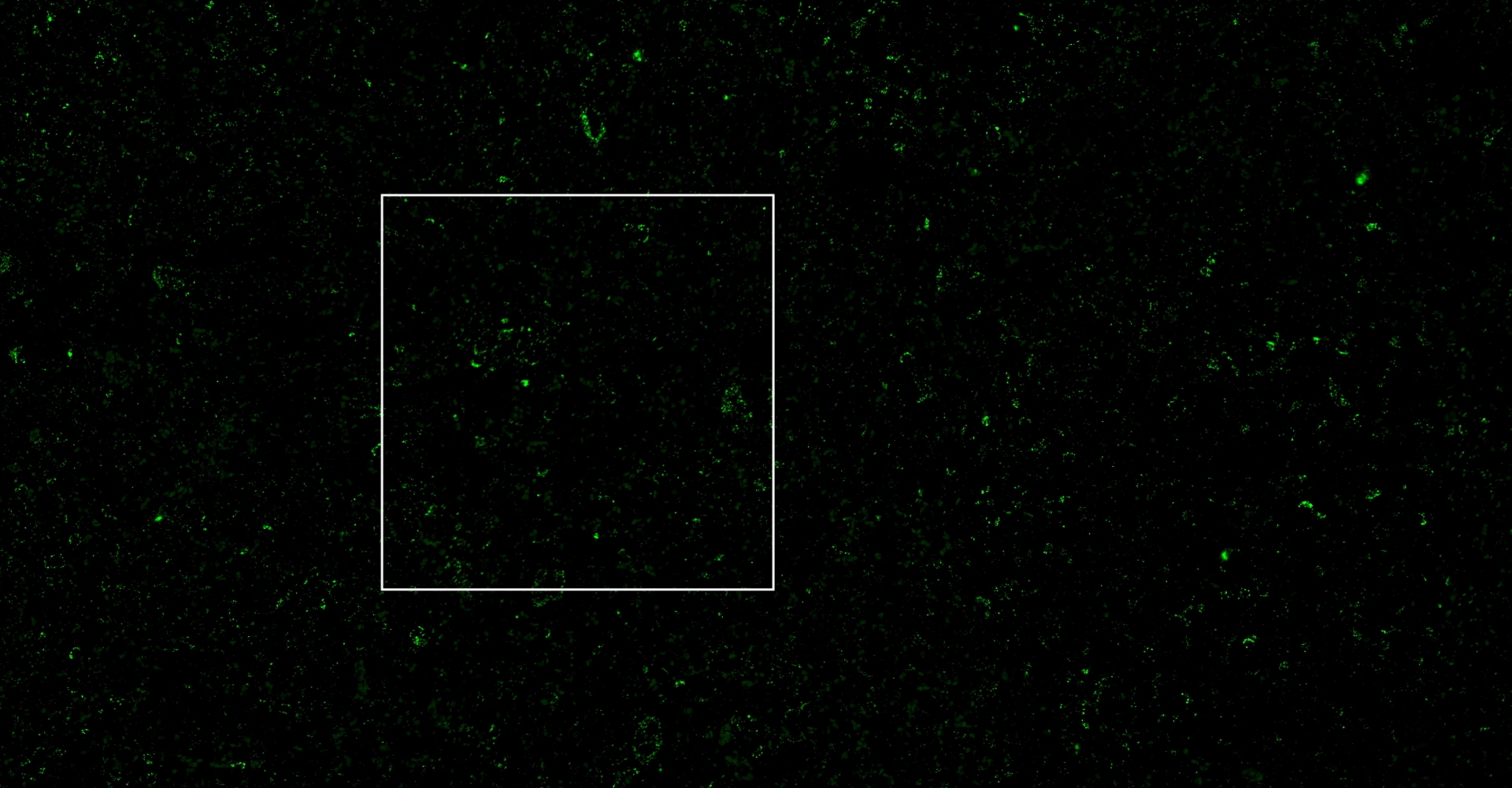

Supplement: Supplementary file 9 — Source data Fig. 7 [file 44319_2025_541_MOESM9_ESM.zip › Figure 7/7J/WT_spleen Gn 20.0x.tiff]

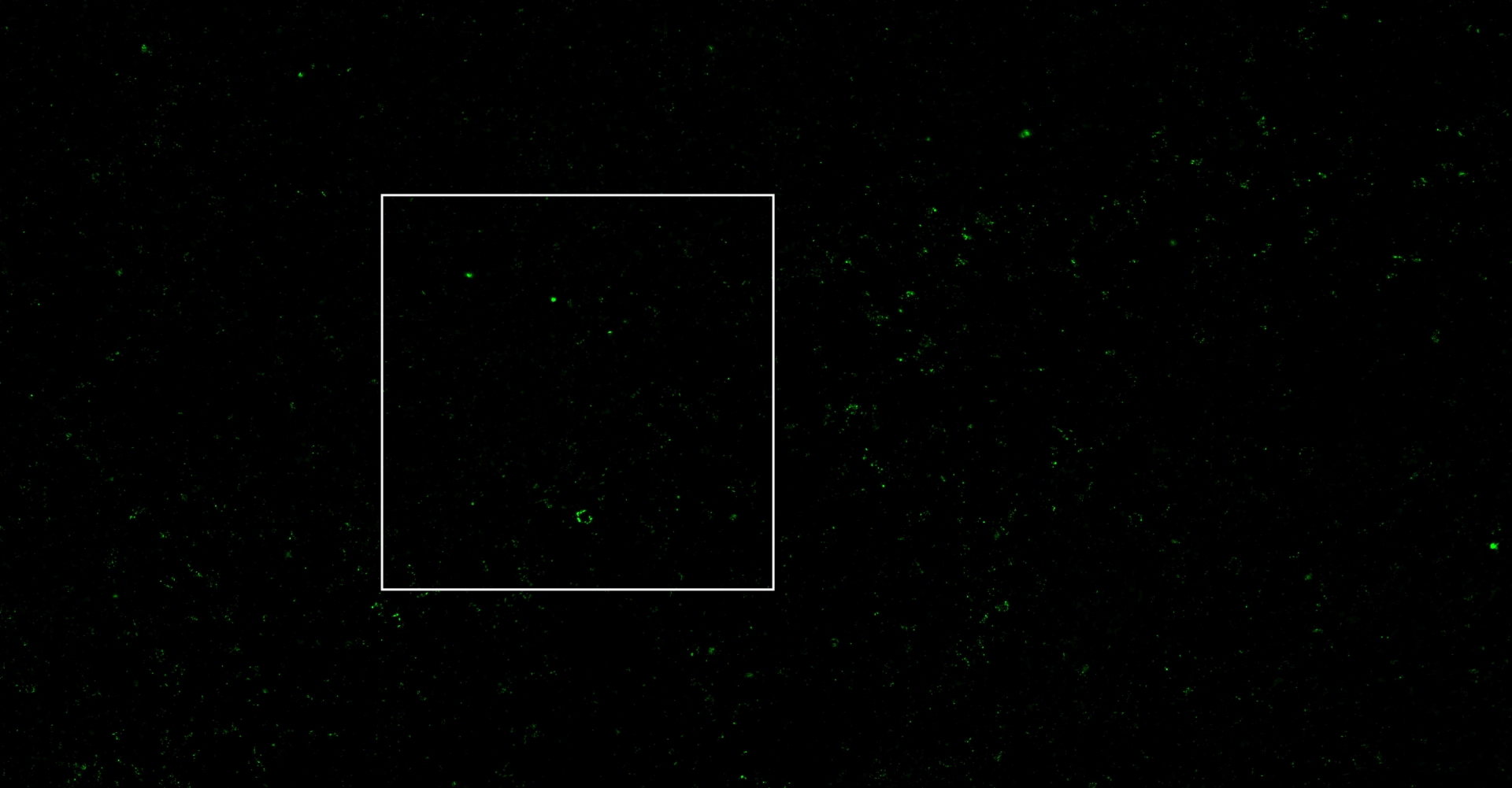

Supplement: Supplementary file 9 — Source data Fig. 7 [file 44319_2025_541_MOESM9_ESM.zip › Figure 7/7J/KO_spleen Gn 20.0x.tiff]

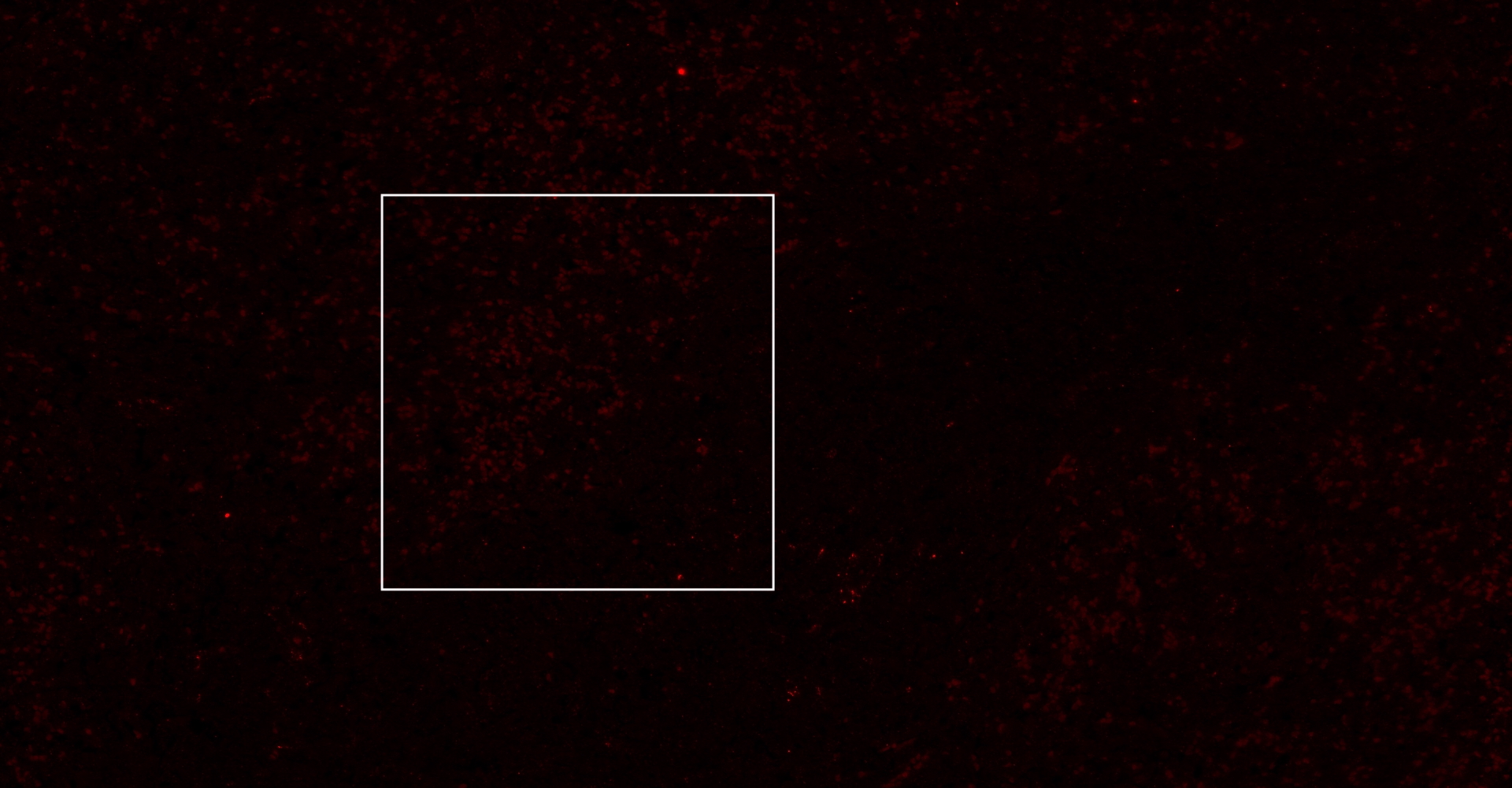

Supplement: Supplementary file 9 — Source data Fig. 7 [file 44319_2025_541_MOESM9_ESM.zip › Figure 7/7J/KO_spleen sVEGFR1 20.0x.tiff]

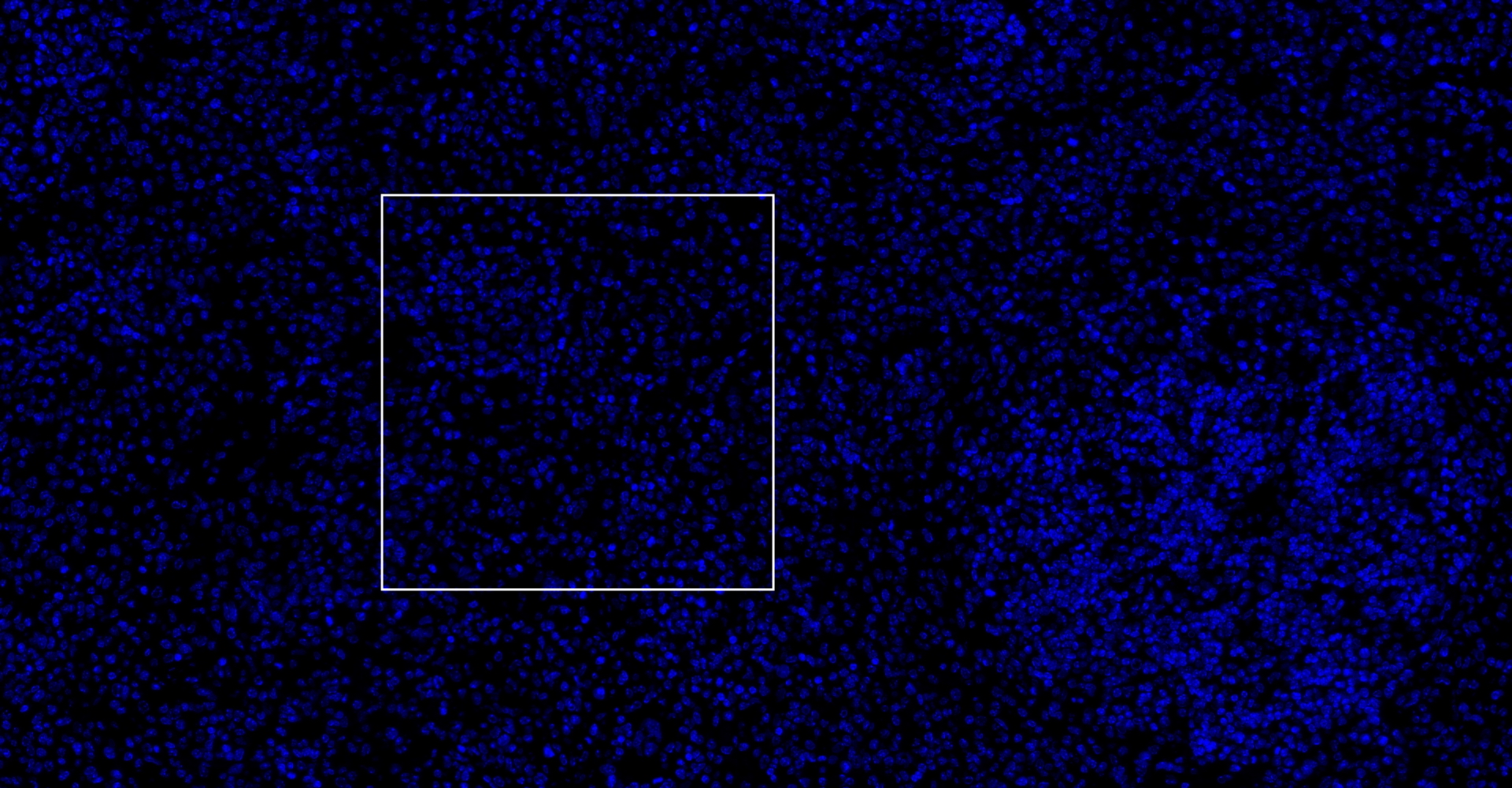

Supplement: Supplementary file 9 — Source data Fig. 7 [file 44319_2025_541_MOESM9_ESM.zip › Figure 7/7J/WT_spleen dapi 20.0x.tiff]

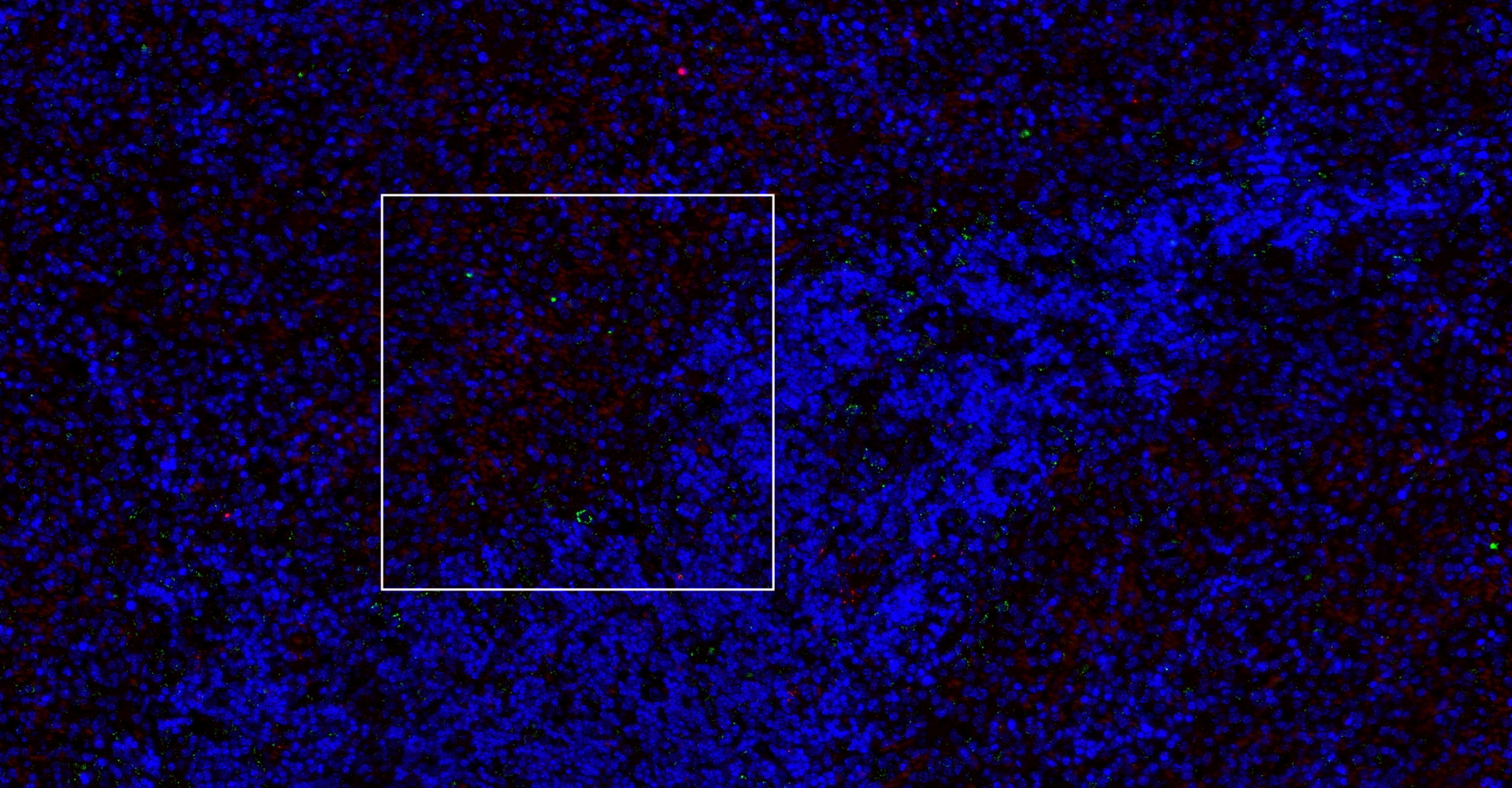

Supplement: Supplementary file 9 — Source data Fig. 7 [file 44319_2025_541_MOESM9_ESM.zip › Figure 7/7J/KO_spleen merge 20.0x.tiff]

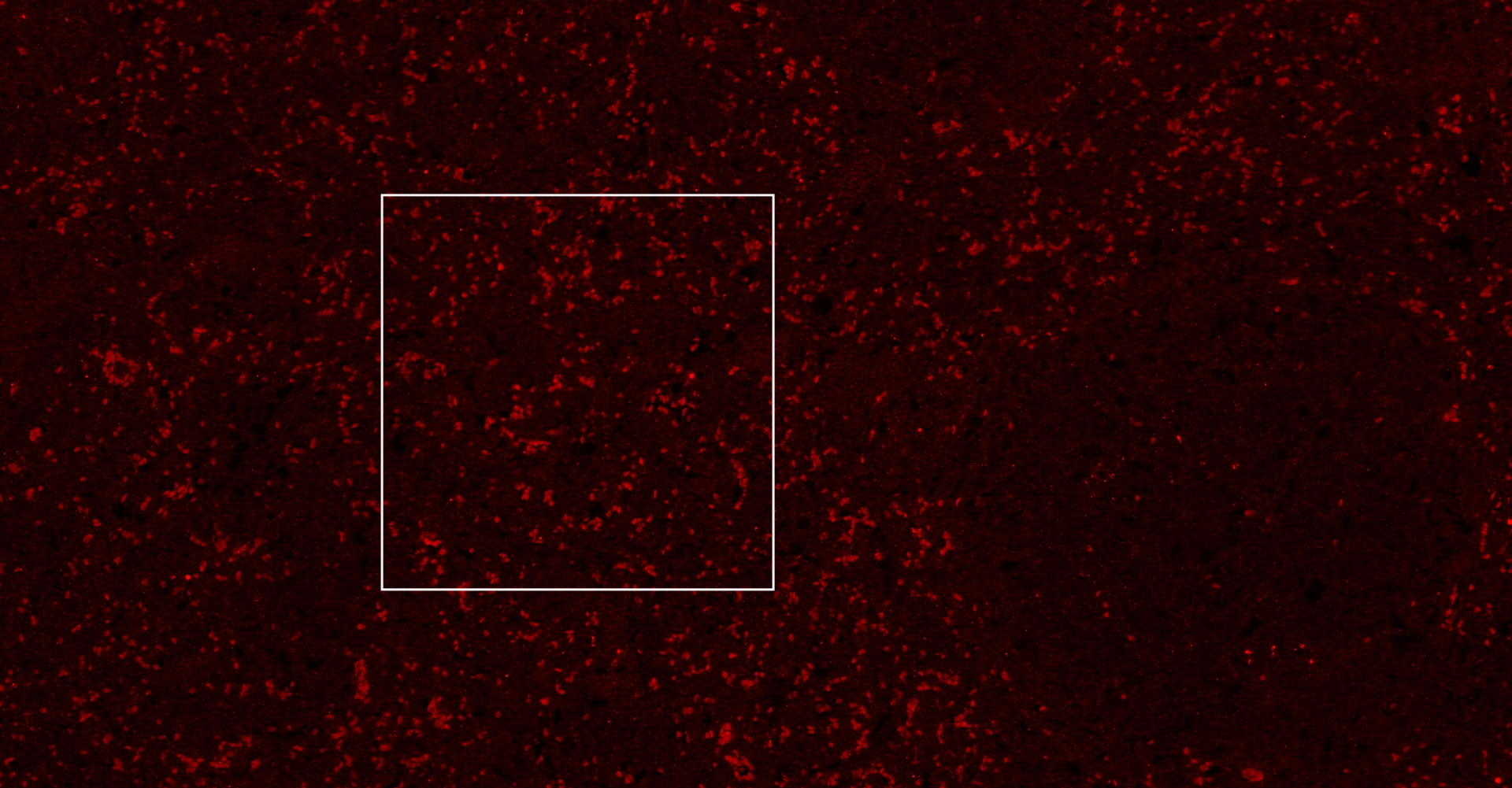

Supplement: Supplementary file 9 — Source data Fig. 7 [file 44319_2025_541_MOESM9_ESM.zip › Figure 7/7J/WT_spleen sVEGFR1 20.0x.tiff]

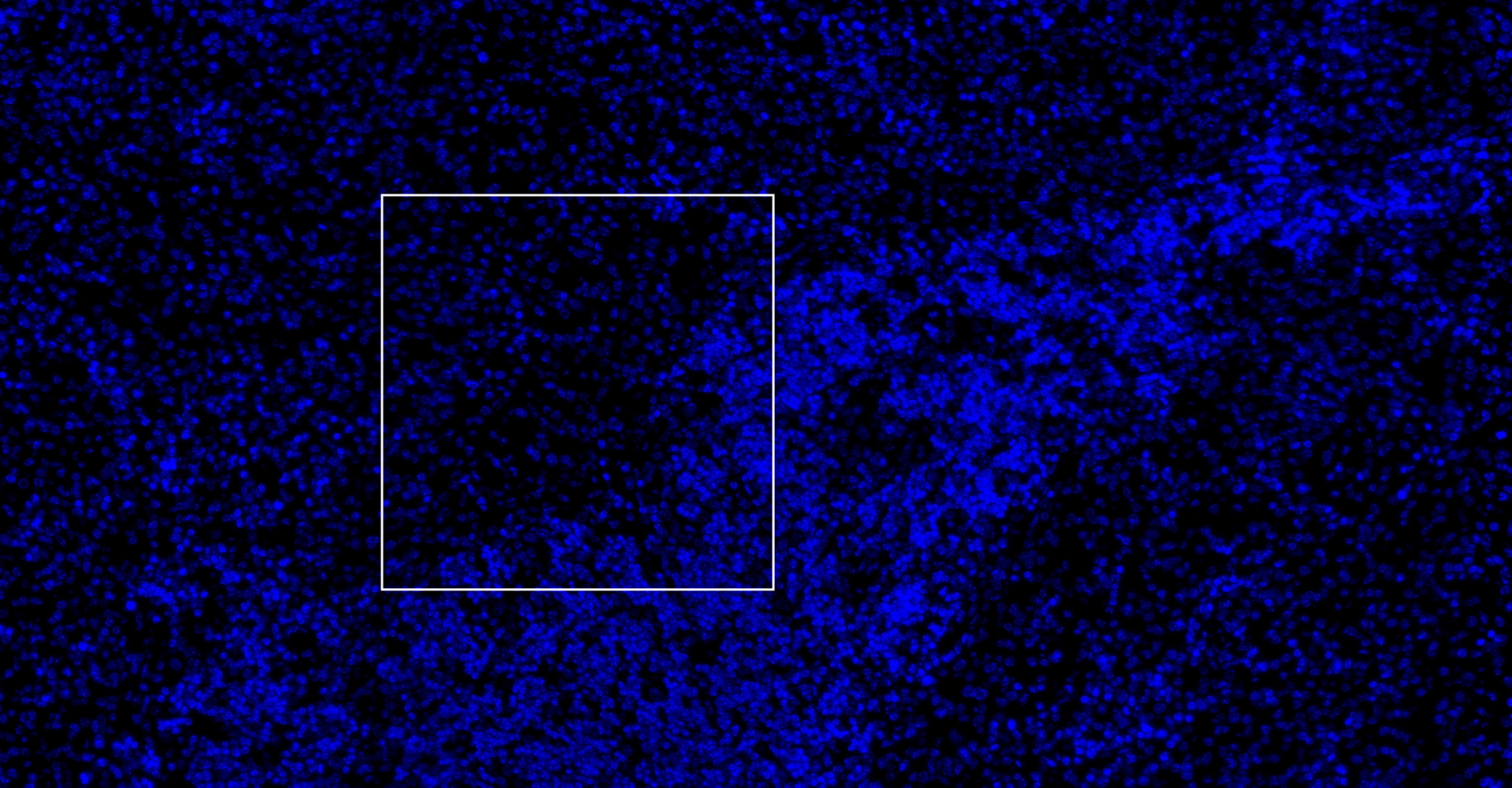

Supplement: Supplementary file 9 — Source data Fig. 7 [file 44319_2025_541_MOESM9_ESM.zip › Figure 7/7J/KO_spleen dapi 20.0x.tiff]

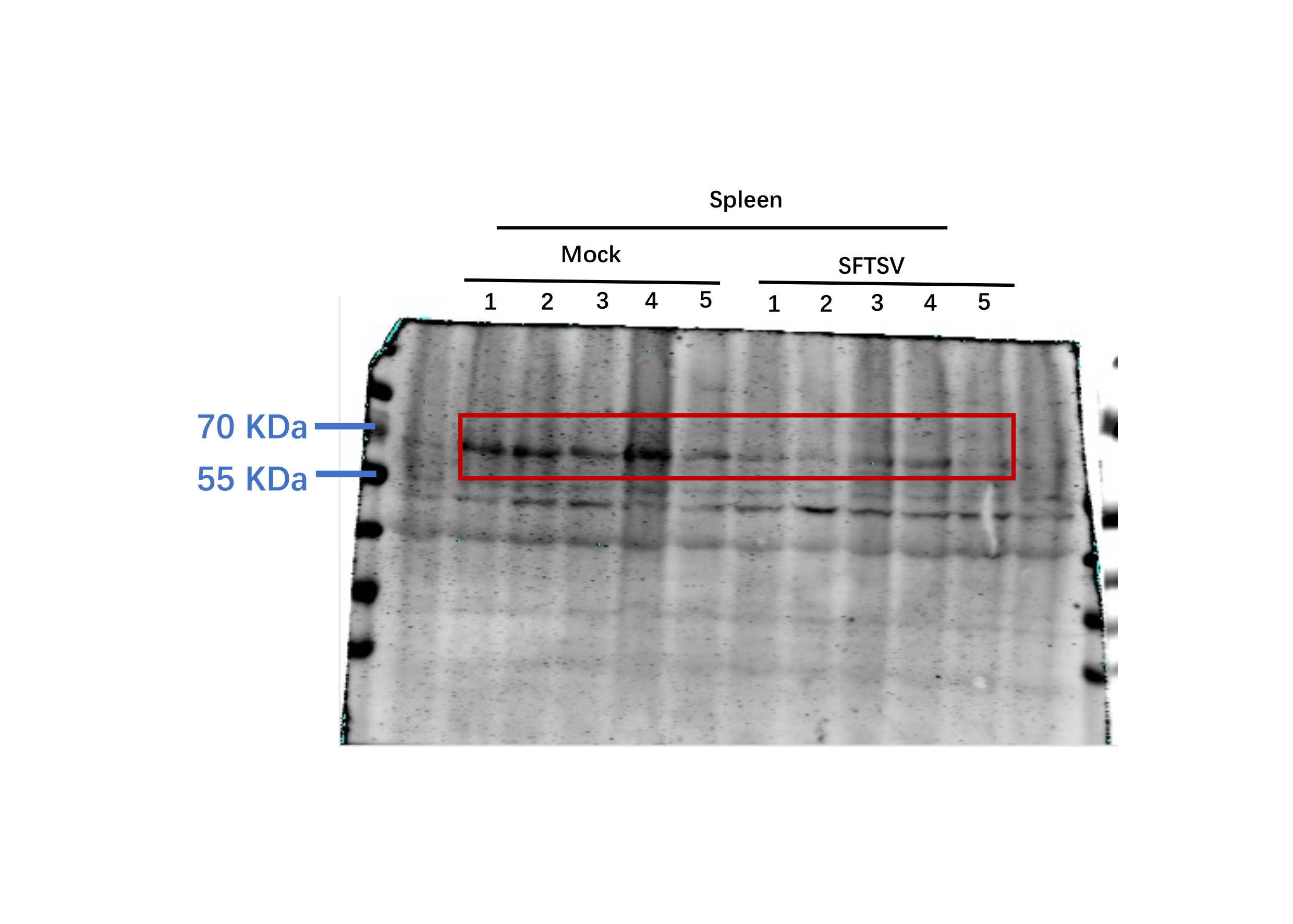

Supplement: Supplementary file 9 — Source data Fig. 7 [file 44319_2025_541_MOESM9_ESM.zip › Figure 7/7I/western sVEGFR1.tiff]

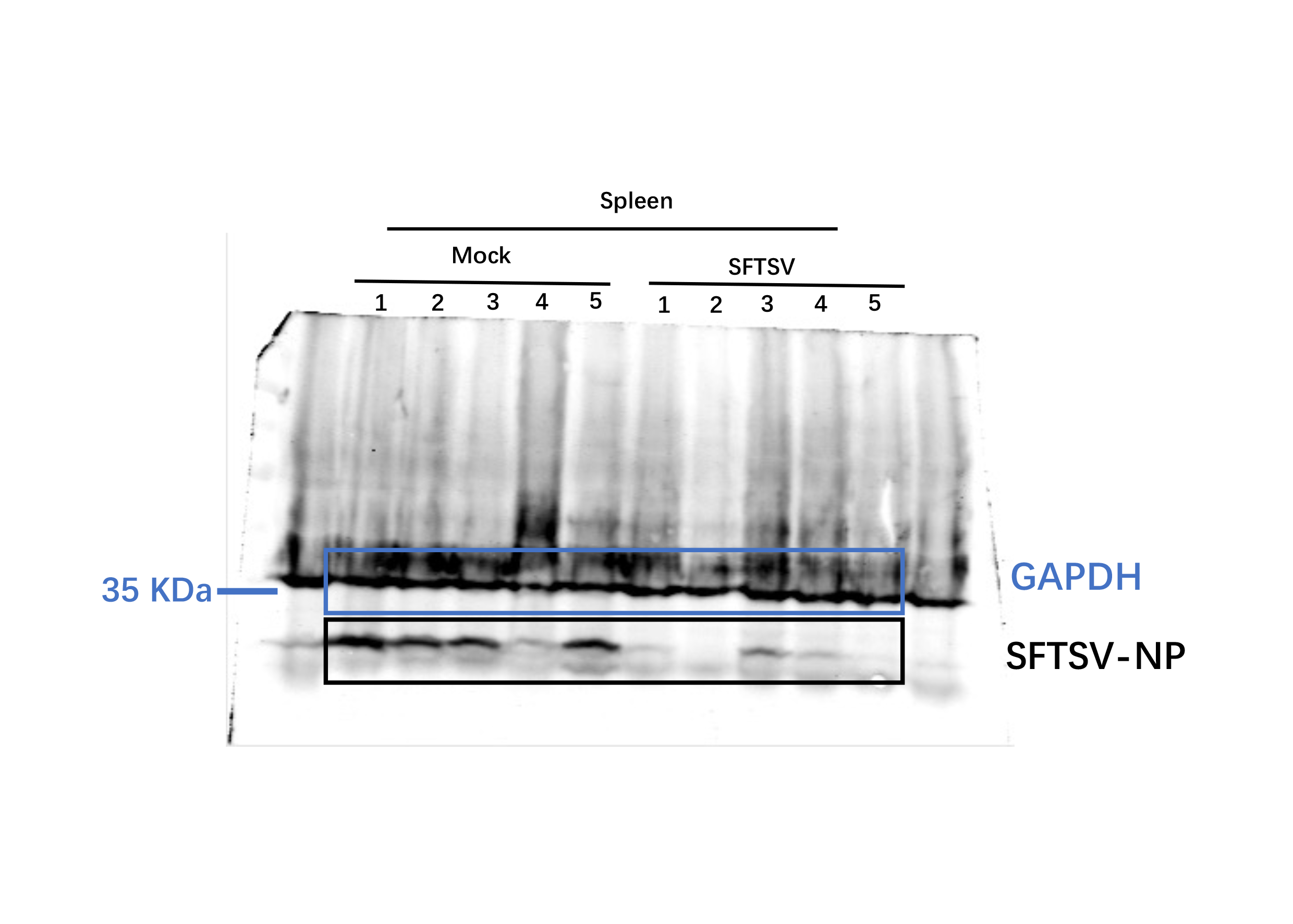

Supplement: Supplementary file 9 — Source data Fig. 7 [file 44319_2025_541_MOESM9_ESM.zip › Figure 7/7I/western NP and GAPDH.tiff]

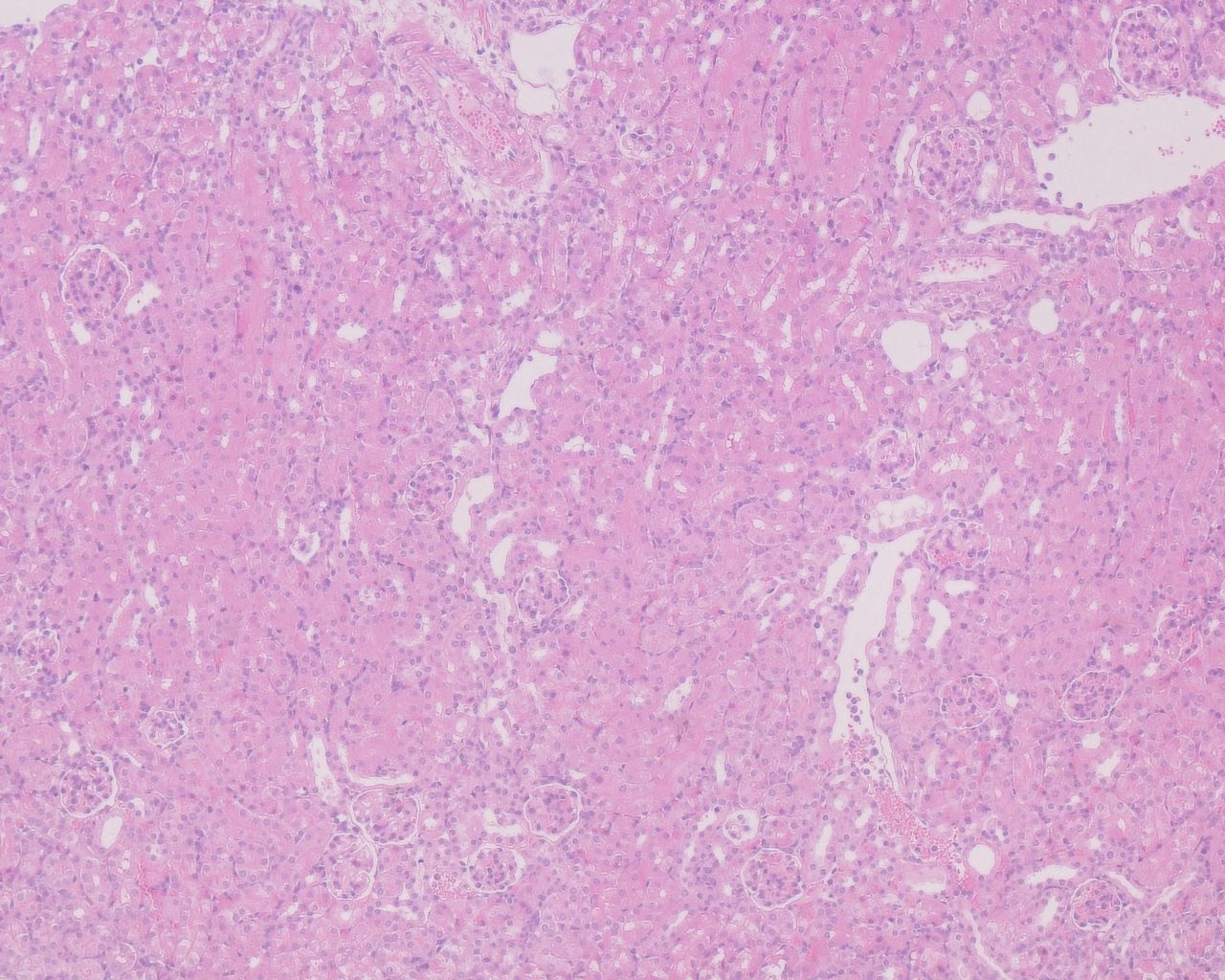

Supplement: Supplementary file 9 — Source data Fig. 7 [file 44319_2025_541_MOESM9_ESM.zip › Figure 7/7G/KO_kidney.tiff]

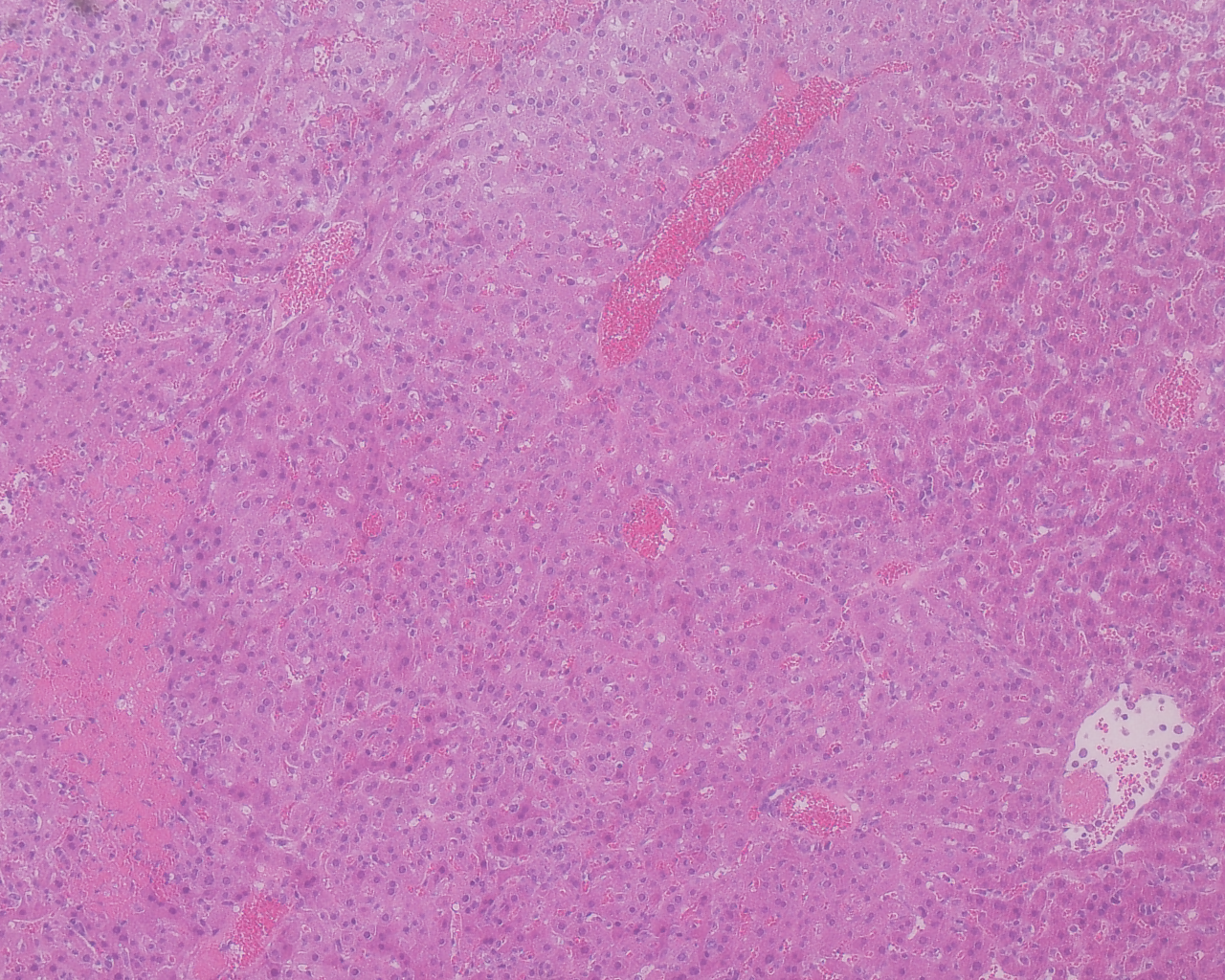

Supplement: Supplementary file 9 — Source data Fig. 7 [file 44319_2025_541_MOESM9_ESM.zip › Figure 7/7G/WT_liver.tiff]

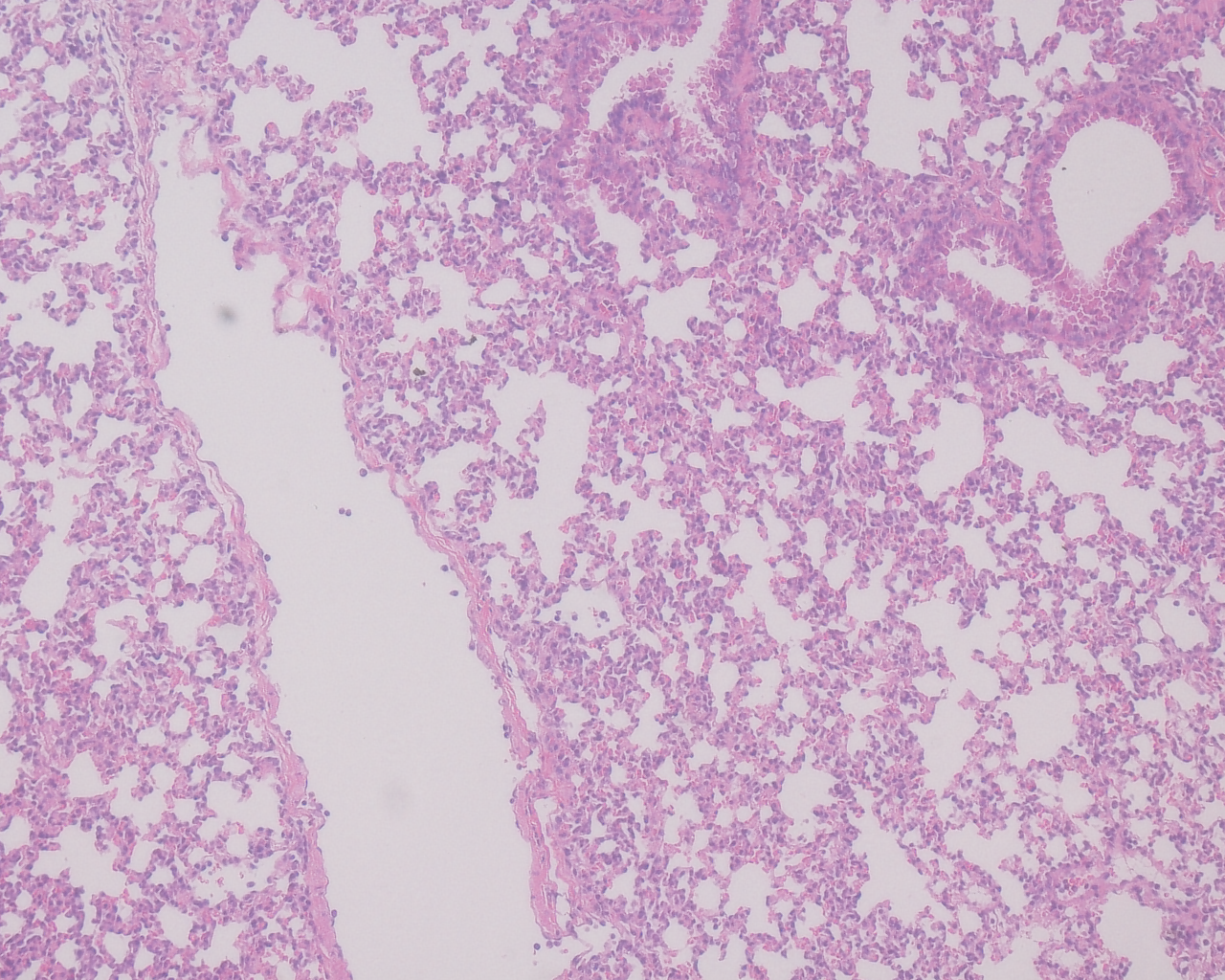

Supplement: Supplementary file 9 — Source data Fig. 7 [file 44319_2025_541_MOESM9_ESM.zip › Figure 7/7G/KO_lung.tiff]

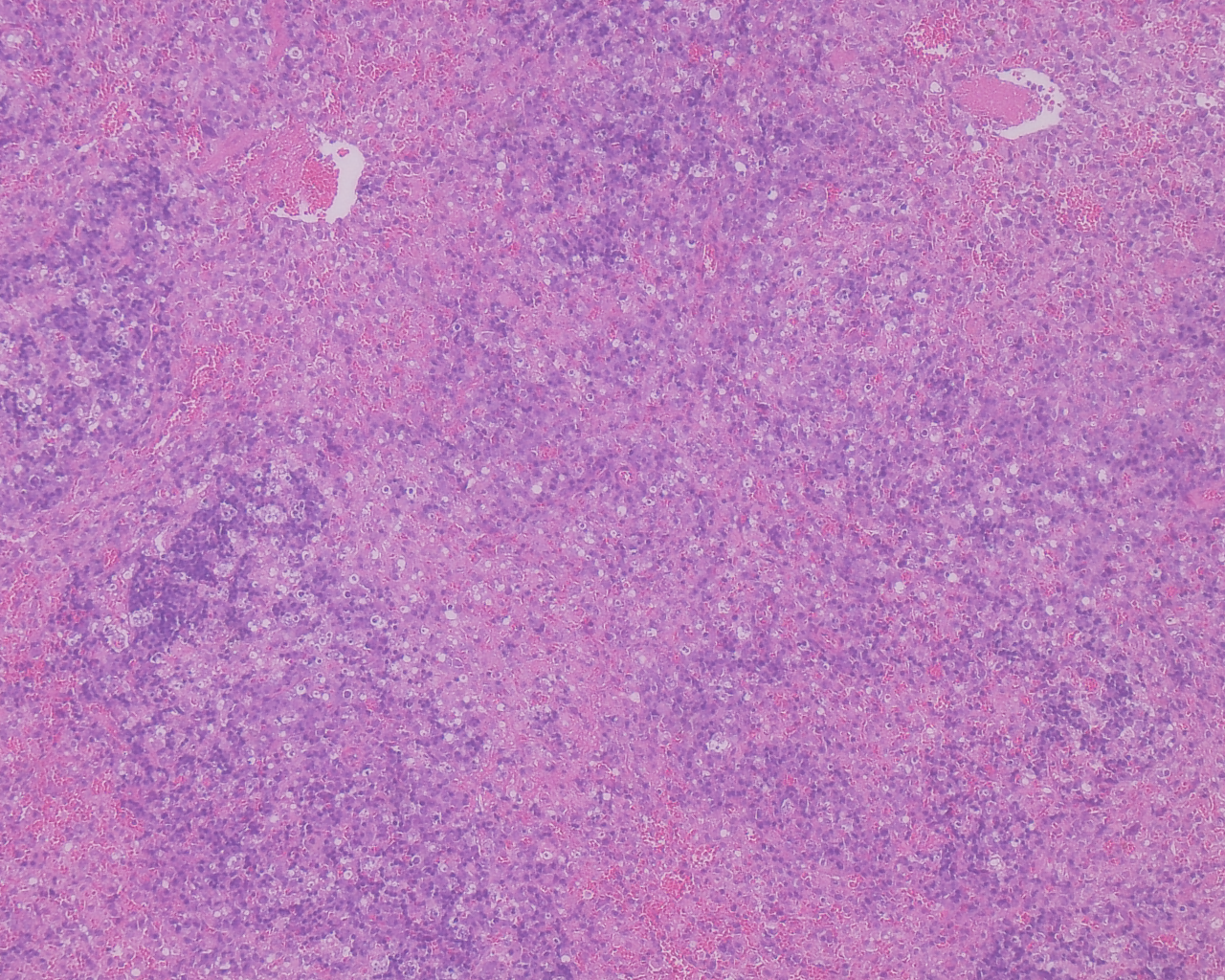

Supplement: Supplementary file 9 — Source data Fig. 7 [file 44319_2025_541_MOESM9_ESM.zip › Figure 7/7G/WT_spleen.tiff]

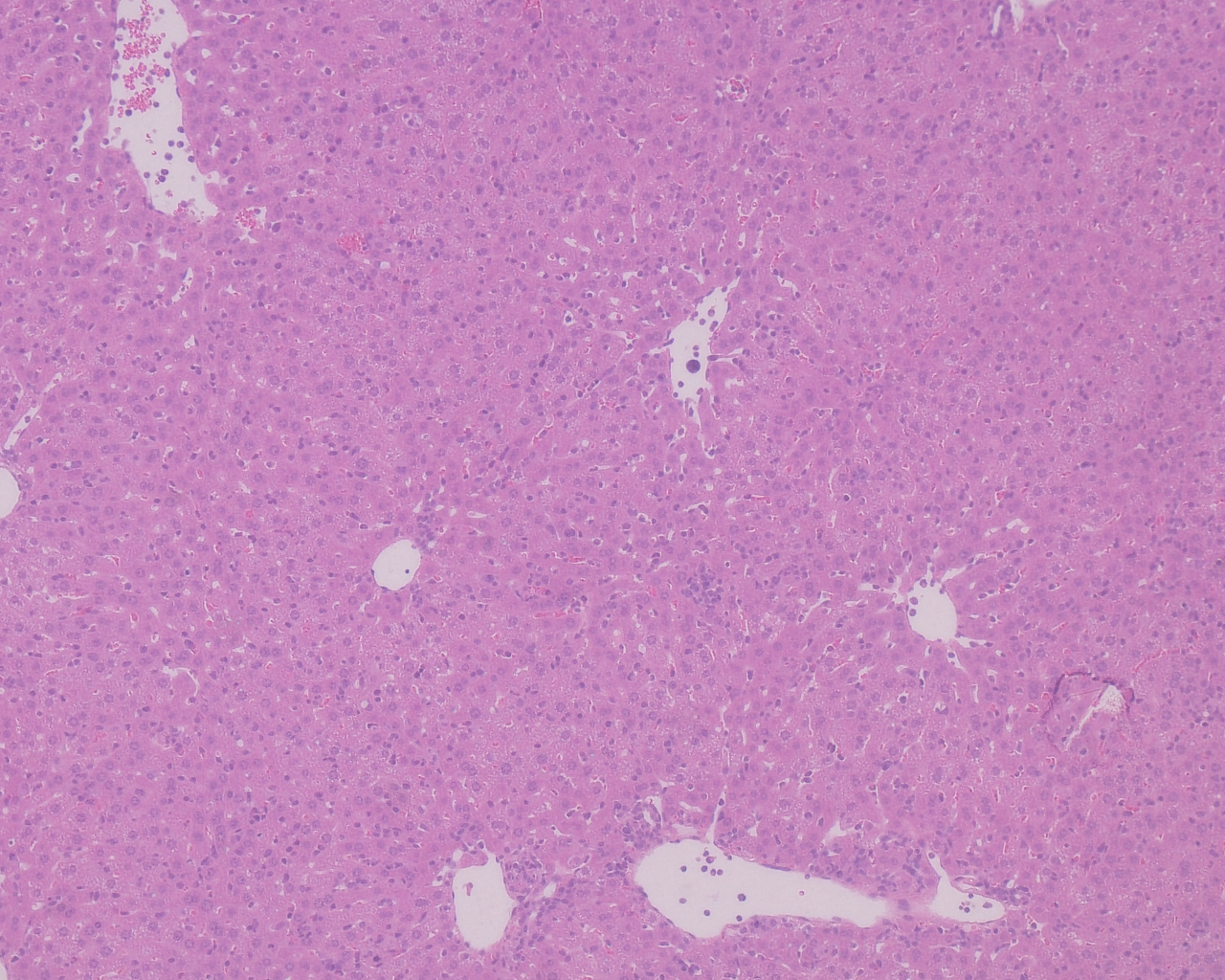

Supplement: Supplementary file 9 — Source data Fig. 7 [file 44319_2025_541_MOESM9_ESM.zip › Figure 7/7G/KO_liver.tiff]

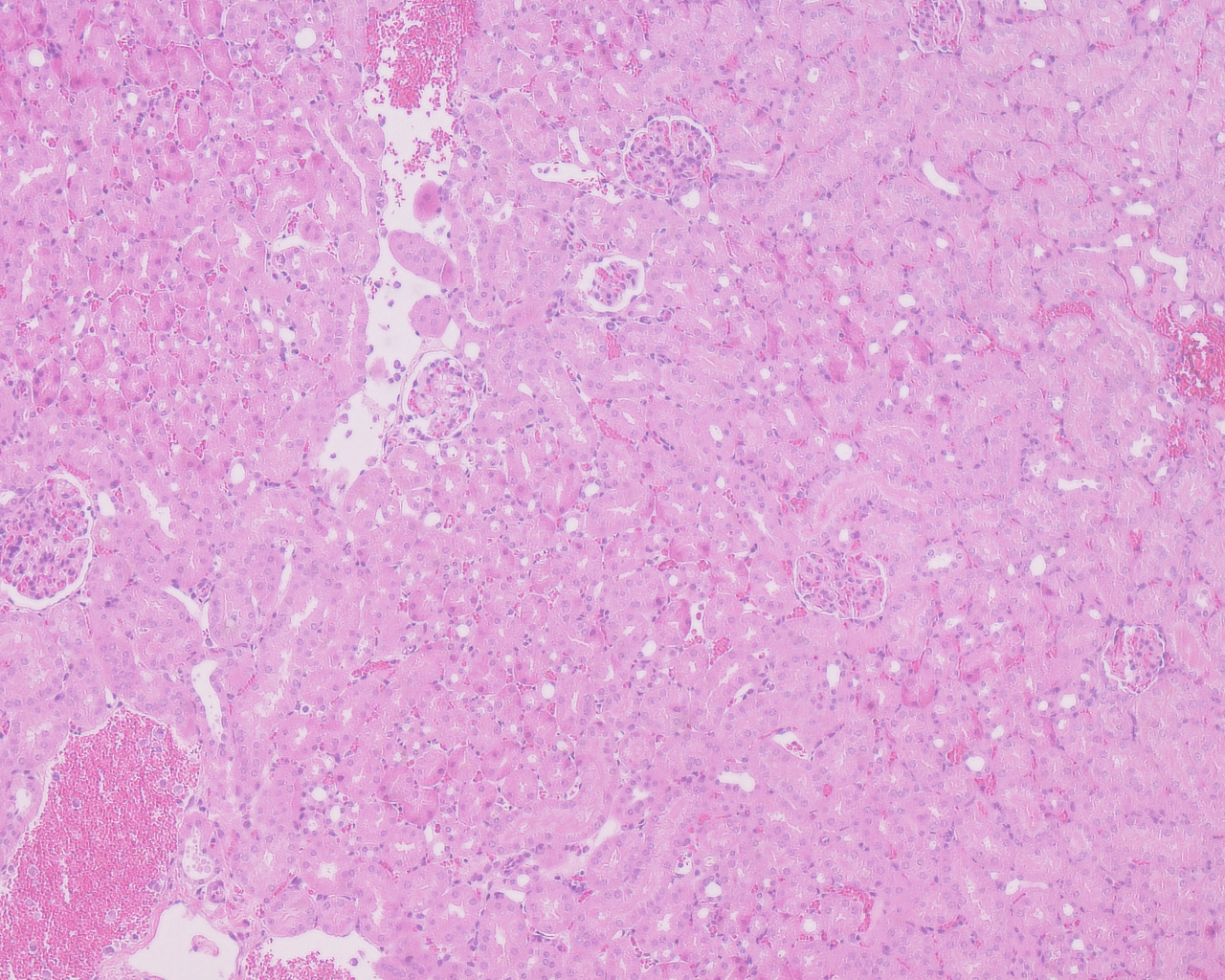

Supplement: Supplementary file 9 — Source data Fig. 7 [file 44319_2025_541_MOESM9_ESM.zip › Figure 7/7G/WT_kidney.tiff]

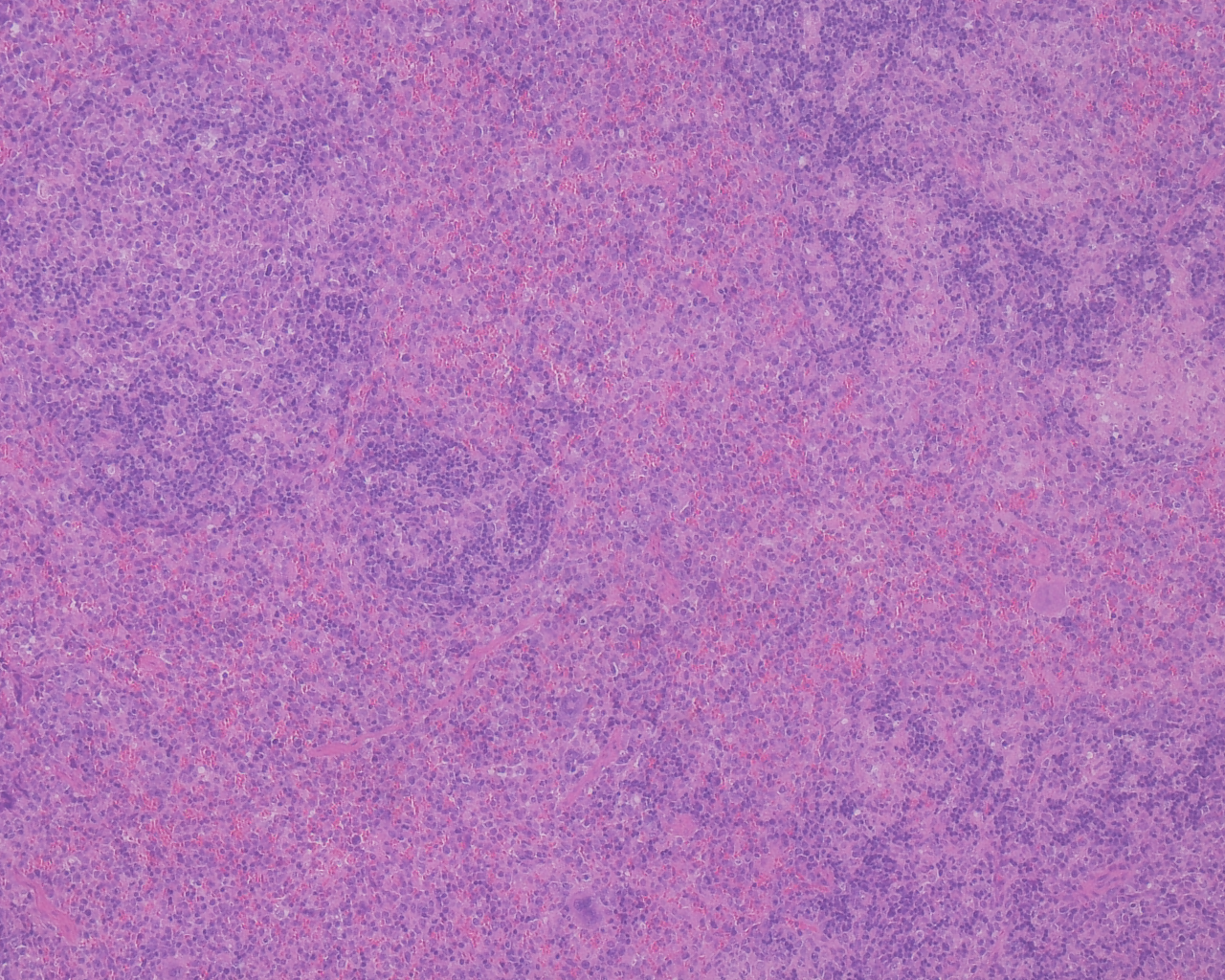

Supplement: Supplementary file 9 — Source data Fig. 7 [file 44319_2025_541_MOESM9_ESM.zip › Figure 7/7G/KO_spleen.tiff]

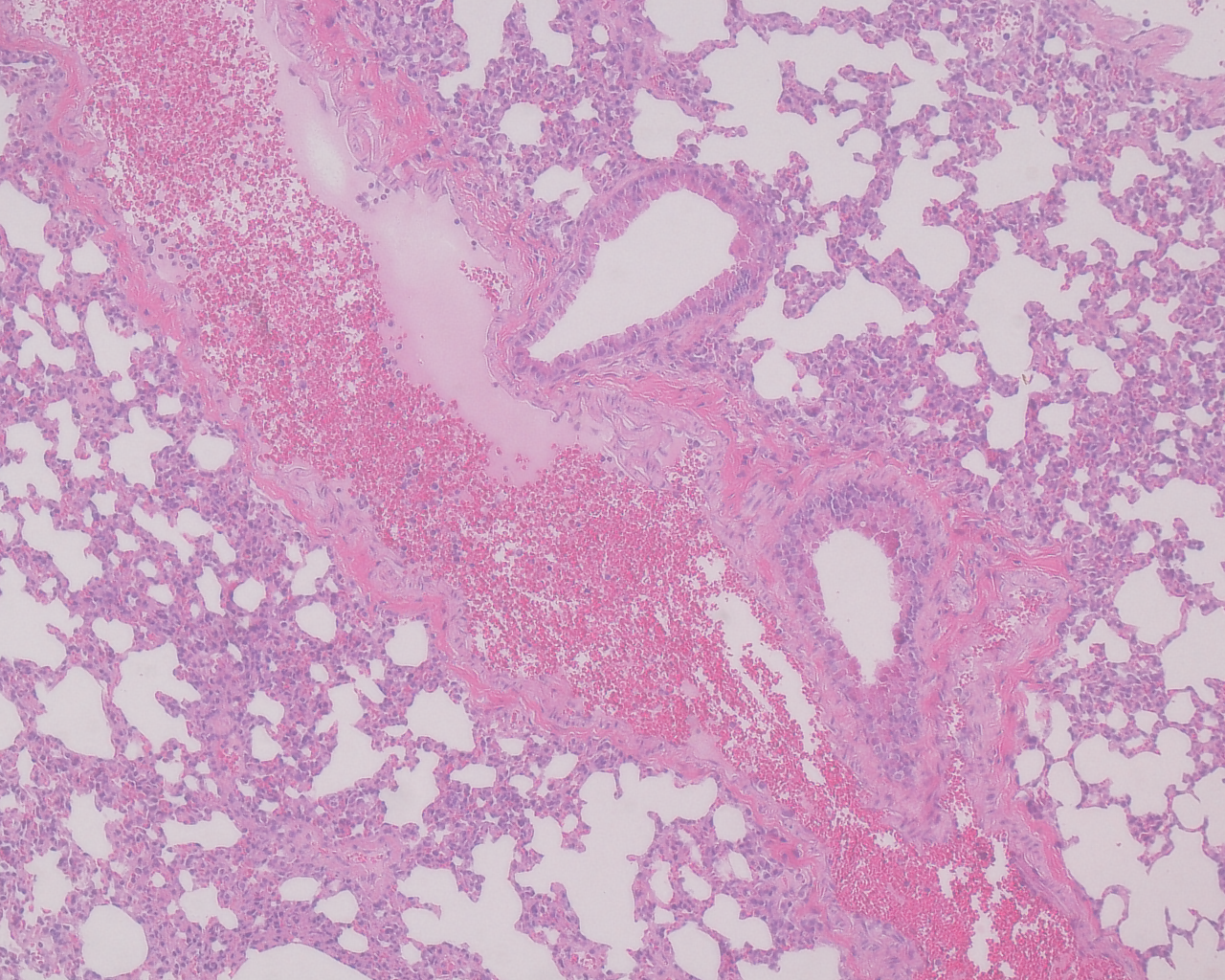

Supplement: Supplementary file 9 — Source data Fig. 7 [file 44319_2025_541_MOESM9_ESM.zip › Figure 7/7G/WT_lung.tiff]

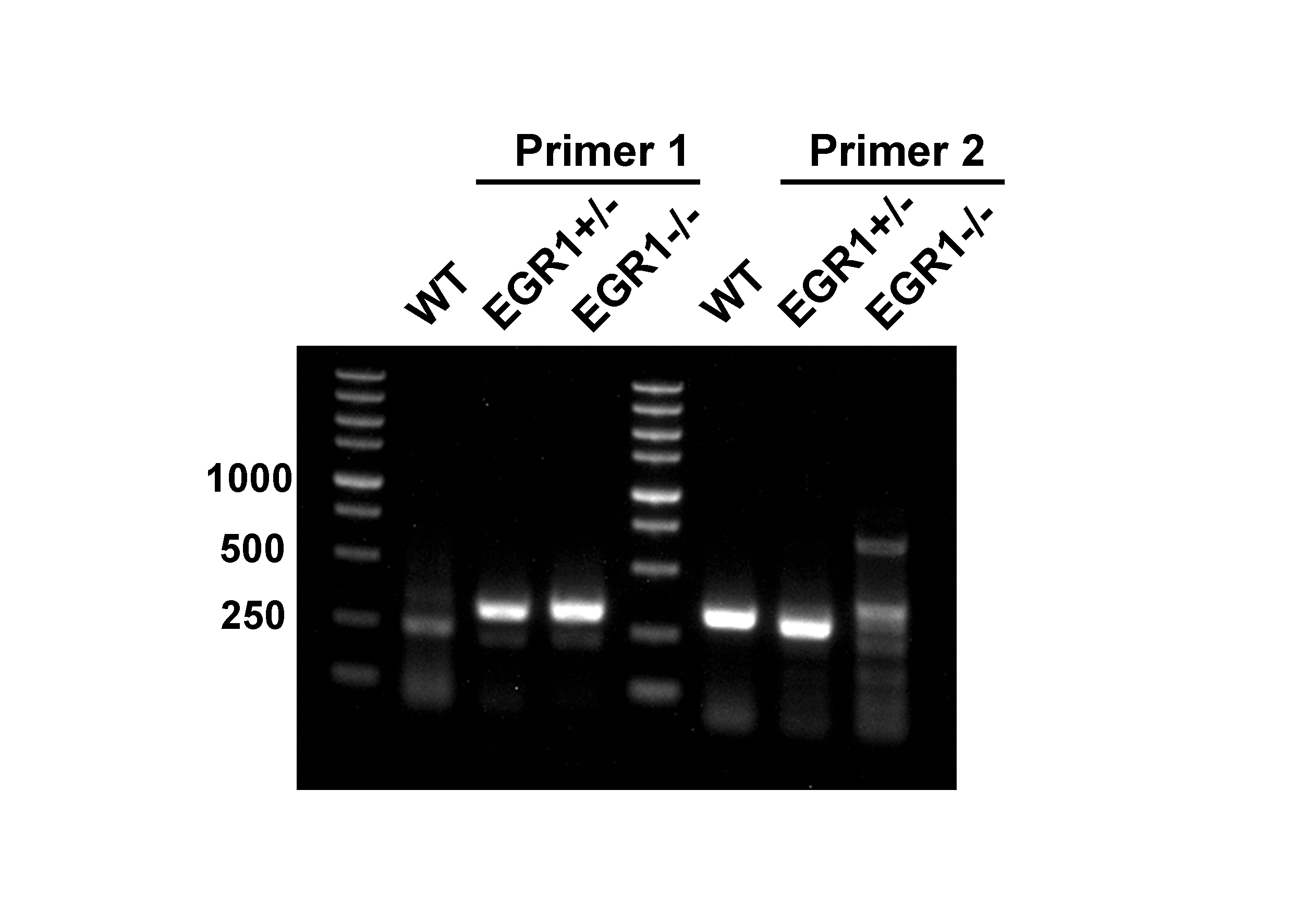

Supplement: Supplementary file 9 — Source data Fig. 7 [file 44319_2025_541_MOESM9_ESM.zip › Figure 7/7A/gel.tiff]
