# Supplementary material for: sVEGFR1 up-regulation via EGR1 impairs vascular repair in SFTSV-induced hemorrhage
Source: EMBO Rep. 2025 Aug 11;26(18):4477–502. doi: 10.1038/s44319-025-00541-2 (PMC12457690; doi:10.1038/s44319-025-00541-2)
Supplement: Supplementary file 10 — Expanded View Figures [file 44319_2025_541_MOESM10_ESM.pdf]

Expanded View Figures

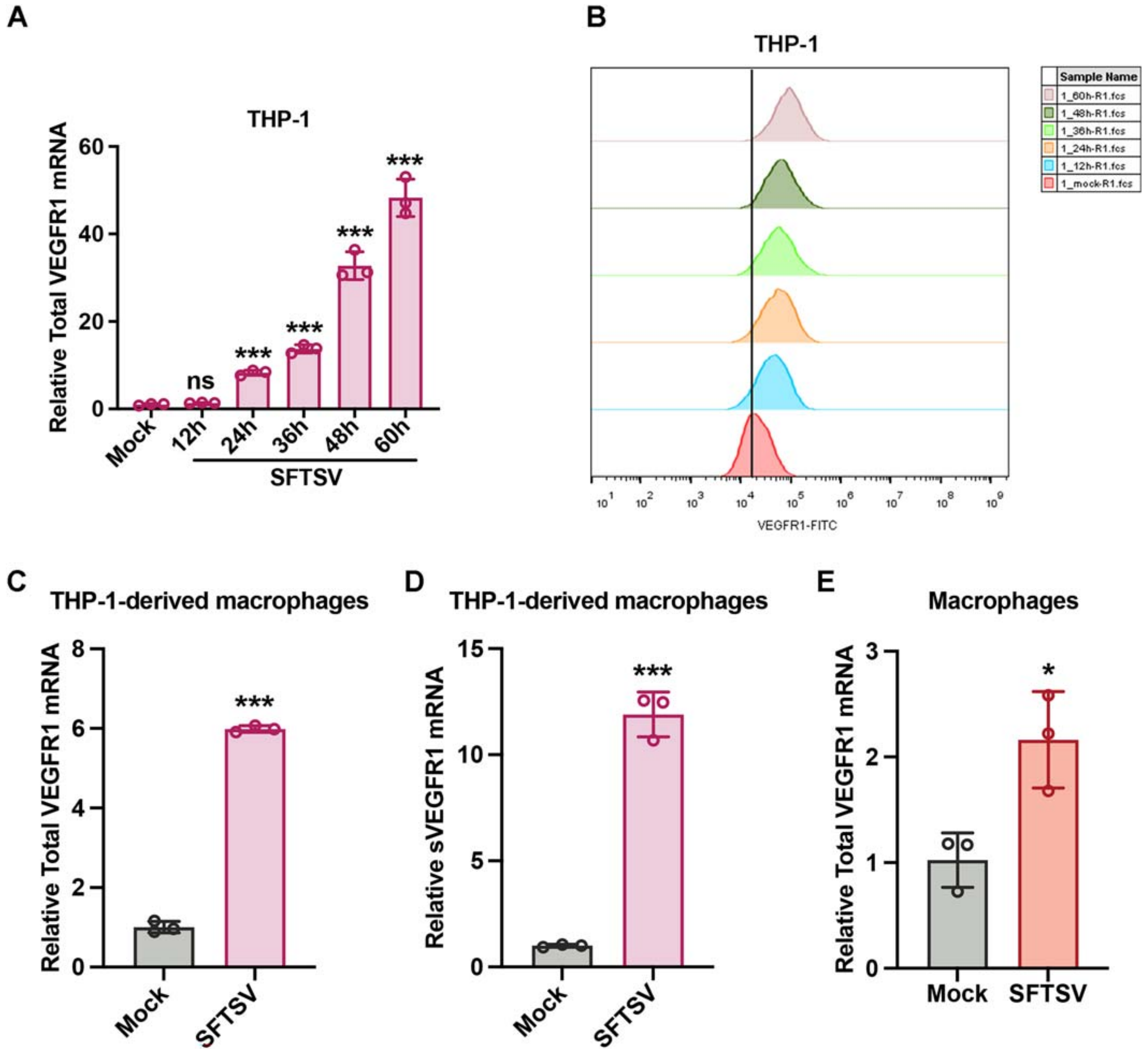

Figure EV1. SFTSV infection up-regulates total VEGFR1 and membrane-bound VEGFR1 expression.

(**A**) THP-1 cells were infected with SFTSV for 12, 24, 36, 48, and 60 h. Total VEGFR1 mRNA was measured by qPCR. $n = 3$ biological replicates. Statistical significance was determined by two-tailed unpaired Student t test. (ns = 0.0548, $***P < 0.0001$, $***P < 0.0001$, $***P < 0.0001$, $***P < 0.0001$) (**B**) THP-1 cells were infected with SFTSV for 12, 24, 36, 48, and 60 h. Then the cells were incubated with anti-VEGFR1 for 1 h at 4 °C and VEGFR1 level on the cell membrane was determined by flow cytometry. X-axes show anti-VEGFR1 antibody (logarithm of fluorescence), *Y* axes depict the cell count. (**C**, **D**) THP-1 cells were induced to differentiate into macrophage-like cells by 150 nM phorbol-12-myristate-13-acetate (PMA) and then infected with SFTSV. Total VEGFR1 (**C**) and sVEGFR1 (**D**) mRNAs were measured by qPCR. $n = 3$ biological replicates. Statistical significance was determined by two-tailed unpaired Student t test. ((**C**): $***P < 0.0001$; (**D**): $***P < 0.0001$). (**E**) Primary human monocyte-derived-macrophages were infected with SFTSV (MOI = 1) for 24 h. Total VEGFR1 mRNA was measured by qPCR. $n = 3$ biological replicates. Statistical significance was determined by two-tailed unpaired Student t test. ($*P = 0.0198$). Data information: Data shown are mean ± SD of three biological replicates. (ns, $P > 0.05$; $*P < 0.05$; $***P < 0.001$).

 © The Author(s)

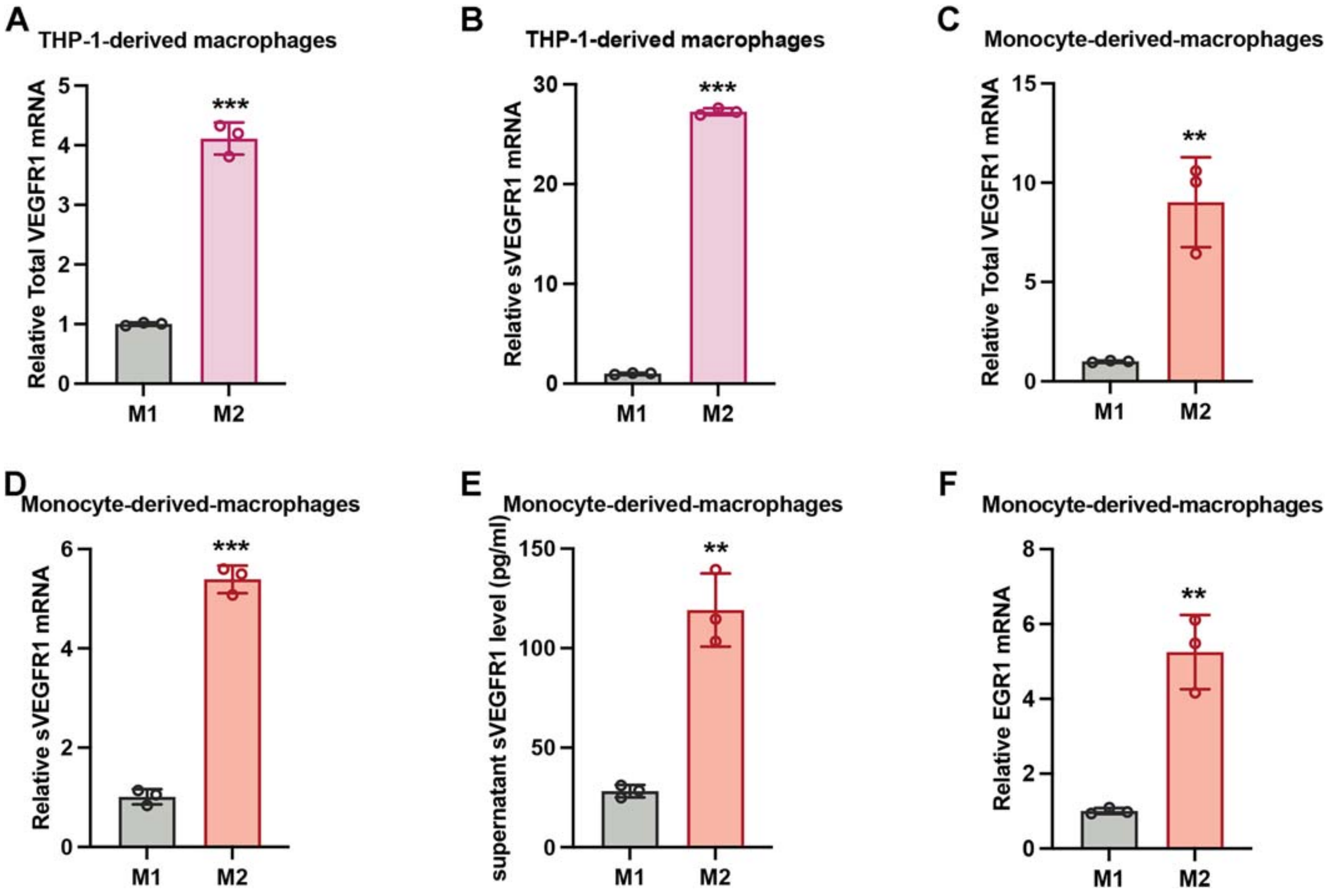

Figure EV2. sVEGFR1 is mainly produced by M2 macrophages.

(**A**, **B**) THP-1 cells were induced to differentiate into M1 macrophage-like cells by 24 h incubation with 150 nM PMA, followed by IFN-γ (20 ng/ml) and LPS (10 pg/ml), or THP-1 cells were induced to differentiate into M2 macrophage-like cells by 24 h incubation with 150 nM PMA, followed by interleukin 4 (20 ng/ml) and interleukin 13 (20 ng/ml) for 72 h. Total VEGFR1 (**A**) and sVEGFR1 (**B**) mRNAs were measured by qPCR. $n = 3$ biological replicates. Statistical significance was determined by two-tailed unpaired Student *t* test. ((**A**): $***P < 0.0001$; (**B**): $***P < 0.0001$). (**C**, **D**) Primary human monocyte-derived-macrophages were differentiated into M1 macrophages by culture in the presence of IFN-γ (20 ng/ml) and LPS (20 ng/ml) for 24 h, or were differentiated into M2 macrophages by culture in the presence of interleukin 4 (20 ng/ml) and interleukin 13 (20 ng/ml) for 72 h. Total VEGFR1 (**C**) and sVEGFR1 (**D**) were measured by qPCR. $n = 3$ biological replicates. Statistical significance was determined by two-tailed unpaired Student *t* test. ((**C**): $**P = 0.0036$; (**D**): $***P < 0.0001$). (**E**) Primary human monocyte-derived-macrophages were differentiated into M1 macrophages or M2 macrophages. The secreted sVEGFR1 in cell supernatant was measured by capture ELISA. $n = 3$ biological replicates. Statistical significance was determined by two-tailed unpaired Student *t* test. ($**P = 0.0011$). (**F**) Primary human monocyte-derived-macrophages were differentiated into M1 macrophages or M2 macrophages. EGR1 mRNA was measured by qPCR. $n = 3$ biological replicates. Statistical significance was determined by two-tailed unpaired Student *t* test. ($**P = 0.0018$). Data information: Data shown are mean ± SD of three biological replicates. ($**P < 0.01$; $***P < 0.001$).

© The Author(s)

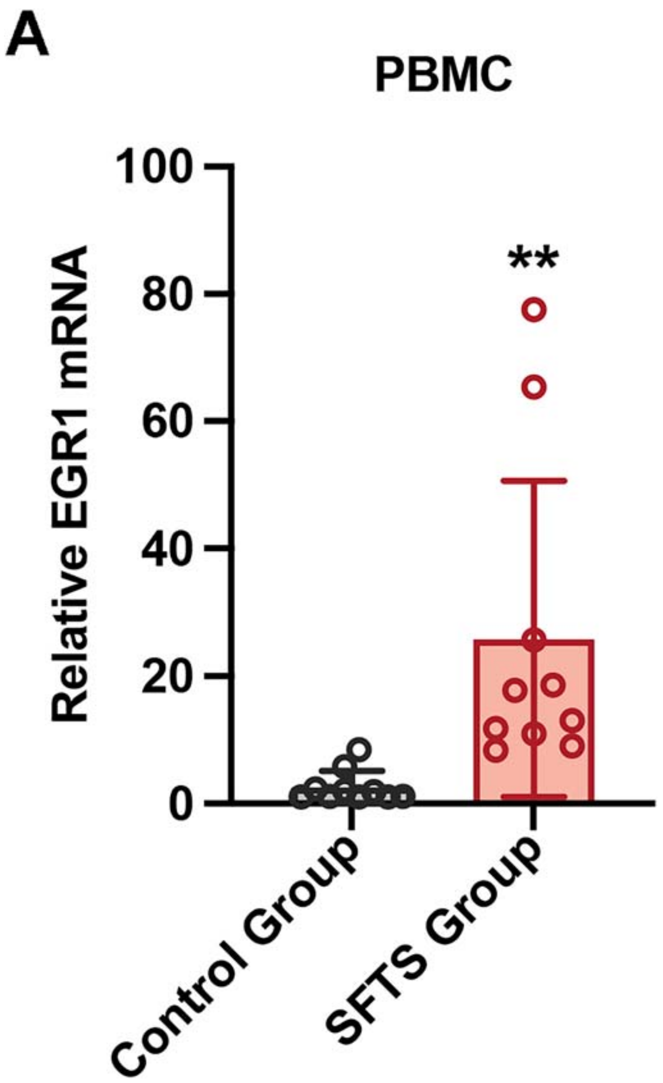

Figure EV3. SFTSV infection up-regulates EGR1 expression in SFTS patients.

(**A**) PBMCs were isolated from 10 SFTS patients and 10 healthy individuals as controls. EGR1 expression in PBMCs among SFTS patients were measured by qPCR. $n = 10$ biological replicates. Statistical significance was determined by two-tailed unpaired Student *t* test. (**$P = 0.0085$). Data information: Data shown are mean ± SD of ten biological replicates with each data point representing a biological experiment (**$P < 0.01$).

 © The Author(s)

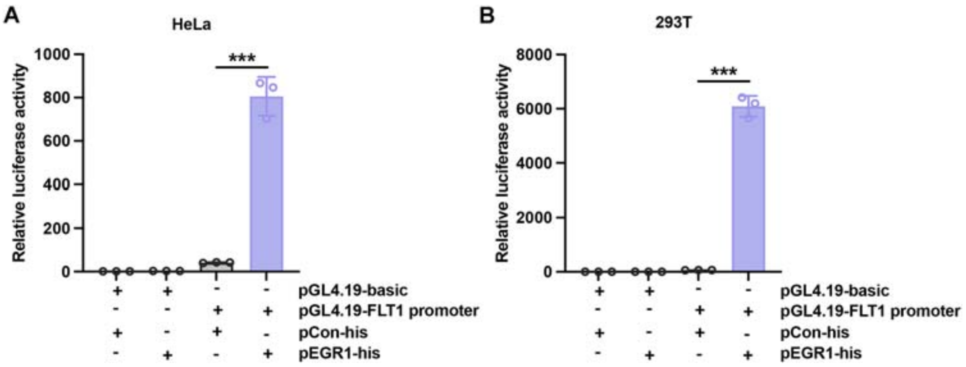

Figure EV4. Overexpression of EGR1 activates the VEGFR1 promoter.

(**A**, **B**) Luciferase reporter assay. For the VEGFR1 promoter luciferase reporter assay, the predicted binding sites of VEGFR1 and EGR1 were obtained from the eukaryotic promoter database (EPD). The corresponding promoter constructs in the promoter region of VEGFR1 was synthesized and then inserted into the pGL4.19-basic firefly luciferase reporter vector (PPL), named pGL4.19-FLT1 promoter. These vectors were co-transfected into HeLa or HEK-293T cells with EGR1 construct or empty vector. Measurement of luciferase activity was conducted at 48 h post-transfection. Representative results were from three independent experiments. $n = 3$ biological replicates. Statistical significance was determined by two-tailed unpaired Student *t* test. ((**A**): ***$P = 0.0001$; (**B**): ***$P < 0.0001$). Data information: Data shown are mean ± SD of three biological replicates (***$P < 0.001$).

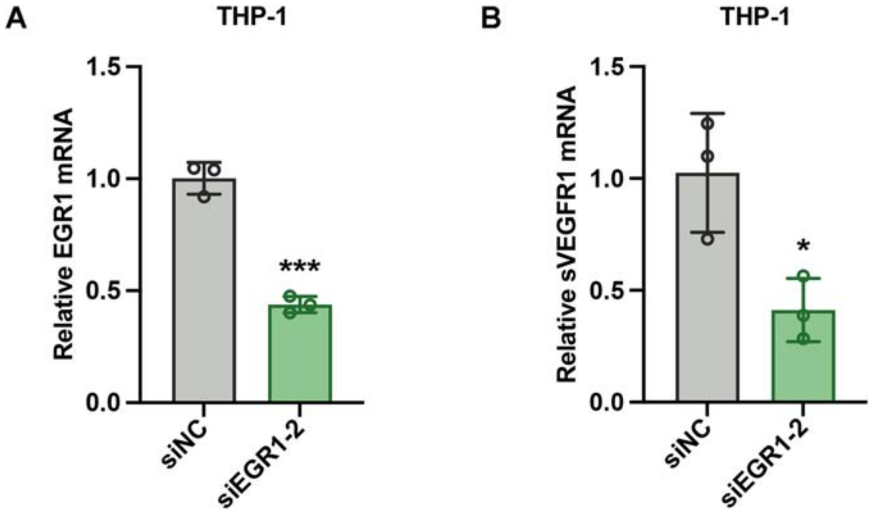

Figure EV5. Knock-down of EGR1 reduces sVEGFR1 expression.

(**A, B**) THP-1 cells transfected with siNC or siEGR1 (siEGR1-2 in Fig. 5G) were harvested at 36 hpt to evaluate EGR1 mRNA (**A**) knock-down efficiency and sVEGFR1 mRNA (**B**) levels by qPCR. $n = 3$ biological replicates. Statistical significance was determined by two-tailed unpaired Student t test. ((**A**): ***$P = 0.0003$; (**B**): *$P = 0.0243$). Data information: Data shown are mean ± SD of three biological replicates (*$P < 0.05$; ***$P < 0.001$).

 © The Author(s)
